# Multicenter phase 1/2 study of onatasertib, a dual TORC1/2 inhibitor, combined with the PD-1 antibody toripalimab in advanced solid tumors

**DOI:** 10.1038/s41392-025-02281-0

**Published:** 2025-06-25

**Authors:** Pei Shu, Xiaoyu Li, Qi Zhou, Guiling Li, Keqiang Zhang, Li Yuan, Yixian Liu, Qiu Li, Yongsheng Wang, Hui Xie, Li Zheng

**Affiliations:** 1 Cancer Center, West China Hospital, Sichuan University, Chengdu, China; 2 Clinical Trial Center, National Medical Products Administration Key Laboratory for Clinical Research and Evaluation of Innovative Drugs, West China Hospital, Sichuan University, Chengdu, China; 3 Gynecological Oncology Center, Chongqing University Cancer Hospital, Chongqing, China; 4 Cancer Center, Union Hospital Tongji Medical College, Huazhong University of Science and Technology, Wuhan, China; 5 Department of Gynecological Oncology, Hunan Cancer Hospital, Changsha, China; 6 Department of Clinical Research and Development, Antengene Corporation, Shanghai, China

**Keywords:** Drug development, Gynaecological cancer

## Abstract

Preclinical studies have indicated that the combination of mTORC1/2 inhibitors with PD-1 antibodies exhibits synergistic effects on solid tumors. However, no clinical data supporting this combination have been reported. Therefore, we conducted a clinical trial (NCT 04337463) to investigate the efficacy and safety of combining onatasertib, an mTORC1/2 inhibitor, with toripalimab, a PD-1 antibody in patients with advanced solid tumors. This open-label, phase 1/2 clinical trial included dose escalation and dose expansion cohorts to evaluate safety, tolerability, objective response rate (ORR), disease control rate (DCR) and progression-free survival (PFS). A total of 46 patients were enrolled and received onatasertib at doses of 15 mg, 20 mg, or 30 mg once daily (QD), combined with toripalimab 240 mg every 3 weeks (Q3W). No dose-limiting toxicities were observed, and the most common grade 3 or 4 treatment emergent adverse events were lymphopenia (23.9%) and rash (19.6%). The overall ORR was 26.1%, with a DCR of 73.9%, and a median PFS of 4.3 months. In cervical cancer patients, regardless of PD-L1 expression, the ORR was 52.4%, DCR was 90.5% and median PFS was 5.8 months. Notably, the 15 mg combination dose demonstrated a median PFS of 7.8 months. In conclusion, the safety profile of onatasertib in combination with toripalimab was manageable and showed encouraging clinical activity in advanced solid tumors, particularly among cervical cancer patients, irrespective of PD-L1 expression. The recommended phase 2 dose for the combination was determined to be onatasertib 15 mg QD and toripalimab 240 mg Q3W.

## Introduction

Immune checkpoint inhibitors (CPIs), such as PD-1 or PD-L1 antibodies, have significantly impacted treatment outcomes across various solid tumor malignancies.^1^ However, only a subset of patients achieves long-term therapeutic benefit.^2^ Therefore, novel effective therapies involving diverse antitumor mechanisms are needed to enhance the efficacy of PD-1/L1-based immunotherapy. Preclinical studies have suggested that combining mTOR inhibitors with PD-1 antibodies has synergistic effects across multiple tumor types, including melanoma, hepatocellular carcinoma, head and neck cancer, and others.^3–5^ Thus, there is a strong rationale to combine these two treatment modalities to improve and prolong antitumor responses in patients with advanced cancer.

The mammalian target of rapamycin (mTOR) is highly conserved serine/threonine kinase that functions as a central regulator of cell growth, integrating signals from mitogen, energy, and nutrient. It operates downstream of the phosphatidylinositol 3 kinase (PI3K)-protein kinase B (AKT) pathway and executes its biologic functions through two distinct complexes, mTORC1 and mTORC2.^6,7^ In many cancers, aberrant activation of the PI3K-AKT-mTOR pathway plays a crucial role in cancer cell proliferation and survival. In renal cell carcinoma (RCC), the mTOR pathway has been reported to regulate hypoxia-inducible factor (HIF) protein expression under specific cellular conditions. The inappropriate accumulation of HIF-1α and HIF-2α, caused by biallelic mutations in the von Hippel-Lindau (VHL) gene observed in the majority of clear cell RCC cases, is considered a pivotal step in RCC tumorigenesis.^8^ Neuroendocrine tumors (NETs) are associated with some rare familial syndromes, which result from mutations in genes linked to the PI3K-AKT-mTOR pathway, including Multiple Endocrine Neoplasia-1 (MEN1),^9^ Neurofibromatosis-1 (NF1),^10^ VHL^11^ and PTEN.^12^ Clinical efficacy of rapamycin analog (rapalog), everolimus, has been demonstrated in RCC and certain NETS. In cervical cancer, Xu et al. reported frequent alterations in the PI3K-AKT pathway, with PIK3CA being the most commonly mutated gene, occurring at an incidence of 31.7%.^13^ These findings suggest that the PI3K-AKT-mTOR pathway represents a classical therapeutic target, and mTOR inhibitors are currently being developed as potential therapeutic agents for various cancers.^7,14^

First-generation allosteric (non-competitive) mTOR inhibitors, such as everolimus and temsirolimus, which specifically target mTORC1, have been approved for the treatment of advanced RCC. Additionally, everolimus has received approval for the treatment of HER2-negative breast cancer, subependymal giant cell astrocytoma (SEGA), and progressive or metastatic pancreatic, gastrointestinal, and lung NETs.^15^ However, first-generation mTOR inhibitors exhibited certain limitations due to their exclusive targeting of mTORC1, which induced feedback activation of AKT and showing resistance to mTORC2.^16,17^ Second-generation ATP-competitive mTOR inhibitors, capable of simultaneously suppressing both mTORC1 and mTORC2, can overcome the feedback inhibition observed with mTORC1 inhibitors alone.^18^ Furthermore, second-generation mTOR inhibitors have been reported to exhibit enhanced potency and selectivity; some of these inhibitors also target PI3K in addition to inhibiting mTORC1 and mTORC2.^19^ Beyond their direct tumor-suppressive effects, both first and second generation mTOR inhibitors were reported to regulate immunity,^3,4^ including altering PD-L1 expression via the AKT-mTOR pathway activation,^20^ and enhance tumor sensitivity to immunotherapy by modifying the tumor microenvironment (TME).^5,6,21^

Onatasertib (also known as ATG-008 or CC-223), a selective mTOR kinase inhibitor targeting both mTORC1 and mTORC2, has shown inhibitory effects on hepatocellular carcinoma, neuroendocrine tumors, and other tumor types.^22,23^ It exhibits an acceptable and manageable safety profile, with a maximum tolerable dose of 45 mg once daily (QD).^22,23^ Due to its unique and broad mTORC1/2 inhibition profile, ability to modulate the TME, and direct antitumor activities, onatasertib was considered as a suitable candidate for combination therapy with immunotherapy.

Toripalimab, a humanized IgG4κ monoclonal antibody specific for human PD-1, has received National Medical Product Administration (NMPA) approval for use as a monotherapy in melanoma, advanced nasopharyngeal carcinoma, and metastatic urothelial carcinoma in China, and US Food and Drug Administration (FDA) approval for nasopharyngeal carcinoma treatment.^24–27^ Clinical data from patients with solid tumors have shown that toripalimab is generally well tolerated, with an approved dose of 240 mg administered intravenously every three weeks (Q3W).^28^ Notably, no prior clinical studies reported the combination of a TORC1/2 inhibitor with immune checkpoint inhibitors. Here, we present the findings from TORCH-2 study, a nonrandomized phase 1/2 trial, investigating the safety, tolerability, and efficacy of onatasertib in combination with toripalimab in patients with advanced solid tumors.

## Results

### Patients

**Fig. 1:**
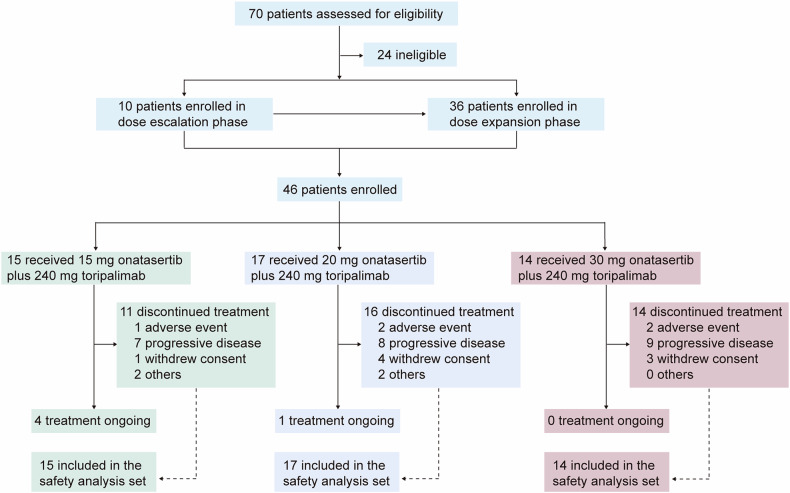
Study flowchart

Between April 23, 2020, and October 21, 2022, a total of 70 patients were screened for eligibility, with 46 being enrolled in the study (Fig. 1). Patients received onatasertib at doses of 15 mg (*n* = 15), 20 mg (*n* = 17), and 30 mg (*n* = 14) QD, along with 240 mg of toripalimab administered Q3W. Demographic information is summarized in Table 1. As of the cutoff date of October 20, 2023, the median follow-up period was 27.3 months. Of the 46 patients, 41 completed treatments, while five remained on active treatment.

**Table 1:** Patient demographics and baseline characteristics

	Onatasertib 15 mg + Toripalimab 240 mg (*n* = 15)	Onatasertib 20 mg + Toripalimab 240 mg (*n* = 17)	Onatasertib 30 mg + Toripalimab 240 mg (*n* = 14)	All enrolled patients (*n* = 46)
Age, years	55 (35–70)	53 (34–58)	50 (25–64)	53 (25–70)
Sex				
Women	11 (73.3)	13 (76.5)	9 (64.3)	33 (71.7)
Men	4 (26.7)	4 (23.5)	5 (35.7)	13 (28.3)
Primary site of tumor				
Liver	1 (6.7)	2 (11.8)	2 (14.3)	5 (10.9)
Ovary	0	2 (11.8)	2 (14.3)	4 (8.7)
Cervix	10 (66.7)	8 (47.1)	3 (21.4)	21 (45.7)
Others	4 (26.7)	5 (29.4)	7 (50.0)	16 (34.8)
ECOG performance status				
0	3 (20.0)	7 (41.2)	1 (7.1)	11 (23.9)
1	12 (80.0)	10 (58.8)	13 (92.9)	35 (76.1)
PD-L1 status				
Positive	4 (26.7)	6 (35.3)	7 (50)	17 (34.8)
Negative	8 (53.3)	10 (52.9)	7 (50)	25 (54.3)
Missing	3 (20)	1 (5.9)	0	4 (8.7)
Disease state at enrollment				
Metastatic	14 (93.3)	12 (70.6)	13 (92.9)	39 (84.8)
Non metastatic	1 (6.7)	5 (29.4)	1 (7.1)	7 (15.2)
TNM Stage				
III	3 (20.0)	6 (35.3)	4 (28.6)	13 (28.3)
IV	11 (73.3)	8 (47.1)	9 (64.3)	28 (60.9)
Time since initial diagnosis, months	34.3 (9.6, 97.1)	26.1 (5.2, 110.9)	26.0 (0.1, 88.8)	30.1 (0.1,110.9)
Prior lines of therapy				
0	1* (6.7)	0	0	1 (2.2)
1	2 (13.3)	7 (41.2)	5 (35.7)	14 (30.4)
2	4 (26.7)	3 (17.6)	1 (7.1)	8 (17.4)
≥ 3	8 (53.3)	7 (41.2)	8 (57.1)	23 (50.0)
PD-1 inhibitor exposure	1 (6.7)	1 (5.9)	0	2 (4.4)

Data are presented as median (range) or *n* (%). *ECOG* Eastern Cooperative Oncology Group. PD-L1 positivity: Tumor proportion score (TPS) ≥ 1%. PD-L1 negativity: TPS < 1%. * A patient diagnosed with thymic carcinoma experienced recurrence following surgical resection and adjuvant radiotherapy, and subsequently declined the standard chemotherapy regimen prior to enrollment in this study

Among the enrolled patients, cervical cancer was the most common histological type (21 patients; 45.7%). The median age of these cervical cancer patients was 53 years (range, 34–60 years), with 81% presenting with stage III–IV disease. Within this cohort, 90.5% had undergone radiotherapy, and 81% had received two or more prior lines of chemotherapy.

### Safety results

The safety profile of combining onatasertib with toripalimab was generally acceptable, with most toxicities being manageable. During the dose escalation phase, ten patients were evaluated across onatasertib combination dose levels of 15 mg (*n* = 3), 20 mg (*n* = 3), and 30 mg (*n* = 4), with no observed dose-limiting toxicities (DLTs) during the 21-day evaluation period.

As of October 20, 2023, all 46 patients had experienced at least one treatment-emergent adverse event (TEAE), as described in Table 2 and supplementary Table 1. The most common TEAEs included hyperglycemia (80.4%), increased blood lactate dehydrogenase (69.6%), rash (63.0%), proteinuria (63.0%), weight loss (58.7%), and lymphopenia (52.2%; Table 2). Most TEAEs were grade 1-2, with 9.1% being grade 3–4. The most common grade 3–4 AEs were lymphopenia (23.9%), rash (19.6%), hyperglycemia (10.9%), anemia (10.9%), and hypokalemia (10.9%; Table 2). Twenty-three patients experienced at least one serious AE, as detailed in supplementary Table 2. Within 30 days of the last dose, six patients recorded TEAEs leading to death: five deaths were reported disease progression, and one myocardial infarction was reported as treatment-related TEAE leading to death.

**Table 2:** Treatment-emergent adverse events in all included patients

	Onatasertib 15 mg + Toripalimab 240 mg (*n* = 15)		Onatasertib 20 mg + Toripalimab 240 mg (*n* = 17)		Onatasertib 30 mg + Toripalimab 240 mg (*n* = 14)		All enrolled patients (*n* = 46)	
	Any grade	Grade 3–4	Any grade	Grade 3–4	Any grade	Grade 3–4	Any grade	Grade 3–4
Total patients with event	15	12	17	12	14	12	46	36
Hyperglycemia	12 (80.0)	1 (6.7)	12 (70.6)	2 (11.8)	13 (92.9)	2 (14.3)	37 (80.4)	5 (10.9)
Blood LDH increased	10 (67.7)	0	11 (64.7)	0	11 (78.6)	0	32 (69.6)	0
Rash	11 (73.3)	3 (20.0)	11 (64.7)	6 (35.3)	7 (50.0)	0	29 (63.0)	9 (19.6)
Proteinuria	10 (67.7)	0	8 (47.1)	0	11 (78.6)	0	29 (63.0)	0
Weight loss	9 (60.0)	0	9 (52.9)	0	9 (64.3)	0	27 (58.7)	0
Lymphopenia	10 (67.7)	4 (26.7)	8 (47.1)	4 (23.5)	6 (42.9)	3 (21.4)	24 (52.2)	11 (23.9)
Hypokalemia	8 (53.3)	0	7 (41.2)	4 (23.5)	6 (42.9)	1 (7.1)	21 (45.7)	5 (10.9)
α-hydroxybutyrate dehydrogenase increased	7 (46.7)	0	7 (41.2)	0	7 (50.0)	0	21 (45.7)	0
WBC decreased	6 (40.0)	0	10 (58.8)	0	4 (28.6)	0	20 (43.5)	0
Anemia	8 (53.3)	3 (20.0)	7 (41.2)	1 (5.9)	5 (35.7)	1 (7.1)	20 (43.5)	5 (10.9)
Anorexia	9 (60.0)	0	4 (23.5)	0	6 (42.9)	0	19 (41.3)	0
Nausea	6 (40.0)	0	6 (35.3)	0	7 (50.0)	0	19 (41.3)	0
Malaise	8 (53.3)	0	5 (29.4)	0	6 (42.9)	0	19 (41.3)	0
Diarrhea	6 (40.0)	1 (6.7)	9 (52.9)	2 (11.8)	4 (28.6)	0	19 (41.3)	3 (6.5)
Hypophosphatemia	4 (26.7)	0	5 (29.4)	0	9 (64.3)	0	18 (39.1)	0
CPK increased	6 (40.0)	0	7 (41.2)	0	5 (35.7)	0	18 (39.1)	0
Lipase increased	7 (46.7)	1 (6.7)	7 (41.2)	0	2 (14.3)	1 (7.1)	16 (34.8)	2 (4.3)
Hypoalbuminemia	7 (46.7)	0	5 (29.4)	0	3 (21.4)	0	15 (32.6)	0
Hypertriglyceridemia	4 (26.7)	1 (6.7)	8 (47.1)	2 (11.8)	2 (14.3)	0	14 (30.4)	3 (6.5)
Vomiting	5 (33.3)	0	2 (11.8)	0	7 (50.0)	0	14 (30.4)	0
Creatinine increased	5 (33.3)	0	4 (23.5)	0	4 (28.6)	0	13 (28.3)	0
Hyponatremia	3 (20.0)	0	5 (29.4)	0	4 (28.6)	0	12 (26.1)	0
Pruritus	7 (46.7)	0	5 (29.4)	0	0	0	12 (26.1)	0
GGT increased	6 (40.0)	0	3 (17.6)	0	3 (21.4)	0	12 (26.1)	0
AST increased	7 (46.7)	0	2 (11.8)	0	2 (14.3)	0	11 (23.9)	0
Oral ulcer	4 (26.7)	0	3 (17.6)	0	4 (28.6)	2 (14.3)	11 (23.9)	2 (4.3)
Cholesterol high	5 (33.3)	0	6 (35.3)	0	0	0	11 (23.9)	0
Hypochloremia	4 (26.7)	0	4 (23.5)	0	3 (21.4)	0	11 (23.9)	0
Urinary tract infection	3 (20.0)	1 (6.7)	4 (23.5)	1 (5.9)	3 (21.4)	0	10 (21.7)	2 (4.3)
Hypothyroidism	6 (40.0)	0	2 (11.8)	0	2 (14.3)	0	10 (21.7)	0
Hypoproteinemia	4 (26.7)	0	3 (17.6)	0	3 (21.4)	0	10 (21.7)	0
Hypocalcemia	4 (26.7)	0	2 (11.8)	0	4 (28.6)	0	10 (21.7)	0
Mucositis oral	4 (26.7)	0	4 (23.5)	0	2 (14.3)	0	10 (21.7)	0
Platelet count decreased	3 (20.0)	0	2 (11.8)	0	4 (28.6)	2 (14.3)	9 (19.6)	2 (4.3)
Pneumonitis	4 (26.7)	3 (20.0)	1 (5.9)	0	0	0	5 (10.9)	3 (6.5)
Abdominal pain	2 (13.3)	0	0	0	2 (14.3)	2 (14.3)	4 (8.7)	2 (4.3)

Data are presented as *n* (%). Data are shown for adverse events that occurred in at least 20% of patients and grade 3–5 adverse events occurring in ≥2 patients. Events are ordered by overall frequency. *LDH* lactate dehydrogenase, *WBC* white blood cell, *CPK* creatine phosphokinase, *GGT* glutamyl transferase, *AST* aspartate transaminase

Thirty patients experienced at least one dose modification due to toxicity, primarily rash (23.9%) or hyperglycemia (8.7%; supplementary Table 3). The relative dose intensity decreased with increasing dose (supplementary Table 4), indicating that more dose modifications were required at higher dose levels. Thirteen patients required dose reduction at least once: 6 at the 20 mg dose, and 7 at the 30 mg dose. The mean duration of therapy with onatasertib for these 46 patients was 28.9 weeks, which varied notably among the different dose regimens (15 mg, 43.6 weeks; 20 mg, 27.2 weeks; and 30 mg, 15.2 weeks), details are presented in supplementary Table 4.

### Pharmacokinetics

The pharmacokinetic (PK) analysis set included 10 patients from the dose escalation phase: three each at the 15 mg and 20 mg dose levels, and four at the 30 mg dose level (one patient did not undergo PK sampling during the multiple-dose phase). Supplementary Fig. 1 and supplementary Table 5 present the single-dose and multiple-dose plasma concentration-time profiles and PK parameters of onatasertib when administered with toripalimab. Results indicated co-administration of toripalimab may not significantly affect the PK profiles of onatasertib.

### Efficacy

**Fig. 2:**
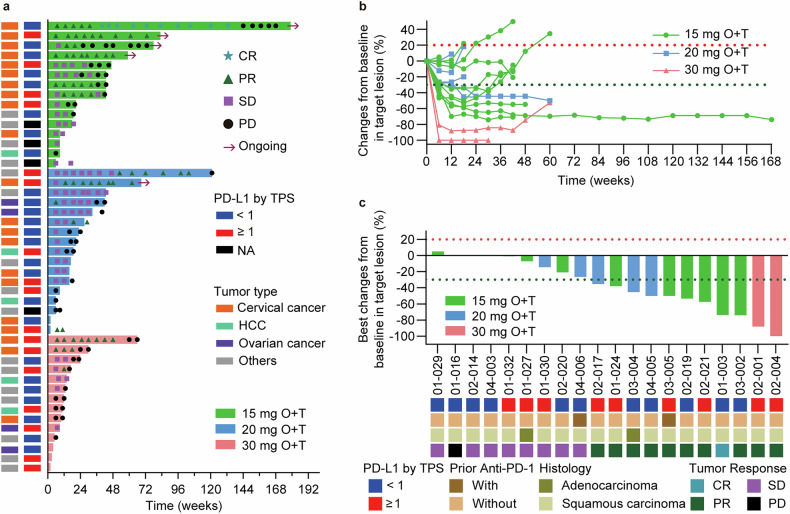
Tumor response to the combination of onatasertib and toripalimab. **a** A swimming plot illustrates treatment duration, time to best overall response, and time to progression by tumor type for all 46 patients. Each bar represents an individual patient. The dosing regimens include 15 mg O + T (onatasertib 15 mg QD + toripalimab 240 mg Q3W), 20 mg O + T (onatasertib 20 mg QD + toripalimab 240 mg Q3W), 30 mg O + T (onatasertib 30 mg QD + toripalimab 240 mg Q3W). HCC: Hepatocellular carcinoma. **b** A spider plot displays target lesion response over time for cervical cancer patients. Each line represents a single patient; lines are colored by different dose group as indicated. The horizontal dotted black line indicates a ≥ 30% decrease in the size of target lesion compared to baseline. **c** A waterfall plot displays the best response in target lesion among cervical cancer patients. Patients without evaluation was excluded from the waterfall plot. Each bar represents an individual patient. Dotted lines at −30% and 20% indicate boundaries for response and progression, respectively. Notably, the complete response (CR) patient 01-003, listed at the top of Fig. 2a and supplementary Fig. 4, received onatasertib and toripalimab for two years, then discontinued toripalimab. After nine months of onatasertib monotherapy, the patient experienced disease progression and was retreated with toripalimab, and the lesion disappeared after two cycles of combination therapy. The patient received retreatment with toripalimab and the lesion disappeared after two cycles of combination treatment. Five patients are on therapy for <6 weeks. Among them, one patient was assessed as a partial response (PR). The remaining four patients discontinued the study due to intolerance

Of the 46 patients enrolled, 11 achieved partial response (PR), and one achieved complete response (CR) according to RECIST version 1.1 (Fig. 2a; Table 3). Four patients discontinued the study prematurely due to intolerance, preventing them from completing scheduled efficacy evaluations. Three of these patients were in the 30 mg dose cohort, and one was in the 20 mg dose cohort. The objective response rate (ORR) among all dose cohort was 26.1%, with the highest rate observed in the 15 mg dose cohort (40.0%; 95% confidence intervals [CI]: 16.3–67.7), compared to 23.5% (95% CI: 6.8–49.9) in the 20 mg dose cohort and 14.3% (95% CI: 1.8–42.8) in the 30 mg dose cohort. The disease control rate (DCR) was 73.9%, with the highest rate reaching 93.3% in the 15 mg dose cohort. The median duration of response was 12.3 months (range, 4.1–NE months), and the median time to response was 1.5 months (range, 1.3–1.8 months).

**Table 3:** Best overall anti-tumor response

Overall response (All patients)	Onatasertib 15 mg + Toripalimab 240 mg (*n* = 15)	Onatasertib 20 mg + Toripalimab 240 mg (*n* = 17)	Onatasertib 30 mg + Toripalimab 240 mg (*n* = 14)	All enrolled patients (*n* = 46)
Complete response	1 (6.7)	0	0	1 (2.2)
Partial response	5 (33.3)	4 (23.5)	2 (14.3)	11 (23.9)
Stable disease	8 (53.3)	9 (52.9)	5 (35.7)	22 (47.8)
Progressive disease	1 (6.7)	3 (17.6)	4 (28.6)	8 (17.4)
Not evaluated	0	1 (5.9)	3 (21.4)	4 (8.7)
Objective response^†^ (95% CI)	40.0 (16.3,67.7)	23.5 (6.8,49.9)	14.3 (1.8,42.8)	26.1 (14.3,41.1)
Disease control^‡^ (95% CI)	93.3 (68.1,99.8)	76.5 (50.1, 93.2)	50 (23,77.0)	73.9 (58.9,85.7)
Progression-free survival, months Median (95% CI)	5.8 (3.3,9.6)	4.1 (3.1, NE)	3.1 (1.3,4.3)	4.3 (3.5,7.2)
Overall survival, months Median (95% CI)	17.7 (4.6, NE)	19.9 (8.3, NE)	10.2 (5.5,16.4)	15.7 (12.1,19.9)
**Overall response (Cervical cancer cohort)**	(*n* = 10)	(*n* = 8)	(*n* = 3)	(*n* = 21)
Complete response	1 (10.0)	0	0	1 (4.8)
Partial response	5 (50.0)	3 (37.5)	2 (66.7)	10 (47.6)
Stable disease	4 (40.0)	4 (50.0)	0	8 (38.1)
Progressive disease	0	0	1 (33.3)	1 (4.8)
Not evaluated	0	1 (12.5)	0	1 (4.8)
Objective response^†^ (95% CI)	60 (26.2,87.8)	37.5 (8.5,75.5)	66.7 (9.4,99.2)	52.4 (29.8,74.3)
Disease control^‡^ (95% CI)	100 (69.2,100)	87.5 (47.3,99.7)	66.7 (9.4,99.2)	90.5 (69.6,98.8)
Progression-free survival, months Median (95% CI)	7.8 (3.3, NE)	4.1 (3.7, NE)	5.5 (1.4, NE)	5.8 (4.1,13.7)
Overall survival, months Median (95% CI)	NE (4.6, NE)	NE (4.1, NE)	18.4 (15.1, NE)	18.4 (14.4, NE)

Data are presented as *n* (%), unless otherwise specified. ^†^The proportion of patients who achieved a complete response or partial response, according to RECIST version 1.1 criteria. ^‡^The proportion of patients who achieved a complete response (CR), partial response (PR), or stable disease (SD), according to RECIST version 1.1 criteria

**Fig. 3:**
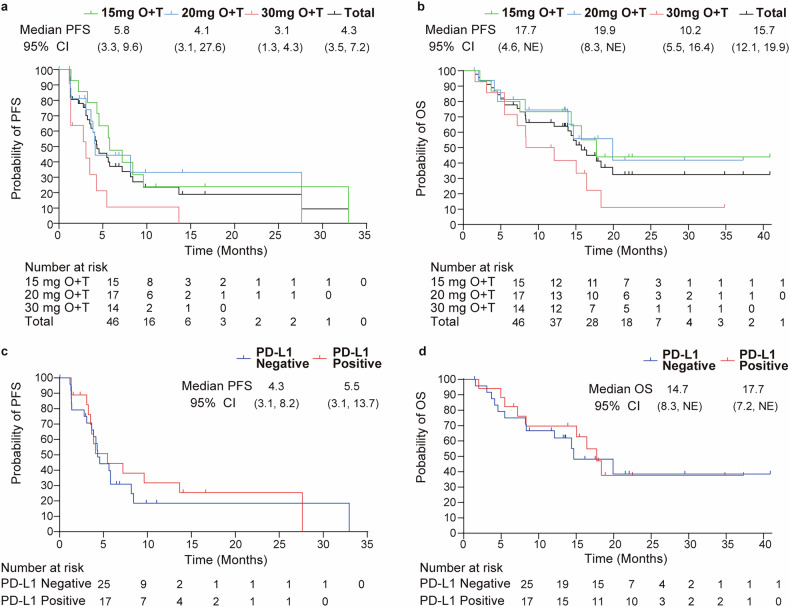
Kaplan-Meier estimation of survival outcomes as per RECIST version 1.1. Progression-free survival (PFS) and overall survival (OS) across three dose level cohorts (**a**, **b**), and PFS and OS stratified by PD-L1 status (**c**, **d**). The dosing regimens include 15 mg O + T (onatasertib 15 mg QD + toripalimab 240 mg Q3W), 20 mg O + T (onatasertib 20 mg QD + toripalimab 240 mg Q3W), 30 mg O + T (onatasertib 30 mg QD + toripalimab 240 mg Q3W)

As shown in Fig. 3a, b, the median progression-free survival (PFS) was 4.3 months (range, 3.5–7.2 months), and the median overall survival (OS) was 15.7 months (range, 12.1–19.9 months). Notably, patients receiving 15 mg dose exhibited the longest median PFS at 5.8 months (range, 3.3–9.6 months), while those receiving the 20 mg and 30 mg doses had median PFS values of 4.1 months (range, 3.1-27.6 months) and 3.1 months (range, 1.3–4.3 months), respectively. Prognostic outcomes varied across different tumor types, with cervical cancer being the most common in this study population and demonstrating relatively better outcomes (supplementary Figs. 2 and 3).

All 21 cervical cancer patients were included in the efficacy analysis. One patient achieved CR, 10 had PR and 8 had stable disease (SD), yielding a confirmed ORR of 52.4%, and a DCR of 90.5%. The median duration of response was 12.3 months (range, 4.2–NE months). There was no increasing trend in ORR when the combination dosage increasing, patients receiving the 15 mg dose demonstrated the best DCR of 100%. The median PFS and OS in cervical patients were 5.8 months and 18.4 months, respectively. The median PFS at the 15 mg dose was 7.8 months and the median OS was not reached (Fig. 2b).

PD-L1 expression in tumor samples was examined in 42 patients at baseline, and the correlation between the antitumor activity and PD-L1 expression was evaluated. In patients with PD-L1 expression (tumor proportion score [TPS] ≥ 1%), the median PFS was 5.5 months (95% CI: 3.1–13.7), the 24-month PFS rate was 23.6% (95% CI: 5.8–48.3), the median OS was 17.7 months (95% CI: 7.2–NE) and the 24-month OS rate was 37.6% (95% CI: 13.4–62.2). For PD-L1-negative patients, the median PFS was 4.3 months (95% CI: 3.1–8.2), the 24-month PFS rate was 19.4% (95% CI: 5.5–39.6), the 24-month OS rate was 38.5% (95% CI: 15.3–61.5), and the median OS was 14.7 months (95% CI: 8.3–NE; Fig. 3c, d). Notably, in the cervical cancer cohort, the ORR for patients with PD-L1-negative tumors were 41.7% (5/12) compared to 66.7% (6/9) for patients with PD-L1-positive tumors. Figure 3 presents the Kaplan–Meier curves for the PFS and OS for all populations and stratified by PD-L1 expression.

## Discussion

This study firstly demonstrated a manageable safety profile and encouraging antitumor activity of the mTORC1/2 inhibitor combined with CPI in patients with advanced solid tumors, particularly those with recurrent or metastatic cervical cancer. These findings expand our understanding of mTOR and PD-1 inhibition in oncology.

Considering the PK characteristics of both drugs and previous clinical studies involving combination therapy with toripalimab,^22,29^ compliance and convenience of patients, we selected a 21-day DLT observation period. No DLTs or unexpected safety events were observed across the explored dose levels. No new safety signal was observed in this study and the AE profile was consistent with previous studies of each agent used as monotherapy. Although 36 patients experienced at least one grade 3–4 AE, the total grade 3–4 AEs reported accounted for only 9.1% of all grades of AEs reported. The most common grade 3–4 AE was lymphopenia, which did not require clinical intervention. The second most common was rash, nine patients who experienced grade 3–4 rash were managed with oral dexamethasone or dexamethasone ointment at a dosage not exceeding 10 mg per day. In the phase 1 clinical trial of onatasertib, the most common TEAEs include hyperglycemia, fatigue, and rash.^22^ The incidence and severity of rash appeared higher than anticipated with either toripalimab or onatasertib alone,^22,24–27^ warranting further mechanistic investigation, including dermatology evaluations and molecular analysis of skin biopsies, to elucidate its underlying cause and relationship to therapeutic efficacy. Generally, toxicities related to the combination therapy were manageable, and the safety profile was acceptable.

Preliminary antitumor efficacy signals were observed, especially among patients with recurrent or metastatic cervical cancer, regardless of PD-L1 expression. Currently, there are no approved dual mTORC1/2 inhibitors for clinical oncological therapy. Temsirolimus showed limited efficacy in treating cervical cancer, with an ORR of 3% and a median PFS of 3.52 months.^30^ Pembrolizumab was the first PD-1 antibody globally approved for cervical cancer patients with positive PD-L1 expression based on KEYNOTE-028 and KEYNOTE-158 trials, achieving ORRs of 17% and 14.6% respectively.^31,32^ Notably, no responder was observed in patients with PD-L1 negative expression in KEYNOTE-158, highlighting the limitations of CPI monotherapy for such population.^32^ Two other studies of nivolumab (GY-002 and Checkmate-358) with mixed PD-L1 status of cervical cancer showed ORRs of 4% and 26.3%, respectively.^33,34^ In the present study, the ORR was 52.4%, and the DCR was 90.5% in cervical cancer patients, regardless of PD-L1 expression, suggesting remarkable potential in this combination strategy for patients with cervical cancer. The addition of an mTORC1/2 inhibitor onatasertib significantly improved the treatment response in patients without PD-L1 expression, providing a potential treatment option for these patients.

Several mTOR inhibitors have been approved for cancer treatment; however, concerns regarding their immunosuppressive properties have emerged.^35^ Studies have shown that mTOR inhibitors can impair T-cell activation and proliferation by decreasing the production of cytokines and growth factors essential for T-cell function and differentiation. They also hinder antigen uptake and modulate antigen presentation by dendritic cells.^36,37^ Recent research suggests that targeting mTOR pathway with agents capable of counteracting their immunosuppressive effects, such as checkpoint inhibitors, may enhance therapeutic outcomes.^21^ In fact, mTOR inhibitors can act as immune modulator to either suppress or activate immune responses, including modifying the TME, leading to increased tumor-infiltrating CD8 + T lymphocytes and PD-L1 expression within tumor tissues.^5,6,21^ Hirayama et al. reported that combination treatment significantly increases tumor-infiltrating lymphocyte (TIL) counts and the ratio of cytotoxic CD8 + T lymphocytes in a renal cell carcinoma mouse model.^38^ Vistusertib, a dual mTORC1/2 inhibitor combined with a checkpoint inhibitor, has also been shown to modulate the TME by reducing the occurrence of exhausted-phenotype TILs and increasing the frequency of activated Th1-polarized T lymphocytes in tumors.^21^ Previous studies have mechanistically demonstrated the direct, tumor-relevant immune-potentiating benefits of combining mTOR inhibition with immune checkpoint blockade. In addition, Xu et al. have demonstrated that the alterations in the PI3K-AKT pathway are prevalent in cervical cancer, which may partially explain the promising effectiveness of this combination therapy in cervical cancer.^13^ The mechanisms underlying the combination therapy in cervical cancer warrant further investigation.

In comparison to previously reported data for the drug used as monotherapy,^22^ the combination of toripalimab with onatasertib may not alter the PK characteristics of onatasertib. This finding suggests that the addition of toripalimab does not increase the risk of drug‒drug interactions.

Preliminary data from this study suggest that the onatasertib 15 mg combination cohort may have a more promising efficacy trend among the three dose groups. Safety data suggest that among all 46 patients, the 15 mg combination cohort experienced fewer TEAEs and TESAEs, along with higher relative dose intensity and longer treatment duration. The recommended phase 2 dose (RP2D), established through optimized dosage evaluation, comprises a daily administration of onatasertib at 15 mg in combination with 240 mg toripalimab Q3W.

There are several limitations of this study, first of all, this is a phase 1/2 non-randomized clinical trial, and randomized controlled trials involving more patients are required. Secondly, although we conducted biomarker tests for PD-L1 expression in a subset of patients, including test results from a larger patient population would be beneficial. Thirdly, given the potential synergistic effects of mTOR inhibitors through modulation of the TME, it is imperative to further explore the underlying mechanisms and associated biomarkers from both preclinical and clinical studies. Fourthly, this is the first exploratory prospective study of mTOR inhibitor and toripalimab, the inclusion of multiple advanced solid tumor types, each characterized by distinct natural histories and varying responses to systemic therapy, introduces complexity in evaluating treatment efficacy.

The toxicities associated with this combination therapy were manageable, and its safety profile was generally accepted. Preliminary evidence of active efficacy was observed in this study, particularly among cervical cancer patients, irrespective of PD-L1 expression status, suggesting potential clinical benefits for a broader patient population, including those with negative PD-L1 expression. These findings warrant further exploration into an optimal dosing regimen involving 15 mg onatasertib in combination with toripalimab for advanced cervical cancer patients. Ongoing efforts to enroll additional cervical cancer patients, including both CPI-naïve and CPI-treated individuals, as well as larger randomized controlled trials are proposed to further investigate PFS or OS benefits.

## Methods

### Study design and population

This TORCH-2 trial was a phase 1/2, open-label, dose escalation and dose expansion clinical study designed to assess the safety and tolerability of onatasertib in combination with toripalimab, as well as to evaluate its PK and efficacy in patients with advanced solid tumors. In dose escalation phase, a “3 + 3” design was employed. The enrollment of additional patients at varying doses was permitted to further determine the optimal dose of the combination therapy. During the dose expansion phase, eligible patients were sequentially allocated to each dose group (15 mg, 20 mg, or 30 mg) according to the order in which informed consent was obtained. The trial was conducted at six academic centers in China.

Patients aged 18 to 70 years with histologically confirmed advanced, relapsed, or refractory solid tumors were included in this study. Eligible patients had either received and progressed on or exhibited intolerance to, or refused at least one standard treatment regimen. Additional key eligibility criteria required the presence of at least one measurable lesion as defined by the Response Evaluation Criteria in Solid Tumors (RECIST) 1.1 or neuro-oncology criteria (RANO criteria). Furthermore, adequate bone marrow function, coagulation status, and organ function were also needed. Patients who had previously been treated with mTOR (TORC1 and/or TORC2) inhibitors and/or PI3K/AKT/mTOR inhibitors were excluded.

This study was reviewed and approved by the Institutional Review Board of West China Hospital, Sichuan University. Written informed consent was obtained from each patient prior to screening. This study was registered at ClinicalTrial.gov (NCT04337463).

### Procedures

Onatasertib was administered orally QD at doses of 15 mg, 20 mg, and 30 mg. Toripalimab was given intravenously at a fixed dose of 240 mg Q3W. Patients could continue treatment until progressive disease, intolerable AEs, or met any another protocol-specified discontinuation criterion. For patients receiving toripalimab for more than 2 years, a collaborative discussion involving both researchers and sponsor was conducted to assess the continuation of the treatment. Administration of onatasertib need to be discontinued or the dosage reduced to a lower level for safety concerns under the conditions listed in the supplementary Table 6.

Safety evaluations included assessments for AEs, vital signs, physical examination, laboratory tests (including routine blood tests, blood biochemistry analyses, routine urine tests, and coagulation examinations), 12-lead electrocardiogram assessment, as well as blood pregnancy testing for women of childbearing age during and after treatment. Detailed documentation regarding AEs included descriptions of symptoms associated with AEs along with their occurrence timeframes, severity levels, causes related to AEs, and corresponding management strategies. AEs were graded according to the National Cancer Institute Common Terminology Criteria for Adverse Events (NCI CTCAE) version 5.0.

Efficacy evaluations were conducted by investigators utilizing the RECIST1.1 or the RANO criteria. Baseline tumor assessments were performed within 28 days prior to the initiation of treatment. Subsequent tumor assessments were scheduled at 6-week intervals (±7 days) following the first administration, continuing for the first 12 months. After the first year, assessments were conducted every 12 weeks (±7 days) until the occurrence of radiologically confirmed disease progression, initiation of new antitumor treatment, death, or withdrawal of consent, whichever occurred first. Post-progression survival was monitored every 12 weeks (±14 days) until death or consent withdrawal. Patients were permitted to continue treatment beyond iUPD (immune unconfirmed progressive disease) if the investigator determined that the patient might still derive clinical benefit from the study treatment.

Blood samples for PK analysis of onatasertib and its primary metabolites (M1) were collected at predefined time points during the dose escalation phase. Sampling times included15 minutes (min) prior to the first dose of onatasertib and at 0.5 hours (h), 1 h, 1.5 h, 3 h, 5 h, 8 h, 24 h, and 48 h post dose. The PK sampling protocol on Cycle 1 Day 15 (C1D15) mirrored that of C1D1.

Formalin-fixed, paraffin-embedded tumor tissue samples obtained during screening was analyzed by immunohistochemical staining to assess tumor PD-L1 expression. TPS, defined as the percentage of PD-L1-stained tumor cells relative to the total number of viable tumor cells, was calculated and expressed as a percentage. A TPS ≥ 1% was classified as PD-L1 positive.

### End points and statistical analysis

The primary endpoint of this study was to assess the safety and tolerability of onatasertib in combination with toripalimab. Safety endpoints included AEs, laboratory abnormalities, vital signs, and physical examination findings. DLTs were evaluated within the first 21 days of onatasertib treatment initiation, and were attributed to either onatasertib or toripalimab based on investigators’ judgment. DLTs were defined as follows: non-hematological toxicity: any ≥grade 3 adverse events, excluding rash that improved to ≤grade 2 within 7 days; laboratory abnormalities or digestive reactions that resolved to ≤grade 2 within 3 days; grade 3 fatigue lasting ≤7 days; grade 3 infusion reactions lasting ≤6 h, and alopecia. Hematological toxicity: grade 4 neutropenia persisting >7 days; grade 3/4 febrile neutropenia; grade 4 thrombocytopenia, or hemorrhage associated with grade ≥3 thrombocytopenia.

Secondary endpoints included ORR, DCR, time to response, PFS, OS, and PK characteristics. The ORR, which represents the proportion of patients achieving CR or PR according to RECIST criteria, was reported along with exact 95% confidence intervals (CIs). The iRECIST criteria were also used as an exploratory method for tumor evaluation. The DCR, defined as the proportion of patients achieving CR, PR or SD, was also reported with exact 95% CIs. The Kaplan‒Meier method was used to estimate the median and CI for time-to-event endpoints, including duration of response, PFS, and OS. SAS (version 9.4) was utilized for statistical analysis. Descriptive statistics were used to summarize all study data. Continuous variables are presented as numbers, while categorical variables are presented as numbers and percentages.

## Supplementary information

Supplementary material

study protocol

## Data Availability

The study protocol and all of the data generated and analyzed during this study are included in our manuscript and Supplemental Materials.
