# Supplementary material for: Multicenter phase 1/2 study of onatasertib, a dual TORC1/2 inhibitor, combined with the PD-1 antibody toripalimab in advanced solid tumors
Source: Signal Transduct Target Ther. 2025 Jun 25;10:198. doi: 10.1038/s41392-025-02281-0 (PMC12187923; doi:10.1038/s41392-025-02281-0)
Supplement: Supplementary file 1 — Supplementary material [file 41392_2025_2281_MOESM1_ESM.docx]

Supplementary Materials for

**Multicenter Phase 1/2 Study of Onatasertib Combined With Toripalimab in Advanced Solid Tumors**

Pei Shu1,2#, Xiaoyu Li1,2#, Qi Zhou3, Guiling Li4, Keqiang Zhang5, Li Yuan3, Yixian Liu2, Qiu Li1, Yongsheng Wang1, Hui Xie6*, Li Zheng1,2*

Correspondence to: Li Zheng (zhengli@wchscu.cn) or Hui Xie(huixie@vip.163.com)

**This PDF file includes:**

Figures. S1-4

Tables S1 to S6

**
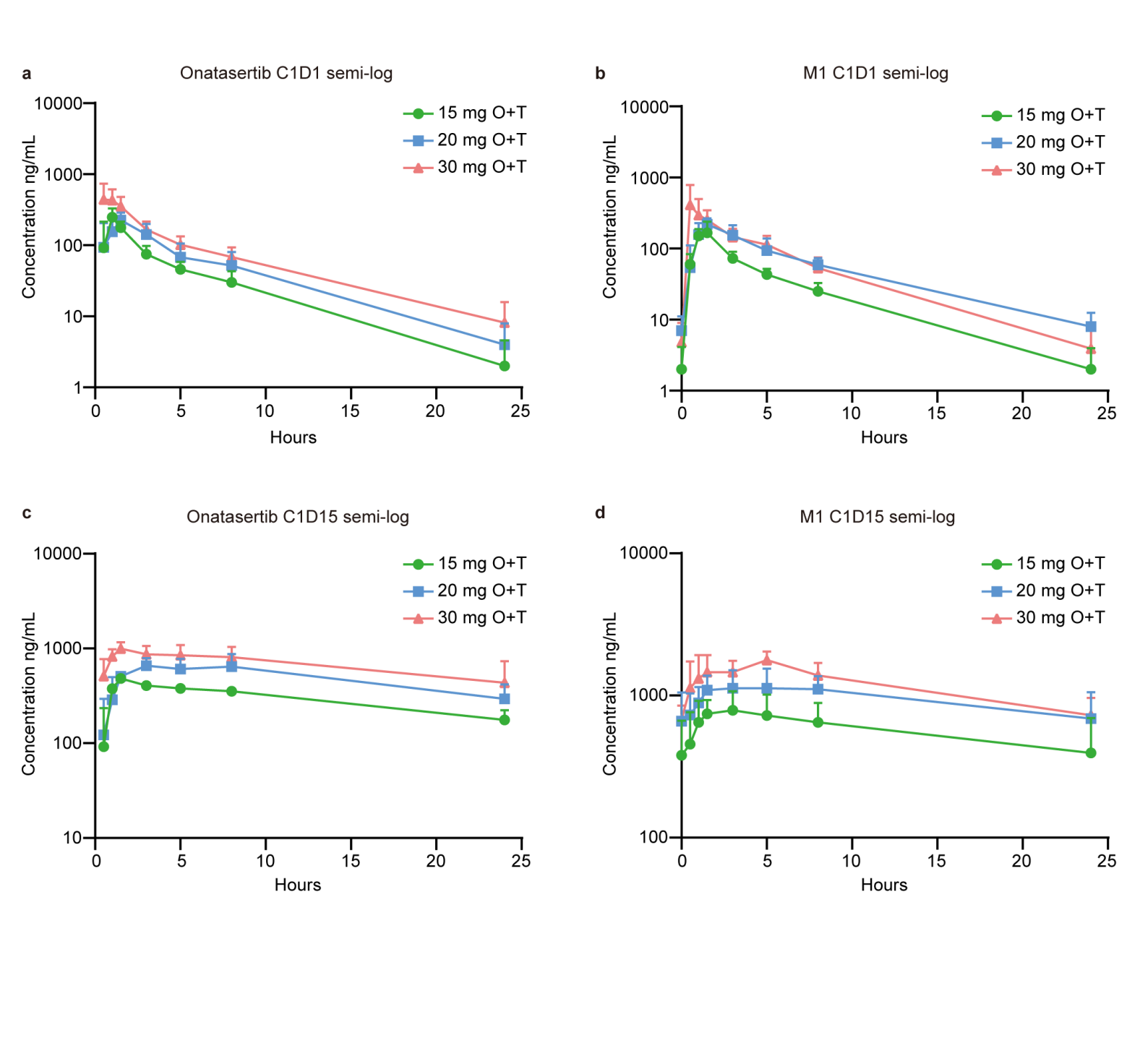
**

**supplementary Fig.1: Pharmacokinetics profiles**

Geometric mean plasma concentration-time profile of a. Onatasertib (15 mg QD, 20 mg QD and 30mg QD) in combination with toripalimab after a single

dose, b. M1 after single dose, c. Onatasertib (15 mg QD, 20 mg QD and 30mg QD) in combination with toripalimab after multiple doses and d. M1 after multiple doses. 15 mg O+T represents onatasertib 15 mg QD + toripalimab 240 mg Q3W, 20 mg O+T represents onatasertib 20 mg QD + toripalimab 240 mg Q3W, 30 mg O+T represents onatasertib 30 mg QD + toripalimab 240 mg Q3W.


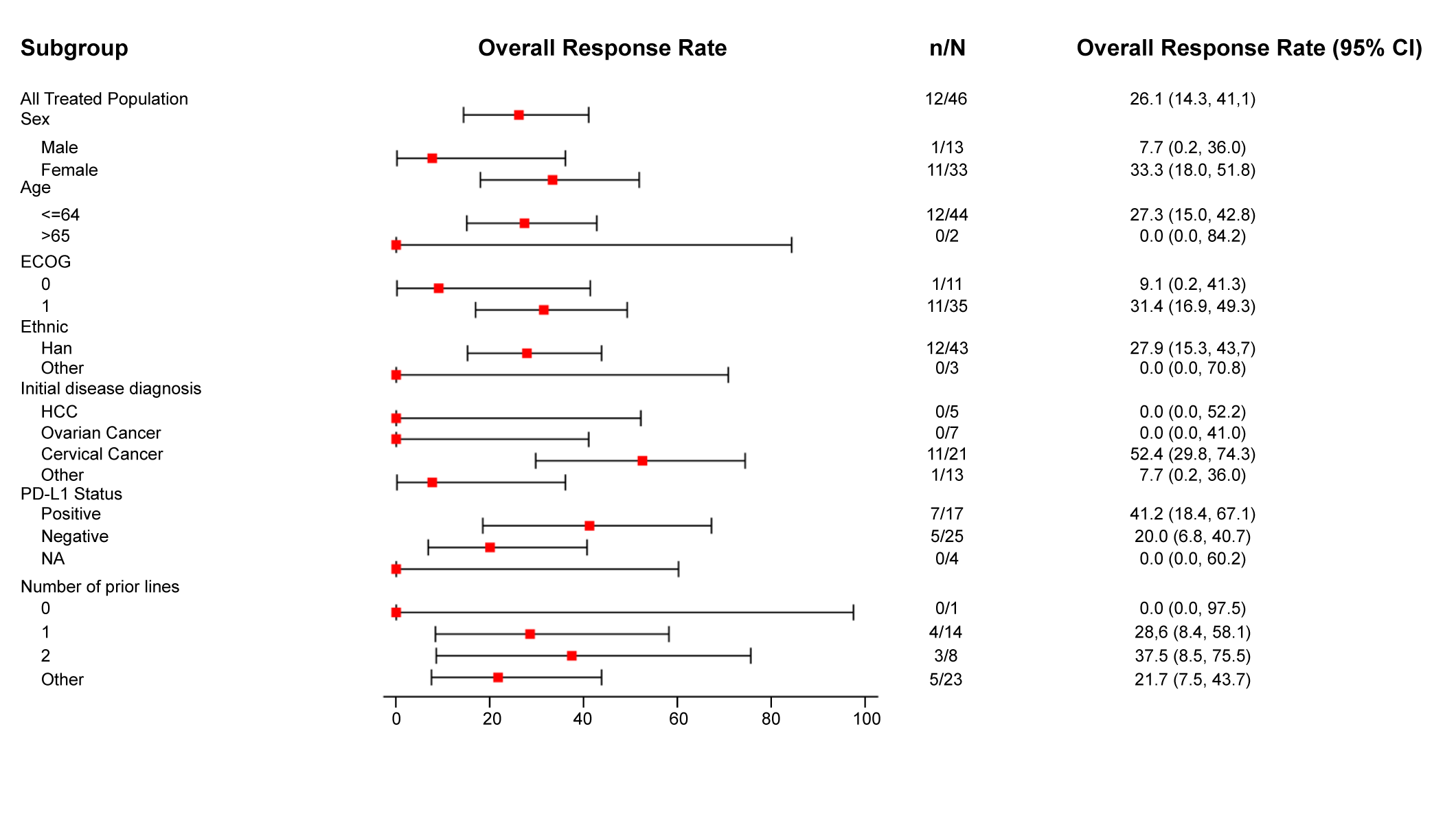


**supplementary Fig.2: Subgroup analysis for Overall Response Rate.** Given the pronounced numerical skewness, p-values were not calculable. Therefore, the differences in objective response rate among the groups are presented directly as observed data. CI, confidence interval, ECOG, Eastern Cooperative Oncology Group.


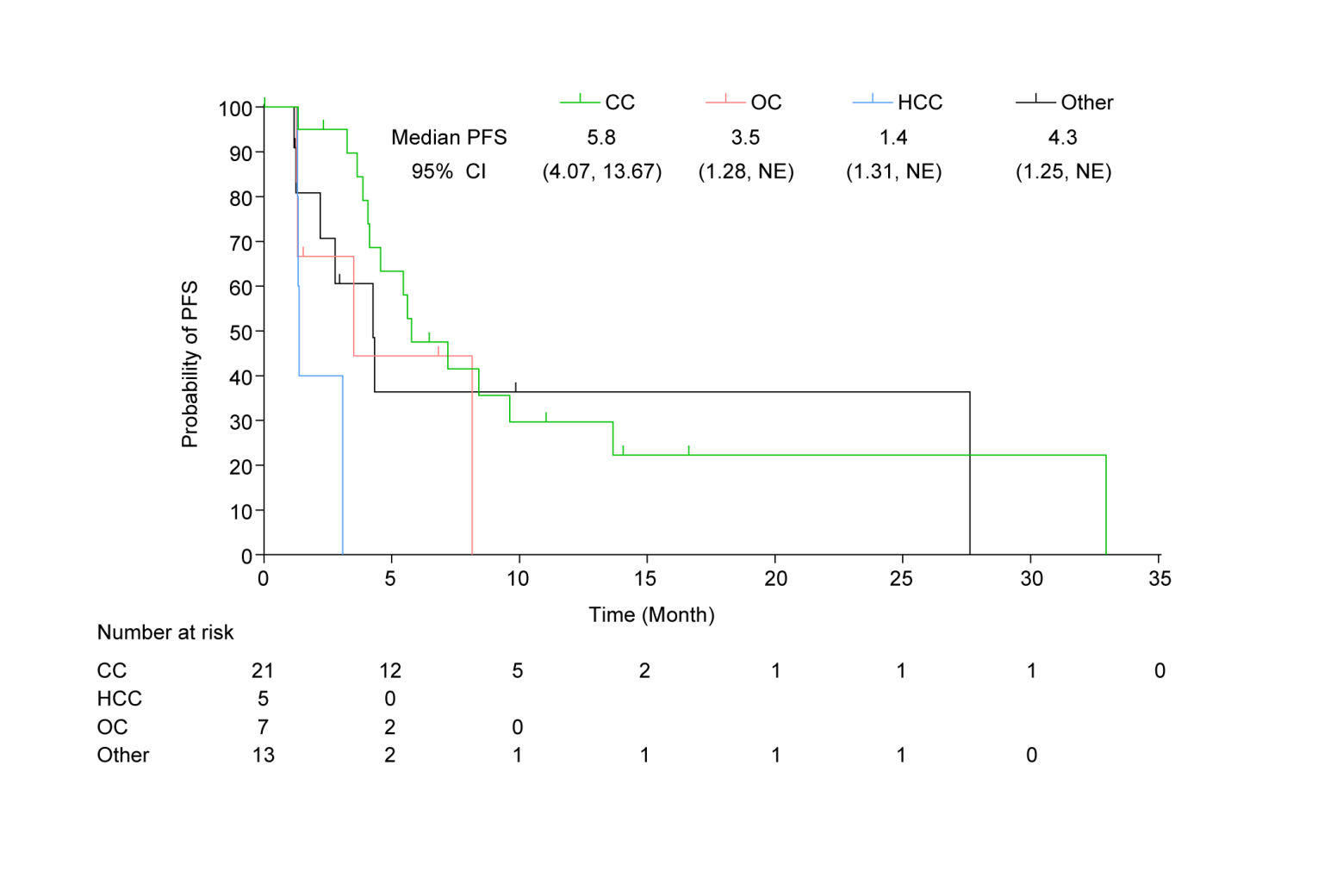


**supplementary Fig.3: Kaplan-Meier survival analyses were performed based on tumor type classification according to RECIST version 1.1 criteria.** The 'other' tumor category includes 2 nasopharyngeal carcinomas (NPC), 2 neuroendocrine tumors (NET), 2 thymic carcinomas, 1 glioblastoma multiforme (GBM), 1 renal cell carcinoma, 1 cholangiocarcinoma, 1 esophageal squamous cell carcinoma, 1 colorectal cancer, 1 lung cancer, and 1 breast cancer. CC, Cervical cancer; OC, ovarian cancer; HCC, hepatocellular carcinoma.

**
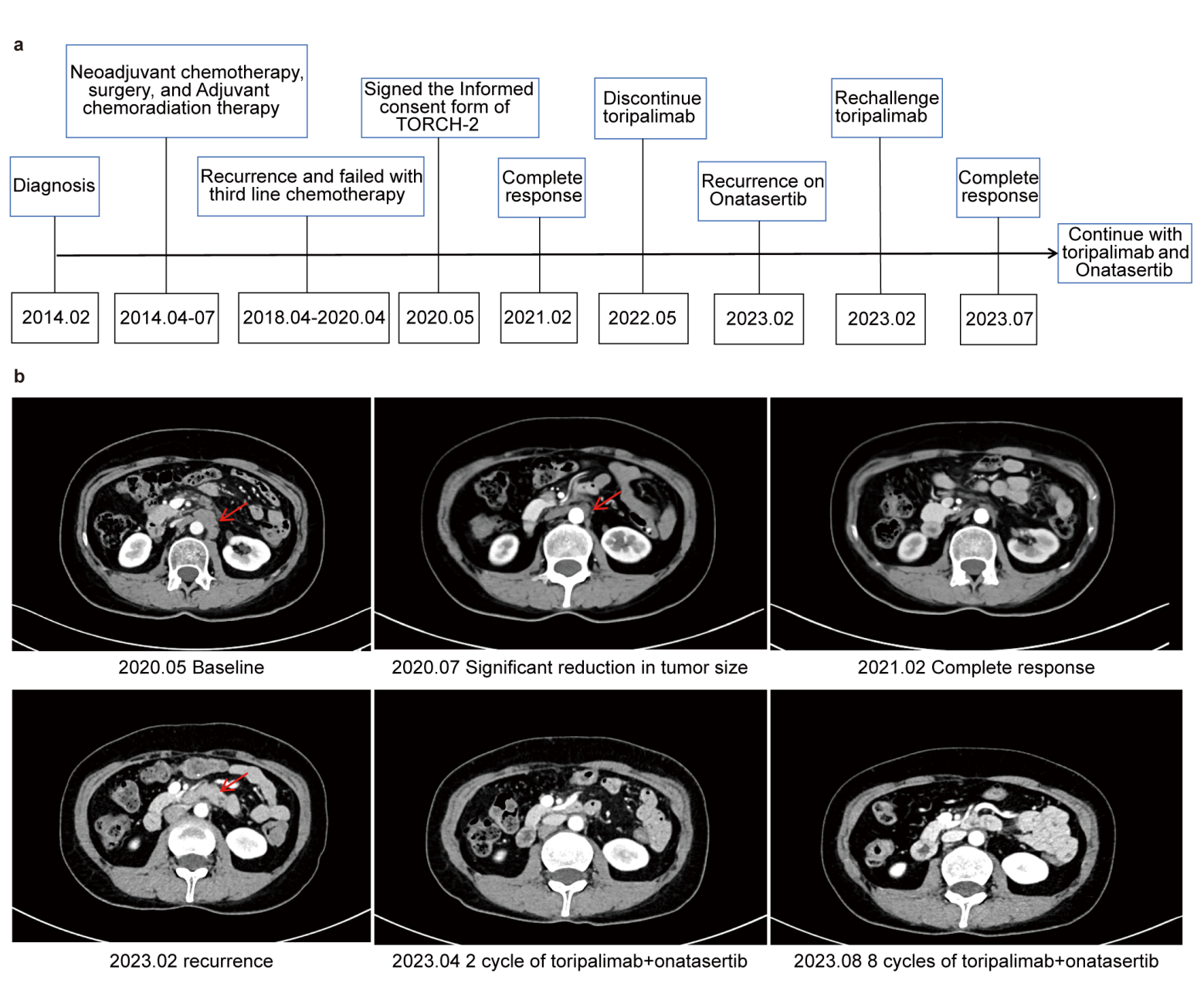
**

**supplementary Fig.4: Treatment history of patient 01-003.** a. Treatment timeline. b. Representative images. The patient 01-003 received onatasertib and toripalimab for two years, then discontinued toripalimab. After nine months of onatasertib monotherapy, the patient experienced disease progression and was retreated with toripalimab, and the lesion disappeared after two cycles of combination therapy. The patient received retreatment with toripalimab and the lesion was disappeared after two cycles of combination treatment.

**supplementary Table 1: Summary of adverse events**

| **Adverse event** | **Onatasertib 15 mg +**  **Toripalimab 240mg (n=15)** | **Onatasertib 20 mg +**  **Toripalimab 240mg (n=17)** | **Onatasertib 30 mg**  **+ Toripalimab 240mg (n=14)** | **All enrolled**  **patients (n=46)** |
| --- | --- | --- | --- | --- |
| Total patients with events | 15 (100.0) | 17 (100.0) | 14 (100.0) | 46(100.0) |
| Serious TEAE | 7 (46.7) | 9 (52.9) | 7 (50.0) | 23 (50.0) |
| Grade ≥3 TEAE | 12 (80.0) | 12 (70.6) | 12 (85.7) | 36 (78.3) |
| Leading to dose modification | 9 (60.0) | 11 (64.7) | 10 (71.4) | 30 (65.2) |
| Leading to dose reduction | 0 (0.0) | 6 (35.3) | 7 (50.0) | 13 (28.3) |
| Leading to treatment suspension | 9 (60.0) | 10 (58.8) | 8 (57.1) | 27 (58.7) |
| Leading to treatment termination | 1 (6.7) | 2 (11.8) | 2 (14.3) | 5 (10.9) |
| Death | 1 (6.7) | 3 (17.6) | 2 (14.3) | 6 (13.0) |

TEAE, treatment emergent adverse event.

**supplementary Table 2: Serious treatment-emergent adverse events**

|  | **Onatasertib 15 mg +**  **Toripalimab 240mg (n=15)** | **Onatasertib 20 mg +**  **Toripalimab 240mg (n=17)** | **Onatasertib 30 mg +**  **Toripalimab 240mg (n=14)** | **All enrolled**  **patients (n=46)** |
| --- | --- | --- | --- | --- |
| Total patients with event | 7 (46.7) | 9 (52.9) | 7 (50.0) | 23 (50.0) |
| Rash | 1 (6.7) | 4 (23.5) | 0 | 5 (10.9) |
| Disease progression | 1 (6.7) | 2 (11.8) | 1 (7.1) | 4 (8.7) |
| Hyperglycemia | 0 | 2 (11.8) | 2 (14.3) | 4 (8.7) |
| Fever | 0 | 1 (5.9) | 1 (7.1) | 2 (4.3) |
| Edema limbs | 0 | 1 (5.9) | 1 (7.1) | 2 (4.3) |
| Hypokalemia | 0 | 1 (5.9) | 1 (7.1) | 2 (4.3) |
| Diarrhea | 1 (6.7) | 1 (5.9) | 0 | 2 (4.3) |
| Fatigue | 1 (6.7) | 0 | 0 | 1 (2.2) |
| Electrolyte imbalance | 0 | 1 (5.9) | 0 | 1 (2.2) |
| Nutrition disorders | 0 | 1 (5.9) | 0 | 1 (2.2) |
| Erythema multiforme | 1 (6.7) | 0 | 0 | 1 (2.2) |
| Oral ulcer | 0 | 0 | 1 (7.1) | 1 (2.2) |
| Vomiting | 0 | 1 (5.9) | 0 | 1 (2.2) |
| Ileus | 0 | 0 | 1 (7.1) | 1 (2.2) |
| Urinary tract infection | 0 | 1 (5.9) | 0 | 1 (2.2) |
| Focal peritonitis | 0 | 0 | 1 (7.1) | 1 (2.2) |
| Shingles | 0 | 1 (5.9) | 0 | 1 (2.2) |
| Pleural effusion | 0 | 0 | 1 (7.1) | 1 (2.2) |
| Acute myocardial infarction | 0 | 0 | 1 (7.1) | 1 (2.2) |
| Immune mediated liver disease | 0 | 0 | 1 (7.1) | 1 (2.2) |
| Cholestatic jaundice | 0 | 1 (5.9) | 0 | 1 (2.2) |
| Immune-mediated kidney disease | 0 | 0 | 1 (7.1) | 1 (2.2) |
| Infection pneumonia | 1 (6.7) | 0 | 0 | 1 (2.2) |
| Premature ventricular contractions | 1 (6.7) | 0 | 0 | 1 (2.2) |
| Pericardial effusion | 1 (6.7) | 0 | 0 | 1 (2.2) |
| Epistaxis | 1 (6.7) | 0 | 0 | 1 (2.2) |
| Sensory motor disorders | 0 | 1 (5.9) | 0 | 1 (2.2) |
| Hepatocellular carcinoma | 0 | 1 (5.9) | 0 | 1 (2.2) |
| Deep vein thrombosis | 1 (6.7) | 0 | 0 | 1 (2.2) |

Data are presented as n (%).

**supplementary Table 3: Adverse events leading to treatment discontinuation**

|  | **Onatasertib 15 mg +**  **Toripalimab 240mg (n=15)** | **Onatasertib 20 mg +**  **Toripalimab 240mg (n=17)** | **Onatasertib 30 mg +**  **Toripalimab 240mg (n=14)** | **All enrolled patients (n=46)** |
| --- | --- | --- | --- | --- |
| Dose modification Rash  Hyperglycemia Fatigue  Diarrhea  Oral ulcer  Mucositis oral Hypokalemia  Neutrophil count decreased Platelet count decreased  COVID-19 infection  Fever  Anemia  Dizziness  Proteinuria  MRI anomaly  Lipase elevation  Urinary tract infection Focal peritonitis  Herpes zoster  Herpes virus infection Nasopharyngitis  Chills  Peripheral swelling Pericardial effusion Arrhythmology  Palpitations  Blood Hypercoagulability Myelosuppression  Conjunctival congestion Conjunctival hemorrhage Hematuria  Interstitial lung disease  Lethargy  Liver injury Flushing  Weight loss  Electrocardiogram ST-T change  White blood cell decreased Anorexia  Abdominal discomfort Abdominal pain  Ileus  Gingival pain Nausea  Vomiting  Erythema multiforme | 9 (60.0) | 11 (64.7) | 10 (71.4) | 30 (65.2) |
|  | 2 (13.3) | 7 (41.2) | 2 (14.3) | 11 (23.9) |
|  | 0 | 1 (5.9) | 3 (21.4) | 4 (8.7) |
|  | 1 (6.7) | 0 | 2 (14.3) | 3 (6.5) |
|  | 0 | 2 (11.8) | 0 | 2 (4.3) |
|  | 0 | 0 | 2 (14.3) | 2 (4.3) |
|  | 1 (6.7) | 0 | 1 (7.1) | 2 (4.3) |
|  | 0 | 1 (5.9) | 1 (7.1) | 2 (4.3) |
|  | 1 (6.7) | 0 | 1 (7.1) | 2 (4.3) |
|  | 1 (6.7) | 0 | 1 (7.1) | 2 (4.3) |
|  | 1 (6.7) | 1 (5.9) | 0 | 2 (4.3) |
|  | 0 | 1 (5.9) | 1 (7.1) | 2 (4.3) |
|  | 1 (6.7) | 0 | 1 (7.1) | 2 (4.3) |
|  | 0 | 1 (5.9) | 1 (7.1) | 2 (4.3) |
|  | 0 | 0 | 2 (4.3) | 2 (4.3) |
|  | 1 (6.7) | 0 | 0 | 1 (2.2) |
|  | 1 (6.7) | 0 | 0 | 1 (2.2) |
|  | 1 (6.7) | 1 (5.9) | 0 | 2 (4.3) |
|  | 0 | 0 | 1 (7.1) | 1 (2.2) |
|  | 0 | 1 (5.9) | 0 | 1 (2.2) |
|  | 1 (6.7) | 0 | 0 | 1 (2.2) |
|  | 1 (6.7) | 0 | 0 | 1 (2.2) |
|  | 0 | 0 | 1 (7.1) | 1 (2.2) |
|  | 0 | 0 | 1 (7.1) | 1 (2.2) |
|  | 1 (6.7) | 0 | 0 | 1 (2.2) |
|  | 0 | 0 | 1 (7.1) | 1 (2.2) |
|  | 0 | 0 | 1 (7.1) | 1 (2.2) |
|  | 0 | 0 | 1 (7.1) | 1 (2.2) |
|  | 0 | 0 | 1 (7.1) | 1 (2.2) |
|  | 0 | 1 (5.9) | 0 | 1 (2.2) |
|  | 1 (6.7) | 0 | 0 | 1 (2.2) |
|  | 0 | 0 | 1 (7.1) | 1 (2.2) |
|  | 1 (6.7) | 0 | 0 | 1 (2.2) |
|  | 1 (6.7) | 0 | 0 | 1 (2.2) |
|  | 0 | 0 | 1 (7.1) | 1 (2.2) |
|  | 0 | 1 (5.9) | 0 | 1 (2.2) |
|  | 0 | 1 (5.9) | 0 | 1 (2.2) |
|  | 0 | 0 | 1 (7.1) | 1 (2.2) |
|  | 1 (6.7) | 0 | 0 | 1 (2.2) |
|  | 0 | 1 (5.9) | 0 | 1 (2.2) |
|  | 1 (6.7) | 0 | 0 | 1 (2.2) |
|  | 0 | 0 | 1 (7.1) | 1 (2.2) |
|  | 0 | 0 | 1 (7.1) | 1 (2.2) |
|  | 0 | 0 | 1 (7.1) | 1 (2.2) |
|  | 0 | 0 | 1 (7.1) | 1 (2.2) |
|  | 0 | 0 | 1 (7.1) | 1 (2.2) |
|  | 1 (6.7) | 0 | 0 | 1 (2.2) |

Data presented as n (%). COVID-19, Corona Virus Disease

**supplementary Table 4: Treatment exposure**

|  | **Onatasertib 15 mg +**  **Toripalimab 240mg (n=15)** | **Onatasertib 20 mg +**  **Toripalimab 240mg (n=17)** | **Onatasertib 30 mg +**  **Toripalimab 240mg (n=14)** | **All enrolled patients (n=46)** |
| --- | --- | --- | --- | --- |
| Agent | Onatasertib Toripalimab | Onatasertib Toripalimab | Onatasertib Toripalimab | Onatasertib Toripalimab |
| Exposure Duration, weeks, Mean (SD) | 43.6 (44.5) NA | 27.2 (29.6) NA | 15.2 (16.6) NA | 28.9 (33.8) NA |
| Cycles of therapy Mean (SD) | 14.6 (14.8) 14.0 (14.6) | 9.1 (9.9) 8.5 (8.5) | 5.1 (5.5) 5.6 (5.4) | 9.6 (11.3) 9.4 (10.6) |
| Total dosage, mg | 4510.0 3136.0 | 2933.5 2047.1 | 2097.1 1337.1 | 3193.0 2186.1 |
| Mean (SD) | (4598.7) (2834.6) | (2921.8) (2042.0) | (2213.5) (1286.3) | (3465.2) (2234.4) |
| Dose intensity, mg/week Mean (SD) | 102.8 (6.1) 236.0 (13.7) | 117.5 (23.0) 240.0 (0) | 149.4 (31.7) 240.0 (0) | 122.4 (29.2) 239.0 (7.9) |
| Relative dose | 97.9 (5.8) 98.2 (5.7) | 83.9 (16.4) 100.0 (0) | 71.1 (15.1) 100.0 (0) | 84.6 (17.0) 99.4 (3.3) |
| intensity, % Mean (SD) |  |  |  |  |

SD, Standard Deviation.

**supplementary Table 5. PK parameters**

|  | Single dose phase | | | Multiple dose phase | | |
| --- | --- | --- | --- | --- | --- | --- |
|  | 15mg QD (N=3) | 20mg QD (N=3) | 30mg QD (N=4) | 15mg QD (N=3) | 20mg QD (N=3) | 30mg QD (N=3) |
| C_max (_mean, ng/mL) | 248 | 243 | 510 | 179 | 221 | 461 |
| T_1/2 (_mean, hours) | 4.1 | 4.1 | 4.76 | 4.4 | 5.1 | 4.23 |
| T_max_(median, hours) | 1 | 1.5 | 0.75 | 1.5 | 1.5 | 0.5 |

C_max_, maximum concentration; T_1/2_, terminal half-life; T_max,_ time to maximum concentration.

| **Supplementary Table 6. Recommended dose reduction guidelines for suspected onatasertib-associated adverse events** | | |
| --- | --- | --- |
| **Adverse events** | **Grade** | **Onatasertib dose reduction suggestion** |
| Thrombocytopenia | 3 | If bleeding occurs, reduce the dose of onatasertib by one level. |
|  | 4 | If it persists for more than 7 days, discontinue onatasertib until the severity grade is ≤ 2, then resume at a reduced dose. |
| Neutropenia | 3 | If fever occurs, reduce the dose of onatasertib by one level. |
|  | 4 | If the fever lasts for more than 7 days or is accompanied by other symptoms, discontinue onatasertib until the severity grade is ≤ 2, then resume at a reduced dose. |
| Elevated bilirubin levels | 3 | If laboratory values exceed 5 times the upper limit of normal (ULN), discontinue onatasertib until the severity grade is ≤ 2, then resume at a reduced dose. |
|  | 4 | Discontinue onatasertib until the severity grade is ≤ 2, then resume at a reduced dose. |
| Elevated aminotransferase | 3 | For values exceeding 10 times ULN, discontinue onatasertib until the severity grade is ≤ 2, then resume at a reduced dose. |
|  | 4 | Discontinue onatasertib until the severity grade is ≤ 2, then resume at a reduced dose. |
| Stomatitis/mucositis/vomiting/diarrhea | 3 | If optimal medical management cannot be achieved within 7 days, reduce the dose of onatasertib by one level |
|  | 4 | Discontinue onatasertib until the severity grade is ≤ 2, then resume at a reduced dose. |
| Hyperglycemia | 3 or 4 | Discontinue onatasertib if there is no response to best medical management within 7 days until the severity grade is ≤ 2, then resume at a reduced dose. |
| Rash | 3 | Discontinue onatasertib if there is no response to best medical management within 7 days until the severity grade is ≤ 2, then resume at a reduced dose. |
|  | 4 | Discontinue onatasertib. |
| Local acute pneumonia | 1 | Continue to use onatasertib with closely monitor. |
|  | 2 | Discontinue onatasertib until the severity grade is ≤ 1, then resume at a reduced dose. |
|  | 3 | Discontinue onatasertib. |
| fatigue | 3 | Discontinue onatasertib if there is no response to best medical management within 7 days until the severity grade is ≤ 2. |
| Other adverse events | 3 | Reduce onatasertib dose by one level. |
|  | 4 | Discontinue onatasertib. |
