# Supplementary material for: Multicenter phase 1/2 study of onatasertib, a dual TORC1/2 inhibitor, combined with the PD-1 antibody toripalimab in advanced solid tumors
Source: Signal Transduct Target Ther. 2025 Jun 25;10:198. doi: 10.1038/s41392-025-02281-0 (PMC12187923; doi:10.1038/s41392-025-02281-0)
Supplement: Supplementary file 2 — study protocol [file 41392_2025_2281_MOESM2_ESM.pdf]

This English version of the clinical research protocol is a translation of the original Chinese clinical research protocol (ATG-008-HX-001, Version: 2.1, Date: 15 Dec 2021). While every effort has been made to ensure translation accuracy, the Chinese language version shall always prevail in case of any discrepancies, conflicts, or ambiguities between the two versions.

Clinical Trial Protocol

**Name of Products:**	**ATG-008; Toripalimab**
**Protocol Number:**	**ATG-008-HX-001**
**Protocol Title:**	**An Open-Label, Dose Escalation and Expansion Study of a Dual TORC1/2 Inhibitor, ATG-008 Combined with PD- 1 Antibody, Toripalimab in Patients with Advanced Solid Tumors**
**Protocol Date:**	**3 Sep 2019, V1.0**<br>**28 Sep 2020, V2.0**<br>**15 Dec 2021, V2.1**
**IND Number:**	**NA**
**Sponsor Name/Address:**	**West China Hospital, Sichuan University**<br>**No. 37, Guoxue Lane,**<br>**Chengdu, Sichuan,**<br>**China**

CONFIDENTIALITY STATEMENT
This clinical trial protocol synopsis is confidential and the property of Sponsor and may not be used, disclosed or published without their consent.

Principal Investigator Signature Page

Principal Investigator Signature Date:

Principal Investigator's Printed Name： Li Zheng

Organization of clinical trial： West China Hospital

I hereby affirm through my signature that I will personally supervise the execution of this study at my affiliated research institution and ensure compliance with the following requirements: Study Protocol, Informed Consent Documentation, Institutional Review Board (IRB)/Ethics Committee (EC) Procedures, Declaration of Helsinki (DoH), ICH Guideline for Good Clinical Practice (ICH-GCP E6), Applicable local regulatory requirements governing clinical research conduct。

Contents

Principal Investigator Signature Page 2
Study Synopsis.. 6
Abbreviation 19
1. Background 21
1.2. Disease background 21
1.2.1. Hepatocellular carcinoma 21
1.2.2. Neuroendocrine tumor 22
1.2.3. Gynecological tumors in advanced stage or with metastasis (endometrial cancer, cervical cancer, ovarian cancer) 22
1.2.4. Glioblastoma multiforme...23
1.3. Research Drug Introduction 23
1.3.1. ATG-008..23
1.3.1.1. Rapamycin Target Protein...23
1.3.1.2. Clinical Efficacy Data of ATG-008..25
1.3.1.3. Safety Research data of ATG-008 (C-223)..27
1.3.1.4. Data of Pharmacokinetics of CC-223-ST-001 28
1.3.2. Toripalimab Injection 28
1.4. Research rationale..29
Figure 1. PD-1/PD-L1 anti-tumor signal pathway 30
Figure 2. In vivo antitumor activity of mTOR inhibitor combined with PD-1 monoclonal antibody.....30
Figure 3. In vivo anti-renal cell carcinoma activity of mTOR inhibitor combined with PD-1 monoclonal antibody 31
Potential Drug-drug Interactions: 32
1.4.1. Dose Selection rationale 33
1.4.3. Dose Selection Basis for Toripalimab 33
2. Objective and Endpoints Objective and Endpoints： 35
3. Study Design 38
3.1 Overall Design 38
3.1.1. Definition of DLT...36
3.2. Basic Rationale of Study Design..36
3.3. Study Duration 36
4. Study Population...37
4.1. Number of subjects 37
4.2. Inclusion criteria： 37
4.3. Exclusion criteria： 40
5. Registration and enrollment...43
5.2. The ID of the study subjects 43
5.3. Screening failure 43
6. Research procedure..44
6.1. Screening visit (28 days before the first dose administration) 44
6.2. Treatment Period (Combination Therapy with ATG-008 and Toripalimab) 45
6.3. End-of-Treatment Visit (0-7 days after the end of treatment)..47
6.4. Safety Follow-up (30 days ± 7 days after the last dose) 49
6.5. Information to be collected during safety follow-up includes:..49
6.6. Survival follow-up...49
6.7. Restrictions and considerations 49
7. Evaluation method...49
7.2. Medical history and treatment history 49
7.3. Combination of drugs...50
7.4. Physical examination and ECOG score..50
7.5. Vital signs...50
7.6. Height and weight...50
7.7. Evaluation of Child-Pugh score (HCC subjects only)..50
7.8. Evaluation of KPS Score (GBM Only) 50

7.9. Safety Assessment....50
7.10. Pharmacokinetic Assessment (Dose Escalation Phase Only)....52
7.11. Efficacy Assessment....52
7.11.2. Serum Anti-Drug Antibody (ADA) Assessment....53
7.12. Exploratory Evaluation....53
8. Termination Criteria....53
8.2. Premature Treatment Termination of Individual Subjects....53
8.3. Study Termination....54
9. Treatment Research....54
9.1. Administration Sequence....54
9.2. Drug Labeling....54
9.3. Drug Compliance....54
9.4. Overdose....55
9.5. ATG-008....55
9.5.2. Management of ATG-008-Related Adverse Reactions Hyperglycemia....55
9.5.3. Dose Adjustment of ATG-008....56
Table2 Recommended guidelines for reductions for suspected ATG-008-related toxic reactions....56
9.6.1. Preparation, Use, and Storage of Toripalimab Monoclonal Antibody....58
9.6.2. Management of Hypersensitivity Reactions....58
9.6.3. Management of Infusion Reactions....58
Table 3 Guidelines for the adjustment of treatment for infusion response of Toripalimab....59
9.6.4. Management of Immunotherapy-Related Adverse Events and Other Special Adverse Events....59
Table 4 General principles for the management of immunization-related adverse events*....61
9.7 of Concomitant Medications and Procedures....62
9.7.2. Permissible Concomitant Medications and Procedures....62
9.7.3. Prohibited Concomitant Medications and Procedures....63
9.7.4. Required Concomitant Medications and Procedures....64
9.8. Contraceptive Requirements....64
9.9. Inventory and Disposition of Investigational Medicinal Products....64
10. Adverse Events....65
Table 5 Classification of causality of adverse events....66
10.1.1. Events not meeting the definition of serious adverse events....66
10.2. Reporting of Serious Adverse Events....67
10.3. Follow-up of Adverse Events and Serious Adverse Events....67
10.5. Drug Overdose....68
10.6. Pregnant and Lactating Women....68
11. Statistical method....69
11.1.1. General Considerations in Statistics....69
11.1.2. Determination of Sample Size....69
11.1.3. Subject Distribution....69
11.1.4. Dose Adjustment....69
11.2. Analysis Population....69
11.3. Multiplicity....70
11.4. Data Analysis and Presentation....70
11.4.1. Demographic Characteristics....70
11.4.2. Baseline Characteristics and Medical History....70
11.4.3. PK Analysis....70
11.4.4. Efficacy Analysis....70
11.4.4.1. Exposure-Response Analysis....70
11.4.5. Safety Data Analysis....71
11.4.5.1. Adverse Events....71
11.4.5.2. Laboratory Examination Data....71
11.4.5.3. Vital Signs, Physical Examination, and ECOG Performance Status....71
11.4.5.4. Electrocardiogram (ECG) Assessment....72
11.4.5.5. Concomitant Medications....72
11.4.5.6. Deaths During the Study....72
11.4.6. Exploratory Analysis....72

11.4.7. Handling of Missing Data 72
11.5. Changes in Study Implementation or Prespecified Analysis 72
12. Regulatory, ethical and legal obligations 72
12.2. Institutional Review Board/Independent Ethics Committee 72
12.3. Regulatory Agency Approval 73
12.4. Protocol Compliance 73
12.5. Protocol Amendments 73
12.6. Informed Consent 73
12.7. Subject Confidentiality and Disclosure 73
12.8. Study Documentation, Recordkeeping, and Document Retention 73
12.9. Study Monitoring 74
12.10. Audits and Regulatory Agency Inspections 74
12.11. Information Disclosure 74
12.12. Study Termination 74
12.13. Reporting and Publication of Study Documents 74

Study Synopsis

Title of Study:

An Open-Label, Dose Escalation and Expansion Study of a Dual TORC1/2 Inhibitor, ATG-008 Combined with PD-1 Antibody, Toripalimab in Patients with Advanced Solid Tumors

Indications: Advanced Solid Tumors

Sample Size and Target Patient Population:

Dose Escalation Phase: Subjects with Advanced Solid Tumors (approximately 3-18 cases)
Dose Expansion Phase (approximately 50-150 cases):

- Subjects with Advanced Hepatocellular Carcinoma (HCC) (approximately 10-12 cases)
- Subjects with Advanced Neuroendocrine Tumor (NET) (approximately 10-12 cases)
- Subjects with Advanced Gynecological Tumors (endometrial, cervical and ovarian cancers) (approximately 10-12 cases)
- Subjects with Advanced Glioblastoma (GBM) (approximately 10-12 cases)
- Subjects with Advanced Solid Tumor (approximately 10-12 cases)

If the ORR of GBM cohort is about >10% or the ORR of other cohorts is about >20%, up to approximately 40 subjects will be enrolled in total in each corresponding cohort.

Objective and Endpoints:

Dose Escalation Phase	
**Primary Objectives**	**Primary Endpoints**
To evaluate the safety and tolerability of ATG-008 combined with toripalimab in subjects with advanced solid tumors	To establish MTD and RP2D of ATG-008 combined with toripalimab<br>To evaluate the safety and tolerability of ATG-008 combined with toripalimab
**Secondary Objectives**	**Secondary Endpoints**
To evaluate the pharmacokinetic characteristics of ATG-008 combined with Toripalimab	Pharmacokinetic parameters of ATG-008 for ATG-008 combined with toripalimab
To evaluate the efficacy of ATG-008 combined with toripalimab in subjects with advanced solid tumors	Evaluated by investigators according to RECIST 1.1 •<br>• ORR<br>• DOR<br>• DCR<br>PFS<br>OS
To evaluate the immunogenicity of toripalimab	Incidence rate of ADA
**Exploratory Objectives**	**Exploratory Endpoints**
To evaluate the efficacy of ATG-008 combined with toripalimab in subjects with advanced solid tumors according to iRECIST 1.1 criteria	Evaluated by investigators according to iRECIST: •<br>ORR, DOR, DCR, and PFS
To explore potential biomarkers in blood and tumor tissues	Levels of MSI, TMB and PD-L1 (tumor tissues), or sPD-L1(blood samples), etc.
**Dose Expansion Phase**	
**Primary Objectives**	**Primary Endpoints**
To evaluate the efficacy of ATG-008 combined with toripalimab in subjects with advanced solid tumors	ORR evaluated by investigators according to RECIST 1.1 or RANO criteria (GBM subjects only)
**Secondary Objective**	**Secondary Endpoints**
To further evaluate the efficiency of treatment with ATG-	Evaluated by investigators according to RECIST

008 combined with toripalimab in subjects with advanced solid tumors	1.1<br>• DOR<br>• DCR<br>• PFS<br>OS
To further evaluate the safety and tolerability of ATG-008 combined with toripalimab in subjects with advanced solid tumors	Safety and tolerability of ATG-008 combined with toripalimab
To further evaluate the immunogenicity of toripalimab	Incidence rate of ADA
**Exploratory Objectives**	**Exploratory Endpoints**
To evaluate the efficacy of ATG-008 combined with toripalimab in subjects with advanced solid tumors according to iRECIST and iRANO criteria (GBM subjects only)	Evaluated by investigators according to iRECIST and iRANO criteria (GBM subjects only)<br>• ORR、DOR、DCR and PFS
To explore the potential biomarkers in blood and tumor tissues	• Levels of MSI, TMB and PD-L1 (tumor tissues), or sPD-L1(blood samples),etc.<br>• Changes of CA125(ovarian cancers only)

Abbreviations: ADA = Antidrug Antibody; CA125= carbohydrate antigen 125; DCR = Disease Control Rate; DOR = Duration of Response; GBM = Glioblastoma; HCC = Hepatocellular Carcinoma; iRANO = Immunotherapy Response Assessment in Neuro-oncology; iRECIST = Evaluation Criteria for Immune Efficacy of Solid Tumors; MSI = Microsatellite Instability; MTD = Maximum Tolerated Dose; ORR = Overall Response Rate; OS = Overall Survival; PFS = Progression Free Survival; PK = Pharmacokinetics; RANO = Response Assessment In Neuro-Oncology; RECIST = Efficacy Evaluation Criteria For Solid Tumors; RP2D = Recommended Phase II Dose; Spd-L1 = Serum Free Programmed Death Ligand-1; TMB= Tumor Mutational Burden.

Study Design

This is an open-label study, including two phases: Dose Escalation Phase and Dose Expansion Phase. The safety and tolerability of ATG-008 combined with toripalimab, as well as DLT evaluation, maximum tolerated dose (MTD), and recommended phase II dose (RP2D) in advanced solid tumor subjects will be evaluated in the Dose Escalation Phase. Once RP2D is determined, Dose Expansion study will be conducted at the RP2D dose level in subjects with different types of solid tumors (including HCC, NET, advanced or metastatic gynecologic tumors, GBM, etc.) to further evaluate the anti-tumor efficacy and safety of ATG-008 combined with toripalimab.

ATG-008 will be administered orally once a day (QD), with a provisional maximum dose of 30 mg QD at present. Toripalimab will be administered intravenously as a fixed dose of 240 mg, once every 3 weeks (Q3W). The longest duration of toripalimab is not more than 2 years.

The investigational dosage as following.

Dose level	The combined treatment dose of ATG-008+Toripalimab	
	Toripalimab dose	ATG-008 dose
1	240 mg Q3W	15 mg QD
2	240 mg Q3W	20 mg QD
3	240 mg Q3W	30 mg QD

Q3W= Once every 3 Weeks; QD= Once a Day

When using the principles of the standard "3+3" design, for each dose level, the administration of the first dose of the study drug will be at least 72 hours apart between the first 2 subjects. At least 3 subjects will be enrolled for DLT evaluation in each dose level.

Safety Monitoring Committee (SMC) will be established by investigators (SMC will consist of investigators and medical representatives of drug provider) to evaluate safety and make decisions for dose escalation, including whether to modify dose escalation or determine RP2D based on the appearance of DLT.

After RP2D determination, subjects in the lower dose levels can be allowed to be escalated to the RP2D dose level.

Considering that the average dose in 47 subjects of NET cohort is about 20 mg according to the safety and tolerability data of solid tumors subjects in CC-223-ST-001-B phase I study, if 20 mg QD of ATG-008 dose level (ATG-008 combined with Toripalimab) shows good safety and tolerability at the end of the enrollment in Dose Escalation Phase, subjects in NET cohort will perform dose expansion at 20 mg QD of ATG-008 combined with Toripalimab in advance, while expansion dose of other cohorts will be determined after SMC discussion. In Dose Expansion Phase, subjects with different types of solid tumors will be enrolled at RP2D dose level, including the following 5 cohorts: advanced HCC, NET, advanced or metastatic gynecologic tumors (endometrial cancer, cervical cancer and ovarian cancer), GBM and advanced solid tumor subjects (approximately10-12 cases in each cohort), to further evaluate the efficacy, safety and tolerability of study drug at this dose level. If it is observed that ORR is about more than 10% in GBM cohort or about more than 20% in other cohorts, the number of subjects enrolled in each corresponding cohort will be increased up to approximately 40 cases.

Study population Inclusion criteria：

Subjects must meet all the following inclusion criteria to be eligible for this study:

1. Know and voluntarily sign informed consent.
2. 18-70 years old (including 18 and 70 years old), weight ≥45 Kg (Dose escalation only).
3. At least one measurable lesion according to RECIST 1.1 or RANO criteria; lesions not recommended for target within the radiation field (except for GBM).
4. ECOG performance status score 0 or 1.
5. Clinical chemistry test results must meet the following:
    a) Aspartate aminotransferase (AST) and alanine aminotransferase (ALT) ≤2.5 ×

normal upper limit (ULN)

b) Total bilirubin ≤1.5 × ULN
c) Serum albumin >29 g/L
d) Creatinine ≤1.5 × ULN or 24-hour serum creatinine clearance ≥50 mL/min
e) Lipase and amylase ≤2 × ULN.

6. Adequate bone marrow function and meet the following criteria:
   a) Absolute neutrophil count (ANC) ≥1.5 × 10^9 cells/L
   b) Platelet count ≥75 × 10^9 cells/L
   c) Hemoglobin ≥90 g/L.
7. Coagulation function: International Normalized Ratio (INR) ≤ 2.0, Prothrombin Time (PT) ≤ 1.5 × ULN.
8. The hepatitis B virus/hepatitis C virus (HBV/HCV) test must meet the following criteria:
   a. For non-HCC subjects: Subjects with positive hepatitis B surface antigens (HBsAg) or HCV antibody, must further pass quantitative detection of HBV-DNA (no more than 2500 copies/mL or 500 IU/mL) and HCV-RNA (not exceed the lower limit of detection), and will be enrolled after ruling out the active hepatitis B or hepatitis C infection which need to receive treatment. Hepatitis B virus carriers, hepatitis B stable subjects after drug treatment (DNA quantitative detection should not be higher than 2500 copies/mL or 500 IU/mL) and cured hepatitis C subjects can be included in the study.
   b. For HCC subjects: Subjects with HBsAg positive and/or hepatitis B core antibody positive, meeting the following conditions can be included: HBV-DNA must < 2000 IU/mL or 10^4 copies/mL when enrollment, if higher than this, subjects must receive antiviral treatment at first to let HBV-DNA drop to the normal range for at least 2 weeks, and must continue to receive antiviral treatment during the study treatment. For subjects who have received antiviral therapy for hepatitis B in the past and have normal range of HBV-DNA when enrollment, antiviral therapy must be received throughout the study treatment. HCV-RNA positive subjects with normal liver function may also be enrolled in this study but must receive approved standard anti-HCV therapy.
9. Except for hearing loss and alopecia, all toxicity caused by previous anti-tumor therapy must have recovered to ≤ Grade 1 (according to NCI-CTCAE version 5.0).
10. Life expectancy is longer than 3 months.
11. Fertile men and women of childbearing age must agree to use effective contraceptives from they sign the informed consent to180 days after the last dose of study drug. Women of childbearing age include premenopausal women and women within 2 years after menopause. Women of childbearing age must have a negative blood pregnancy test at screening.

Inclusion criteria should also be met in Dose Escalation Phase：

1. Subjects are histopathologically confirmed with advanced, relapsed or refractory solid tumors (including but not limited to breast cancer, neuroendocrine cancer, lung cancer, cholangiocarcinoma, gastroesophageal junction adenocarcinoma and HCC). Subjects with HCC will meet the inclusion/exclusion criteria of the Dose Expansion Phase. No standard treatment available, intolerant or reject to standard treatment.

Inclusion criteria should also be met in Dose Expansion Phase：

1. Meets all the criteria for any of the following cohort

   <u>Advanced HCC Cohort:</u>

   a. Pathologically or clinically confirmed HCC, following Guidelines for Diagnosis and

Treatment of Primary Liver Cancer in China (2018 Edition)

b. Unresectable Stage B (intermediate stage) or Stage C (advanced stage) HCC according to Barcelona Clinic Liver Cancer Staging (BCLC). Subjects with stage B must have progressed after surgery /local treatment, or be unsuitable for surgery/local treatment.
c. Previously received at least one type of systemic therapy for HCC (including sorafenib, oxaliplatin-based chemotherapy, lenvatinib, or regorafenib, etc.).
d. Child-Pugh A or Child-Pugh B7 without encephalopathy

Advanced NET Cohort:

a. Histologically confirmed, unresectable advanced local or metastatic non-functional neuroendocrine neoplasms, including well-differentiated neuroendocrine neoplasms and poorly differentiated neuroendocrine carcinomas;
b. Subjects with poorly differentiated neuroendocrine carcinoma must have previously received platinum-based chemotherapy. Subjects with well-differentiated neuroendocrine tumors must have received at least one systemic treatment, including somatostatin analogues, antiangiogenic agents, chemotherapy, etc.

Advanced GBM Cohort:

a. Histologically confirmed GBM (WHO Grade IV);
b. Radiologically confirmed recurrence or progression after prior radiotherapy and temozolomide treatment;
c. Karnofsky Functional Status score (KPS) ≥70;
d. Prior to screening, antiepileptic drug therapy should be stable for at least 4 weeks or more.

Advanced or Metastatic Gynecologic Tumors Cohort:

a. Histologically confirmed, recurrent or metastatic gynecological tumors, any of the following:
   i. Ovarian cancer is epithelial ovarian, fallopian tube or peritoneal cancer, includes high-grade serous, endometrioid and clear cell carcinoma;
   ii. Cervical cancer is advanced, unresectable, unsuitable for radiotherapy and/or metastatic cervical cancer (squamous cell carcinoma, adenocarcinoma, adenosquamous carcinoma);
   iii. Advanced/recurrent endometrioidadenocarcinoma;
b. Progression or recurrence after previous chemotherapy containing platinum and/or taxol.

Advanced solid tumor cohort:

a. Subjects with histologically confirmed advanced, recurrent or refractory solid tumor that cannot be resected (recommended but not limited to soft-tissue sarcoma, papillary thyroid carcinoma [Thyroid Stimulating Hormone (TSH) level <0.5 mU/L], and cholangiocarcinoma).

2. No standard treatment available, intolerant or reject to standard treatment

Exclusion criteria：

Subjects meeting any of the following criteria are not allowed to be enrolled in the study：

1. Have a history of hepatic encephalopathy.
2. Have a history of organ transplantation (eg., liver transplantation).
3. Stable period determined by imaging <3 months, or symptomatic brain metastases (not applicable for GBM)

4. Have a thyroid disorder with a clinically significant thyroid dysfunction judged by the investigator (not applicable for thyroid cancer is in Dose Expansion Phase).
5. Active or history of upper gastrointestinal bleeding, ulcers, or esophageal varices with bleeding within 6 months.
6. Subjects with a history of human immunodeficiency virus (HIV) infection and/or acquired immunodeficiency syndrome.
7. Major surgery has been performed within 4 weeks prior to the first dose or is expected to be performed during the study period.
8. Have received systemic chemotherapy, radiotherapy, traditional Chinese medicine with anti-tumor activity, or local treatment for tumor (including but not limited to radiofrequency ablation, hepatic arterial chemoembolization, and high-intensity focused ultrasound) within 4 weeks prior to the first dose. Immunotherapy (including anti-PD-1, anti-PD-L1, cytotoxic T cell-associated protein 4 [CTLA-4] or CAR-T cell therapy, etc.) within 6 weeks prior to the first dose; Prior immunotherapy with less than 2 full cycles and progression or clinical judgment as hyperprogression of immunotherapy.
9. Received live attenuated vaccine within 4 weeks prior to the first study dose (Subjects should not receive live attenuated vaccine at the time of study drug administration and up to 28 days after the last dose if enrolled).
10. Poorly controlled pleural or pericardial effusion (with clinical symptoms, fluctuating fluid or requiring repeated drainage, oral diuretics, etc.) at Screening. Ascites can be detected during the physical examination at Screening, or clinical symptoms caused by ascites, or require special treatment, such as repeated drainage, intraperitoneal drug perfusion, etc. (But subjects with ascites could be considered applicable for enrollment if their ascites can only be found through image examination).
11. Other primary malignancies occurred within 5 years prior to the first study dose except for locally curable malignancies (eg., basal or squamous cell skin cancer, superficial bladder cancer, prostate, cervical or breast carcinoma in situ, etc.).
12. Suffering from active or previously had autoimmune diseases with recurring potential (eg., systemic lupus erythematosus, rheumatoid arthritis, inflammatory bowel disease, autoimmune thyroid disease, vasculitis, psoriasis disease, etc.), or at risk of such diseases.
13. Immunosuppressive drugs are systemically used currently or within 14 days prior to the first dose. Except following:
    a. Intranasal, inhaled, topical steroids, or topical steroid injections (such as intra-articular injections)
    b. Systemic corticosteroid treatment not exceeding 10 mg/day prednisone or equivalent physiological dose
    c. Steroids as a preventive medication for allergic reactions (such as pretreatment before CT scan).
14. Suffer a persistent or active infection, a history of pneumonia due to immunotherapy, or currently have Grade 2 above pneumonia, or a history of active pulmonary tuberculosis infection within 1 year prior to the first dose (Subject who has a history of active pulmonary tuberculosis infection more than 1 year ago, and currently has no evidence of active tuberculosis judged by investigator could be applicable for enrollment).
15. Have clinically significant cardiovascular diseases, such as Class II or above cardiac dysfunction (NYHA Functional Classification), ischemic heart disease (such as myocardial infarction or unstable angina pectoris), clinically significant supraventricular or ventricular arrhythmias, poorly controlled hypertension (systolic blood pressure> 150 mmHg and/or diastolic blood pressure> 100 mmHg), or echocardiogram showing

ejection fraction <50%, or QT corrected interval by using the heart rate (QTc) > 450 msec (male), 470 msec (female).

16. Any serious gastrointestinal disease that may affect the absorption of the study drug.
17. Previously received received mTOR (TORC1 and/or TORC2) inhibitors and/or PI3K/AKT/mTOR inhibitors.
18. Using/eating drugs or food known to have strong CYP3A4 inhibitory effects within 2 weeks prior to screening, including but not limited to atazanavir, clarithromycin, indinavir, itraconazole, ketoconazole, nefazodone, nelfinavir, ritonavir, saquinavir, telithromycin, vinegar oleandomycin, voriconazole, grapefruit or grapefruit juice.
19. Receiving drugs known to have strong CYP3A4 induction within 2 weeks prior to screening, including but not limited to carbamazepine, phenobarbital, phenytoin, rifabutin, rifampicin, and hyperforin perforatum.
20. Receiving drugs as CYP3A4 substrates (with narrow therapeutic index) within 2 weeks prior to screening, including but not limited to dihydroergodemine, ergodemine, pimozide, astimidazole, cisapride, and terfenadine.
21. Have a history of severe allergic reactions to chimeric, human or humanized antibodies, or fusion proteins; Known to have hypersensitivity reactions to any components produced from CHO cells, or a Toripalimab monoclonal antibody (such as citric acid monohydrate, sodium citrate dihydrate, mannitol, polysorbate).
22. Investigator considers that the complications or other situations of the subject may affect compliance with the protocol or is not suitable for participation in this study.

Exclusion criteria should also be met in Dose Escalation Phase as following:

1. Subjects with diabetes or glycated hemoglobin (HbA1c)> 7%.

Exclusion criteria should also be met in Dose Expansion Phase as following:

1. Poorly controlled diabetes (HbA1c >7%)
2. For HCC subjects:
    a. Histopathological diagnosis is fibrous lamellar HCC, sarcomatoid HCC, bile duct cell carcinoma, or mixed liver cancer;
    b. Have received molecular targeted therapy, such as sorafenib, orlenvatinib within 4 weeks or 5 half-lives prior to the first dose;
    c. Have received palliative surgery in the liver area, within 4 weeks prior to the first dose.
    d. Imaging findings of major portal vein cancer metastasis
3. For GBM subjects: those who have received carmustine wafer implantation or intracranial radioactive implantation

Study Duration

All subjects will continue to receive ATG-008 in combination with Toripalimab, during which the duration of Toripalimab treatment is usually no more than 2 years, until radiologically confirmed disease progression, intolerant toxicity, subject’s or investigator’s decision to discontinue medication, subject lost to follow-up, or death, whichever occurs first.

End of study is defined as 12 months after enrollment of the last subject in the study, withdrawal of consent, death, or loss to follow-up (whichever occurs first). In addition, the drug provider may decide to discontinue the study at anytime for reasons including, but not limited to safety.

After the subject's disease progression or the end of this study, if the subject needs to continue treatment, on the basis of the subject's will, the investigator can continue to administer the drug according to the subject's status, and with the consent of the drug provider, the subject can continue to provide ATG 008 and/or toripalimab (the longest time is usually not more than 2 years), and continue to collect the safety data of active reporting. ATG-008 and/or toripalimab maybe available in separate extension studies or other formats at the discretion of the drug provider.

Efficacy Assessments:

Efficacy assessments will be done by investigators based on the Response Evaluation Criteria in Solid Tumors (RECIST1.1) or the Response Assessment in Neuro-oncology (RANO [GBM only]) criteria. At Screening, all subjects have to undergo computed tomography (CT) or magnetic resonance imaging (MRI) scans of the chest, abdomen and pelvis, and skull MRI, according to RECIST1.1 or RANO criteria (GBM only). Subjects with bone lesions should undergo a confirmatory bone scan at screening. Baseline assessment of the tumor should be conducted not more than 28 days prior to administration and as close as possible to the commencement of administration or prior to administration, ideally. The investigator will also conduct exploratory efficacy evaluations according to the Evaluation Criteria for Immune Efficacy of Solid Tumors (iRECIST) for solid tumor and Immunotherapy Response Assessment in Neuro-oncology (iRANO) criteria for GBM subjects.

Tumor assessment will be performed 6 weeks after the first administration as the first imaging examination, will be performed once every 6 weeks (±7 days) in the first 12 months of treatment, and performed once every 12 weeks (±7 days) after 12 months of treatment, until radiologically confirmed disease progression, initiation of new anti-tumor treatment, death, or withdrawal of consent (whichever occurs first).

Information on all anti-tumor treatments used after the last dose of the study drug will be collected until death or withdrawal of consent. Disease progression status and date of disease progression should be recorded. Following disease progression, survival will be followed up once every 12 weeks (±14 days), until death or withdrawal of consent.

Serum anti-drug antibody (ADA) tests will be collected within 60 minutes prior to eachToripalimab administration in the first 6 cycles of the study treatment and thereafter will be collected once every 2 cycles (day 1 of the treatment cycle) until the subjects complete the End of Treatment visit; Sampling will also be required at the End of Treatment and Safety Follow-up visit, to evaluate the immunological effect of ATG-008 combined with Toripalimab.

Pharmacokinetic Assessments (only in Dose Escalation Phase)

In Dose Escalation Phase, each subject will be collected PK blood sample of ATG-008 at the following time points to evaluate the PK characteristics of ATG-008 and its metabolites in the subject after combined treatment.

PK sampling time point: 15 min prior to the first dose of ATG-008, and 0.5h±5min, 1h±10min, 1.5h±10min, 3h±10min, 5h±15min, 8h±30min, 24h±60min, and 48h±60min after the first dose of ATG-008. 15min pre-dose of ATG-008, and 0.5h±5min, 1h±10min, 1.5h±10min, 3h±10min, 5h±15min, 8h±30min, and 24h±60min post-dose of ATG-008 on C1D15.

Biomarker Assessment

Biomarkers will be assessed: levels of MSI, Tumor Mutant Burden (TMB), PD-L1 (tumor tissues), etc., and the condition of drug-related pathway mutations (including but not limited to PI3K, AKT, KEAP1, NFE2L2, STK11).

Tumor biomarker of subjects will be tested at Screening visit, Day 1 of each cycle and End of Treatment visit (serum carbohydrate antigen 125(CA125) must be quantitatively detected in ovarian cancer subjects, fetal protein (AFP) must be quantitatively detected in HCC subjects) to exploratively assess the relationship between the expression levels of tumor markers and the efficacy indicators. Changes of CA125 will also need to be assessed for ovarian cancer subjects based on the Gynecologic Oncology Coordinating Group (GCIG) criteria combined with RECIST 1.1. For subjects who have CA125 response, the CA125 will be retested after 4 weeks to confirm the response; If CA125 drops to normal after treatment, at least two additional CA125 tests will be performed a week apart to confirm PD (CA125 response and disease progression is defined by GCIG). When appropriate, correlation of drug exposures, adverse events and clinical outcomes will be analyzed.

Summary of statistics

Safety Analysis:

Safety assessment includes adverse events (AE), vital signs, physical examination, electrocardiogram (ECG), and laboratory tests. The incidence of DLT will be assessed. All treatment emergent adverse event (TEAE) will be summarized according to systemic organ classification and preferred terminology. TEAE will be summarized in terms of incidence, severity, and causality with the study drug. A descriptive summary of clinical laboratory tests and vital signs will be provided. All abnormal findings of clinical laboratory tests, vital signs, physical examination, and ECG will be listed.

Efficacy Analysis:

The efficacy assessment includes objective response rate (ORR), disease control rate (DCR), duration of response (DOR), progression-free survival (PFS) and overall survival (OS). ORR, DCR, DOR and PFS will be assessed by investigator based on RECIST1.1 or RANO criteria (only limited to GBM). ORR assessed by investigator based on RECIST1.1 or RANO criteria (only limited to GBM) will be used as the primary efficacy endpoint for the Dose Expansion Phase and will be summarized for all subjects receiving treatment. Kaplan-Meier method will be used to provide the OS, PFS, and DOR of all subjects receiving treatment. Exploratory efficacy will also be assessed by investigators based on iRECIST for solid tumors and iRANO criteria for GBM.

During the study treatment, the serum ADA data will be analyzed to assess the immunological effect of ATG-008 combined treatment with Toripalimab.

PK Analysis:

The PK data wil be presented in the form of graphs and/or tables with descriptive summaries. The PK parameters (AUC_{0-21}, $AUC_{0-\infty}$, C_{max}, T_{max}, $t_{½}$, clearance rate, distribution volume, etc.) will be presented in the form of tables by the dose level of ATG-008.

Exploratory Analysis:

Exploratory summary and analysis of ORR, DCR, DOR, and PFS assessed by the investigator according to the iRECIST will be performed.

Levels of MSI, TMB, PD-L1 (tumor tissues) or sPD-L1 (blood sample), etc., and the condition of drug-related pathway mutations (including but not limited to PI3K, Akt, KEAP1, NFE2L2, and STK11) of subjects will be descriptively analyzed and summarized.

Descriptive analysis and summary of AFP response rates will be performed in HCC subjects. Response rate of CA125 will also be analyzed based on GCIG criteria combined with RECIST 1.1 in ovarian cancer subjects.

Table 1 Study flowchart

Study cycle	Screening	Treatment（1 cycle=21 days ）							EOT	Safety follow-up[s]	Survival follow-up[t]
		Cycle 1			Cycle 2			≥Cycle 3			
**Visit （days）**	**-28 to -1**	**1**	**8**	**15**	**1**	**8**	**15**	**1**	**0-7d**	**30d**	**Every 12w**
**Time Window（days）**	**-**	**-**	**（±3d，Tumor evaluation±7d）***						**-**	**±7d**	**±14 d**
Informed Consent Form (ICF)	X										
Inclusion/Exclusion Criteria	X										
Demographics/Medical History/Concomitant Medications[a]	X										
Height/Weight[b]	X	X			X			X	X	X	
Physical Examination[c]	X	X			X			X	X	X	
Vital Signs[d]	X	X	X	X	X	X	X	X	X	X	
ECOG Performance Status	X	X			X			X	X	X	
Child-Pugh Classification (HCC specific)	X	X[e]			X			X	X	X	
HBV/HCV/HIV[f]	X	（X）[e]			（X）			（X）	（X）	（X）	
12-lead ECG	X	X[e]	Clinical indicated								
Complete Blood Count[g]	X	X[e]	X	X	X			X	X	X	
Blood Biochemistry Panel (Fasting Glucose Included)[h]	X	X[e]	X	X	X			X	X	X	
Urinalysis[i]	X	X[e]			X			X	X		
Coagulation[j]	X	X[e]			X			X	X		
ANA	X										
HbA1c	X	Every 2 cycles（CxD1）							X		
Thyroid function[k]	X	X[e]			X			X	X		
Myocardial enzyme[l]	X	X[e]	Clinical indicated								
AFP(HCC only)、CA125(Ovarian cancer only)[m]	X	X[e]			X			X	X		
Pregnancy tests[n]	X	X			X			X	X	X	
Echocardiograph	X										
Tumor assessment[o]	X	Every 6 weeks (±7 days) in the first 12 months of treatment, and performed once every 12 weeks (±7 days) after 12 months of treatment; Clinical indicated									
Pharmacokinetics[p]		X		X							
ADA[q]		Day1 from cycle 1-6，Day1 every 2 cycles after Cycle 6							X	X	
Tumor tissue MSI、TMB and PD-L1[r]	X										
sPD-L1[r]	X										
KPS（GBM only）	X	X			X			X	X		
Adverse events	X	X	X	X	X	X	X	X	X	X	X
Concomitant medication	X	X	X	X	X	X	X	X	X	X	
ATG-008 treatment		X	X	X	X	X	X	X			
Torpalimab treatment		X			X			X			
Survival status											X
Subsequent anti-tumor treatment									X	X	X

*Excluding pharmacokinetic testing

Abbreviations: ADA = Anti-Drug Antibody; AFP = Alpha-Fetoprotein; ANA = Anti-Nuclear Antibody; CA125 = Carbohydrate Antigen 125; C1D1 = Cycle 1 Day 1; ECG = Electrocardiogram; ECOG = Eastern Cooperative Oncology Group; HbA1c = Hemoglobin A1c; Anti-HBc = Hepatitis B Core Antibody; HBV = Hepatitis B Virus; HBsAg = Hepatitis B Surface Antigen; HCC = Hepatocellular Carcinoma; HCV = Hepatitis C Virus; HIV = Human Immunodeficiency Virus; MSI = Microsatellite Instability; sPD-L1 = Soluble Programmed Death-Ligand 1; TMB = Tumor Mutational Burden; TSH = Thyroid-Stimulating Hormone; T3 = Triiodothyronine; T4

= Thyroxine

a. Include treatment history for the primary diagnosis, encompassing prior systemic therapies, radiotherapy, and surgical interventions. The date of the most recent antitumor treatment must be documented. Complete medication records for the 28 days preceding the first dose of study drug (C1D1) must be reported.
b. Height will be measured only during screening. Body weight will be recorded at screening, on Day 1 of each cycle, at end-of-treatment, and during safety follow-up.
c. Comprehensive physical examinations will be conducted at screening and end-of-treatment visits. Symptom-directed physical exams will be performed during the study and safety follow-up.
d. Vital signs include blood pressure, pulse, body temperature, and respiratory rate; measured after the subject has rested in a supine or seated position for 5 minutes.
e. Tests performed within 7 days prior to the first dose of study drug do not need to be repeated at the C1D1 visit.
f. HBV serology and HIV testing will be performed during screening. Subjects with positive HBsAg, anti-HBc antibodies, or detectable HBV-DNA at screening will undergo HBV-DNA testing every cycle. If HBV-DNA increases (in subjects with detectable baseline HBV-DNA) or becomes detectable (in subjects with undetectable baseline HBV-DNA), qualitative HBsAg, HBeAg, and anti-HBc antibody testing will be performed.
For non-HCC subjects: Subjects with positive HBsAg or HCV antibodies during screening must undergo HBV-DNA quantification (≤2500 copies/mL or 500 IU/mL) and HCV-RNA testing (below the lower limit of quantification). Enrollment is permitted only after excluding active hepatitis B/C requiring treatment.HBV carriers, subjects with stable HBV (DNA ≤2500 copies/mL or 500 IU/mL after antiviral therapy), and cured HCV subjects are eligible.
For HCC subjects: Subjects with positive HBsAg and/or anti-HBc antibodies are eligible if:Baseline HBV-DNA <2000 IU/mL or 10^4 copies/mLIf HBV-DNA exceeds this threshold, antiviral therapy must reduce HBV-DNA to normal range for ≥2 weeks before enrollment, with continued antiviral treatment throughout the study. Subjects with prior antiviral therapy and normal baseline HBV-DNA must continue treatment during the study. HCV-RNA-positive subjects with normal liver function may enroll but must receive approved anti-HCV therapy.
g. Complete blood count (CBC) includes hemoglobin, hematocrit, mean corpuscular volume (MCV), mean corpuscular hemoglobin (MCH), mean corpuscular hemoglobin concentration (MCHC), white blood cell count (WBC), WBC differential, red blood cell count (RBC), lymphocytes, monocytes, neutrophils, eosinophils, basophils, and platelets. Reticulocytes will be tested only if clinically indicated.
h. Blood biochemistry includes sodium, potassium, chloride, bicarbonate, blood urea nitrogen (BUN)/urea, creatinine, glucose, calcium, phosphate, magnesium, ALT, AST, alkaline phosphatase (ALP), total bilirubin, lactate dehydrogenase (LDH), total protein, albumin, amylase, lipase, creatine kinase (CK), and uric acid.
i. Urinalysis includes bilirubin, glucose, occult blood, ketones, pH, protein, specific gravity, and urobilinogen. Microscopic examination will be performed only if clinically indicated.
j. Coagulation tests include prothrombin time (PT), international normalized ratio (INR), and activated partial thromboplastin time (APTT). If PT/APTT testing is unavailable, thrombin time (TT) may be used as an alternative.
k. Free T3, free T4, and TSH will be tested at screening, Day 1 of each cycle, and end-of-treatment visits.
l. Cardiac enzyme testing (serum CK and CK-MB) will be performed at screening, Cycle 1 Day 1, and as clinically indicated.
m. AFP (for HCC subjects) and CA125 (for ovarian cancer subjects) will be quantified at screening, Day 1 of each cycle, and end-of-treatment visits. Ovarian cancer subjects will also be evaluated for CA125 changes per GCIG criteria combined with RECIST 1.1. CA125 response requires confirmation by retesting 4 weeks later. For subjects with normalized CA125 after treatment, two additional CA125 tests ≥1 week apart are required to confirm progression (see Appendix 11 for GCIG criteria).
n. Serum pregnancy tests (hCG) will be performed for women of childbearing potential (WOCBP) at screening and end-of-treatment visits. Urine or blood pregnancy tests will be conducted on Day 1 of each cycle and during safety follow-up. Positive urine tests require serum confirmation.
o. All subjects must undergo chest, abdominal, and pelvic CT/MRI scans, plus brain MRI at screening. Subjects with bone lesions require confirmatory bone scans. Baseline tumor assessments must be completed ≤28 days before dosing, ideally close to dosing initiation. Tumor evaluations (excluding GBM) will follow RECIST 1.1

and iRECIST. GBM evaluations will use RANO and iRANO criteria. Imaging (CT/MRI) will be performed:

- First scan at 6 weeks post-dose.
- Every 6 weeks ±7 days for the first 12 months.
- Every 12 weeks ±7 days after 12 months.
- Continued until radiographically confirmed progression, new antitumor therapy, death, or consent withdrawal.

p. **Dose-escalation phase only**: PK blood samples for ATG-008 will be collected at:

- Pre-dose (15 minutes before first ATG-008 dose).
- 0.5±0.08, 1±0.17, 1.5±0.17, 3±0.17, 5±0.25, 8±0.5, 24±1, and 48±1 hours post-dose.
- C1D15: Pre-dose and 0.5±0.08, 1±0.17, 1.5±0.17, 3±0.17, 5±0.25, 8±0.5, and 24±1 hours post-dose.

q. For the first 6 treatment cycles, blood samples will be collected ≤60 minutes before toripalimab administration. Thereafter, samples will be collected every 2 cycles (Day 1 of treatment cycles) until end-of-treatment. Samples will also be collected at end-of-treatment and safety follow-up visits.

r. Tumor tissue will be collected for MSI, TMB, PD-L1, and pathway mutation analyses (e.g., PI3K, AKT, KEAP1, NFE2L2, STK11). Approximately 10 mL blood will be collected for sPD-L1 testing as needed.

s. Safety follow-up will occur 30±7 days after the last dose of study drug. Subjects discontinuing due to drug-related adverse events (AEs) will be followed until AEs resolve to Grade 0-1 or stabilize.

t. After the final safety follow-up, survival status will be assessed every 12 weeks ±14 days via phone or in-person contact until death, loss to follow-up, consent withdrawal, study termination, or sponsor-directed discontinuation. Data on survival, subsequent antitumor therapy, and drug-related serious AEs will be collected during these contacts.

Abbreviation

**Abbreviation**	**Full English name**
ADA	Anti-Drug Antibody
AE	Adverse Event
AFP	Alpha-fetoprotein
AKT	Serine/Threonine Kinase
ALT	Alanine Aminotransferase
ANA	Anti-Nuclear Antibody
ANC	Absolute Neutrophil Count
AST	Aspartate Aminotransferase
ATG-008	Dual mTOR Kinase Inhibitor (formerly CC-223)
AUC	Area Under the Curve
AUC_{0-21}	Area Under the Curve from Day 1 to Day 21
BCLC	Barcelona Clinic Liver Cancer Staging System
CA125	Carbohydrate Antigen 125
CDE	Center for Drug Evaluation
C_{max}	Maximum Serum Concentration
CR	Complete Response
CT	Computed Tomography
CTLA-4	Cytotoxic T-Lymphocyte Associated Protein 4
C_{trough}	Trough Concentration
CYP	Cytochrome P450
DCR	Disease Control Rate
DLBCL	Diffuse Large B-cell Lymphoma
DLT	Dose-Limiting Toxicity
DOR	Duration of Response
ECG	Electrocardiogram
ECOG	Eastern Cooperative Oncology Group
eCRF	Electronic Case Report Form
EGFR	Epidermal Growth Factor Receptor
FDA	Food and Drug Administration
GBM	Glioblastoma
GCIG	Gynecologic Cancer InterGroup
GCP	Good Clinical Practice
HbA1c	Hemoglobin A1c
HBcAb	Hepatitis B Core Antibody
HBsAg	Hepatitis B Surface Antigen
HBV	Hepatitis B Virus
HCC	Hepatocellular Carcinoma
hCG	Human Chorionic Gonadotropin
HCV	Hepatitis C Virus
HIV	Human Immunodeficiency Virus
ICF	Informed Consent Form
ICH	International Council for Harmonisation of Technical Requirements for Pharmaceuticals for Human Use
IEC	Independent Ethics Committee
INR	International Normalized Ratio
irAE	Immune-Related Adverse Event
iRANO	Immunotherapy Response Assessment for Neuro-Oncology
IRB	Institutional Review Board
iRECIST	Immune RECIST

KPS	Karnofsky Performance Status
MedDRA	Medical Dictionary for Regulatory Activities
mITT	Modified Intention-To-Treat
MRI	Magnetic Resonance Imaging
MSI	Microsatellite Instability
MTD	Maximum Tolerated Dose
mTOR	Mammalian Target of Rapamycin
NCCN	National Comprehensive Cancer Network
NCI-CTCAE	National Cancer Institute Common Terminology Criteria for Adverse Events
NMPA	National Medical Products Administration
NET	Neuroendocrine Tumor
NSCLC	Non-Small Cell Lung Cancer
ORR	Objective Response Rate (CR+PR)
OS	Overall Survival
pAKT	Phosphorylated AKT
PD-1	Programmed Cell Death Protein-1
PD-L1	Programmed Death-Ligand 1
PD-L2	Programmed Death-Ligand 2
PET	Positron Emission Tomography
PFS	Progression-Free Survival
PI3K	Phosphatidylinositol 3-Kinase
PK	Pharmacokinetics
PR	Partial Response
PT	Prothrombin Time
PTEN	Phosphatase and Tensin Homolog
PTT	Partial Thromboplastin Time
QD	Quaque Die
QTc	Corrected QT Interval
Q3W	Every 3 Weeks
RANO	Response Assessment in Neuro-Oncology
RECIST	Response Evaluation Criteria in Solid Tumors
RP2D	Recommended Phase 2 Dose
SD	Stable Disease
SMC	Safety Monitoring Committee
sPD-L1	Soluble Programmed Death-Ligand 1
$T_{1/2}$	Half-life
T3	Triiodothyronine
T4	Thyroxine
TEAE	Treatment-Emergent Adverse Event
TMB	Tumor Mutational Burden
T_{max}	Time to Maximum Concentration
TRA	Total Radioactivity
TSH	Thyroid-Stimulating Hormone
ULN	Upper Limit of Normal

1. Background

1.1. Medical background

According to the 2018 China Cancer Report, China had the highest incidence and mortality rates of cancer globally (Freddie, 2018). The latest report from the World Health Organization (WHO) in 2018 revealed a global increase of 18.1 million new cancer cases and 9.6 million deaths (5.4 million in males and 4.2 million in females), further exacerbating the global cancer burden. Nearly half of the world's new cancer cases and over half of cancer deaths occurred in Asia in 2018. Among them, China, as a populous country, accounted for a significant portion of cancer incidence and mortality in Asia, with one country's cancer incidence nearly equivalent to that of the entire Americas, and cancer-related deaths surpassing those of the entire Europe (International Agency for Research on Cancer, 2018). Based on data collected by the China National Cancer Center between 2000 and 2011, it is estimated that China had 42.92 million new cancer cases and 28.14 million cancer deaths in 2015 (Chen, 2016). Looking at the mortality figures, lung cancer remains the leading cause of death (1.8 million deaths, accounting for 18.4% of the total), followed by colorectal cancer (881,000 deaths, accounting for 9.2%), gastric cancer ranking third (783,000 deaths, accounting for 8.2%), followed by liver cancer (782,000 deaths, accounting for 8.2%), and female breast cancer ranking fifth (627,000 deaths, accounting for 6.6%) (World Cancer Report, 2018). Therefore, the development of new cancer treatment methods is urgently needed.

1.2. Disease background

1.2.1. Hepatocellular carcinoma

Globally, hepatocellular carcinoma (HCC) accounts for 85-90% of all primary liver cancers, ranking as the third leading cause of cancer-related deaths worldwide and the second leading cause of cancer-related deaths in males. The incidence of HCC varies significantly geographically, with higher rates in Asia and sub-Saharan Africa, while rates in the United States and Europe are steadily increasing (Parkin, 2005; El Serag, 1999), possibly due to higher rates of liver cirrhosis caused by hepatitis C virus (HCV) infection and alcohol/non-alcoholic fatty liver disease. Hepatitis B virus (HBV) is a major risk factor for HCC in Southeast Asia and Africa. Currently, chronic HBV infection accounts for approximately 80% of all newly diagnosed HCC cases in Asia (Raza, 2007). Over 50% of new cases and deaths occur in China (Parkin, 2005), making it the second most common cancer (Yang, 2005a).

Sorafenib has been the only first-line standard systemic therapy for advanced HCC patients for a long time. On September 4, 2018, lenvatinib was approved by the China National Medical Products Administration (NMPA) for first-line treatment of unresectable HCC. In December 2017, regorafenib was approved by the NMPA for use in HCC patients previously treated with sorafenib. Additionally, in January 2019, cabozantinib was approved by the United States Food and Drug Administration (FDA) for HCC patients previously treated with sorafenib. Prior to this, nivolumab and pembrolizumab were also FDA-accelerated approved for HCC patients previously treated with sorafenib. Although there have been significant advances in the pharmacological treatment of advanced liver cancer in recent years, the currently available treatments provide only modest survival benefits for HCC patients. However, the overall prognosis remains poor. The 5-year survival rate for advanced disease globally is only 3% to 5% (Parkin, 2005). In Western countries, potentially curative treatments are applicable to only 30% to 40% of patients (Llovet, 2003). The recurrence rate after surgery is approximately 70%, and patients who experience recurrence have a poor prognosis (Llovet, 2003). The median overall survival (OS) for stage B and stage C HCC patients after

treatment is 8-14 months. Therefore, new treatment methods are still needed for advanced HCC patients who cannot undergo local treatment.

1.2.2. Neuroendocrine tumor

Neuroendocrine tumors (NETs) are rare tumors, with an estimated incidence of approximately 7 cases per 100,000. The most common origins are the gastrointestinal pancreatic system (approximately 70%) and the lungs (approximately 20%), with fewer originating from the urinary reproductive system, female gynecological system, and Merkel cells in the skin. According to the WHO-2010 classification, gastrointestinal pancreatic neuroendocrine tumors are classified into Grade 1 (G1, Ki67 20%), with all G3 tumors referred to as neuroendocrine carcinomas. The treatment of neuroendocrine tumors varies greatly depending on the location of the primary lesion, morphological differentiation, and grading. For unresectable advanced low- to intermediate-grade neuroendocrine tumors, key options include somatostatin analogs (SSAs), tyrosine kinase inhibitors (TKIs), mammalian target of rapamycin (mTOR) inhibitors, and peptide receptor radionuclide therapy (PRRT) (Lee, 2018). Currently, the molecular targeted drugs for the treatment of neuroendocrine tumors are mTOR inhibitor everolimus and multi-target tyrosine kinase inhibitor sunitinib, both of which have completed Phase III clinical trials in patients with advanced pancreatic neuroendocrine tumors and have been shown to prolong progression-free survival (PFS) in trial participants. In addition, the expression of programmed death-ligand 1 (PD-L1) and PD-1 is associated with high-grade tumors (such as G3 and neuroendocrine carcinomas), and PFS and OS are poor. In terms of anti-tumor treatment for higher-grade G3 neuroendocrine tumors, exploring the effectiveness and safety of everolimus combined with chemotherapy as first-line treatment, everolimus monotherapy as maintenance treatment after effective first-line treatment, or as second- line treatment after first-line treatment failure is the main direction of investigation (Zhang Yu, 2017). In summary, the treatment of neuroendocrine tumors requires multiple approaches, but further research is needed to determine the effectiveness and safety of their combination with other drugs and resistance mechanisms.

1.2.3. Gynecological tumors in advanced stage or with metastasis (endometrial cancer, cervical cancer, ovarian cancer)

Endometrial cancer ranks second among malignant tumors of the female reproductive system in China. According to statistics from the National Cancer Center in 2015, the incidence rate was 63.4 per 100,000 and the mortality rate was 21.8 per 100,000. Sixty-seven percent of endometrial cancer patients are diagnosed with disease confined to the uterus, while regional and distant diseases account for 21% and 8%, respectively. Second-line treatments (typically radiotherapy and/or surgery or systemic therapy) are effective for recurrent endometrial cancer involving the vagina or pelvis (Zhou Qi, 2020). Despite early detection being possible for endometrial cancer and favorable prognosis after treatment for most patients, some remain at high risk of recurrence, contributing to a relatively high mortality rate (Li Haiying, 2020). A study on 108 late-stage or recurrent endometrial cancer patients treated with bevacizumab combined with chemotherapy showed improved progression-free survival (PFS) but did not significantly increase overall survival. Another study indicated that recurrent endometrial cancer patients (133 cases) treated with the multi-targeted TKI lenvatinib demonstrated efficacy with generally good tolerability. Additionally, the PD-1 antibody pembrolizumab exhibited good safety and durable anti-tumor activity in a subgroup of late-stage PD- L1 positive endometrial cancer patients. In summary, the prognosis for patients with advanced or recurrent endometrial cancer is poor, and treatment strategies for malignancies progressing after standard therapy require further exploration (She Yuanping, 2020).

Cervical cancer ranks fourth globally in both incidence and mortality rates among women. It is the second most common female-specific malignancy in China, with incidence and mortality rates concentrated among women aged 20-50 years. Current treatments include radiotherapy, chemotherapy, and surgery. Surgery is restricted to women with early-stage disease or those who have lost fertility. Cervical cancer is often treated with combined chemotherapy (platinum-based)

and radiotherapy. However, many tumors develop chemoresistance, leading to treatment failure. Despite advances in screening and preventive vaccines, over half of cervical cancer cases are diagnosed at advanced stages due to limited treatment options and severe side effects (Wang, 2019). The National Comprehensive Cancer Network (NCCN) guidelines recommend pembrolizumab for second-line treatment of recurrent or metastatic cervical cancer with PD-L1 positivity or mismatch repair deficiency (dMMR)/high microsatellite instability (MSI-H). Single-agent immunotherapy has limited efficacy for most tumors. Combination immunotherapy with surgery, radiation, or chemotherapy is a hot topic in clinical trials, including studies on the efficacy and safety of pembrolizumab combined with synchronous chemoradiation for locally advanced cervical cancer, pembrolizumab combined with paclitaxel-platinum chemotherapy for late-stage or recurrent cervical cancer, and atezolizumab combined with bevacizumab (anti-angiogenic targeted therapy) for recurrent or metastatic cervical cancer. More new drugs or treatment methods are needed for clinical treatment of advanced and recurrent cervical cancer (Li Haiying, 2020).

Ovarian cancer has approximately 52,100 new cases and 22,500 deaths annually in China, with epithelial ovarian cancer accounting for about 90%. Due to the lack of effective screening methods and difficulties in early diagnosis, 70% of ovarian cancer patients are diagnosed at advanced stages, and 50%-70% experience recurrence within 2 years, with a less than 30% survival rate at 5 years. Common diagnostic methods for ovarian cancer include tumor marker testing and imaging examinations, with carbohydrate antigen 125 (CA125) and human epididymis protein 4 (HE4) widely used in epithelial ovarian cancer. However, their sensitivity and specificity do not meet the requirements for early diagnosis. The traditional treatment for ovarian cancer primarily involves surgery with adjuvant chemotherapy, but the development of chemotherapy resistance significantly affects patient prognosis (Wang Xiaoni, 2020).

1.2.4. Glioblastoma multiforme

Glioblastoma multiforme (GBM), also known as glioblastoma, originates from glial cells in the brain and is the most common intracranial tumor. WHO classifies brain gliomas into grades I-IV, with grades I and II being low-grade and III and IV being high-grade. Grade IV glioblastomas account for the majority of deaths from primary brain tumors. The incidence rate of glioblastoma in China is 3-6.4 per 100,000 annually, with 30,000 deaths each year, predominantly affecting males. The 5-year mortality rate ranks third among all cancers, following pancreatic and lung cancers. Clinical trials show a median overall survival (OS) of approximately 15-18 months, with less than 10% surviving 5 years. The prognosis for GBM is poor, necessitating standard treatment strategies including maximal surgical resection, radiotherapy combined with temozolomide synchronous chemotherapy, sequential temozolomide chemotherapy and/or tumor treatment electric fields. Currently, clinical assessment standards include Karnofsky performance status (KPS), Eastern Cooperative Oncology Group (ECOG) score, and WHO quality of life assessment score. Bevacizumab is the first targeted drug recommended for recurrent GBM; however, it only extends OS. Additionally, its role in WHO II/III gliomas is unclear. The TAVAREC study compared the efficacy of bevacizumab combined with TMZ versus TMZ monotherapy in recurrent WHO II/III gliomas without 1p/19q codeletion, with results showing no statistical difference in survival between the two groups (Van, 2018; Li Depei, 2020). GBM treatment faces challenges related to central nervous system localization, the blood-brain barrier, and high heterogeneity within and between tumors (Wang, 2019). Both domestic and international guidelines recommend clinical trial participation for recurrent cases, highlighting the need to explore more effective new therapies.

1.3. Research Drug Introduction

1.3.1. ATG-008

1.3.1.1. Rapamycin Target Protein

ATG-008 (formerly known as CC-223, CC0482223) is a potent, selective mammalian target of rapamycin (mTOR) kinase inhibitor currently in development for the treatment of solid tumors and hematologic malignancies. It is a dual inhibitor of both mTORC1 and mTORC2 complexes, with the

potential to significantly reduce resistance caused by phosphorylation of AKT (serine/threonine kinase).

mTOR is a serine/threonine kinase associated with the phosphatidylinositol 3-kinase (PI3K) family of lipid kinases. Its functions include serving as a sensor for mitogens, energy, and nutrient levels, as well as a central regulator of cell growth. mTOR exists in two complexes: mTORC1, which complexes with raptor and is sensitive to rapamycin, and mTORC2, which complexes with rictor and is insensitive to rapamycin (Kim, 2002; Sarbassov, 2004). mTORC1 integrates signals from growth factor receptors and cellular nutrient status to control the level of messenger RNA translation by regulating the activity of key translational components. The function of mTORC2 has not been elucidated to the same extent as mTORC1 (Feldman, 2009). However, mTORC2 is thought to regulate growth factor signaling pathways by phosphorylating kinases such as AKT. Activation of AKT can promote cell survival through various mechanisms, including inhibition of apoptosis, promotion of glucose uptake, and regulation of cell metabolism (Gibbons, 2009). In many cancers, inappropriate activation of the PI3K-AKT pathway is crucial for cancer cell growth and survival (Engelman, 2009). Therefore, mTOR inhibitors are being developed as potential therapeutic agents for various cancers. Rapamycin and its analogs can inhibit the mTORC1 complex alone and stimulate the upstream kinase AKT by inhibiting the negative feedback loop between p70S6 kinase and insulin receptor substrate 1. This mechanism can partially explain the resistance of most cancer cell lines and tumors to rapamycin analogs (Gibbons, 2009). Thus, simultaneous inhibition of mTORC1 and mTORC2 by mTOR kinase inhibitors is expected to inhibit AKT rather than activate it.

The PI3K/AKT/mTOR pathway is a crucial signaling pathway driving cell growth and proliferation in HCC (hepatocellular carcinoma). Homozygous deletion of phosphatase and tensin homolog (PTEN) expression, often due to promoter hypermethylation, is a common alteration in 30% to 50% of HCC cases and is associated with activation of the mTOR pathway, activation of vascular endothelial growth factor (VEGF), and a more aggressive phenotype (Yang, 2005b; Mi, 2006; and Hayashi, 2009).

Analysis of gene alterations and protein expression levels in 314 HCC samples revealed activation of the insulin-like growth factor pathway, overexpression of vascular endothelial growth factor, loss of PTEN, and activation of the mTOR signaling pathway in 50% of the samples. Increased copy numbers of Rictor, associated with mTOR pathway activation, were observed in 25% of HCC cases. Activation of the TORC2 pathway, associated with increased expression of phosphorylated AKT (pAKT), was observed in most HCC cases and was associated with vascular invasion, higher tumor staging and grading, and poorer prognosis (Villanueva, 2008a).

Rapamycin has been shown to inhibit proliferation of HCC cell lines in vitro and inhibit tumor growth and angiogenesis in vivo (Semela, 2007). Similar results were observed with rapamycin analogs such as everolimus in xenograft models (Huynh, 2009; Villanueva, 2008a). These studies provide a solid theoretical basis for clinical studies of mTOR pathway inhibitors in HCC patients, as well as compounds with inhibitory activity against both TORC1 and TORC2 complexes.

Although the mechanism of sorafenib resistance has not been fully elucidated, preclinical studies suggest involvement of epidermal growth factor receptor (EGFR), signal transducer and activator of transcription 3 (STAT3), and activation of the PI3K/AKT signaling pathway. HCC cell lines exhibiting acquired resistance to sorafenib show increased levels of pAKT and p85 (PI3K regulatory subunit) and downregulation of PTEN, consistent with activation of the mTOR pathway. Additionally, treatment of resistant cells with allosteric AKT inhibitors restores sensitivity to sorafenib-induced apoptosis (Chen, 2012). In another study, acquired sorafenib-resistant HCC cells were found to have abnormal activation of EGFR/human epidermal growth factor receptor 3 and overexpression of several EGFR ligands. These enhanced autocrine/paracrine loops lead to constitutive activation of extracellular signal-regulated kinase and AKT and enhance sensitivity of cells to gefitinib (Blivet-VanEggepoel, 2012). These data provide additional support for the use of

mTOR-targeted drugs that simultaneously inhibit TORC1 and TORC2 complexes and thus block the activation of pAKT in the treatment of sorafenib-resistant HCC.

The PI3K/Akt and mTOR signaling pathways play important roles in controlling the cell cycle and growth of cancer cells, and their activation is involved in the pathogenesis of NETs. Preclinical models of NETs have reported the anti-proliferative effects of mTOR pathway inhibition, making it a potential therapeutic target (Wolin, 2013; Chan, 2014). Several phase II/III clinical studies have evaluated the efficacy and safety of oral mTOR pathway inhibitors, such as everolimus, in patients with advanced NETs from different sources. In the RADIANT-3 trial, significant clinical benefits were observed in many patients with progressive pancreatic neuroendocrine tumors (Yao, 2011). On May 5, 2011, the FDA approved everolimus as an anti-tumor drug for the treatment of pancreatic neuroendocrine tumor disease. The RADIANT-4 trial investigated the efficacy of everolimus in 302 patients with advanced gastrointestinal and lung NETs, showing a significant improvement in median progression-free survival (PFS) compared to placebo, with a PFS of 7.1 months (11.0 vs. 3.9 months) and a 52% reduction in the risk of disease progression or death (Lee, 2018).

Furthermore, studies have shown that the activation of the PI3K/Akt signaling pathway is closely associated with various human cancers, including breast, ovarian, endometrial malignancies, and GBM. For example, the PI3K/Akt/mTOR pathway is aberrantly activated in approximately 70% of ovarian cancers (Wang Xiaoni, 2020). Additionally, the activation of the PI3K/Akt pathway is associated with incomplete response in cervical cancer (Schwarz, 2012). The frequent expression of the PI3K/Akt/mTOR pathway in cervical cancer suggests that it may be a potential therapeutic target and a new biomarker for assessing the risk of cervical cancer. Moreover, the mTOR signaling pathway plays a crucial role in cerebral vasodilation. The control of protein translation by mTORC1 is of significant importance in synaptic plasticity, memory, and learning (Neasta, 2010). Excessive activation of the mTOR signaling pathway is present in most GBMs. Recent studies have indicated that the PI3K signaling pathway regulated by mTOR could be a potential molecular target in GBM patients (Duzgun, 2015). Next-generation adenosine triphosphate (ATP)-competitive mTOR kinase inhibitors, such as CC214-1 and CC214-2, inhibit the growth of GBM by blocking the activity of mTORC2 in vitro and in vivo. Sensitivity to CC214 compounds significantly increases in GBM cells with EGFRvIII and PTEN deficiencies (Gini, 2013).

Preclinical research results (PD1752, 1753) show that ATG-008, as a single agent, inhibits the growth of endometrial cancer cells (4 endometrial cancer cell lines) and has a growth-inhibitory effect on ovarian cancer cell lines. The baseline level of pAKT in the cell lines is unrelated to the sensitivity to CC-223. Combination treatment with ATG-008 and erlotinib demonstrates synergistic anti-proliferative effects in a cell line (RL95-2) with high expression of EGFR and no PTEN (combination index (CI) values of 0.52 and 0.25, n=2; confirmed by fractional product method). These data provide supportive evidence for the anti-tumor activity of mTORC1/2 inhibitors.

1.3.1.2. Clinical Efficacy Data of ATG-008

To date, five clinical studies of ATG-008 have been completed, with two ongoing. Three clinical pharmacology studies (CC-223-CP-001, CC-223-CP-002, and CC-223-CP-003) involved 50 healthy subjects. The studies CC-223-ST-001, CC-223-NSCL-001, and the ongoing study CC-223-DLBCL-001 included a total of 402 subjects with various solid and hematologic malignancies. Additionally, an international multicenter clinical study (ATG-008-HCC-001) is being conducted in mainland China, South Korea, and Taiwan, which will include 30 subjects with HBV+ and unresectable HCC who have previously received at least one systemic treatment regimen.

In the CC-223-CP-001 study, 18 healthy adult male subjects received a single dose of 20 mg ATG-008 capsule, 4 tablets of 5 mg ATG-008 tablet, and a single dose of 20 mg tablet, with a washout period of 7 days between each treatment. The 20 mg ATG-008 capsule, 5 mg ATG-008 tablet, and 20 mg ATG-008 tablet showed comparable exposure to ATG-008, indicating similar bioavailability. ATG-008 was rapidly absorbed, and the average concentrations of ATG-008 and M1

reached peak levels within 1 to 3 hours after administration. The average terminal half-lives of ATG-008 and M1 were 6 and 14 hours, respectively.

The study CC-223-CP-002 was conducted in 18 healthy adult male subjects (6 subjects participated in Part 1, and 12 subjects participated in Part 2). In Part 1, the subjects received a single dose of 20 mg ATG-008 capsule orally, which contained a trace amount of [14C] ATG-008 solution. In Part 2, the subjects received a single dose of 20 mg ATG-008 orally under fasting and fed conditions. ATG-008 was well absorbed systemically, with less than 2% of the administered dose recovered in urine and feces. ATG-008 accounted for approximately 16% of the total radioactivity exposure in circulation. ATG-008 was extensively metabolized into seven metabolites measured in urine and feces: M1, M2, M4, M7, M8, M11, and M13. Among these seven metabolites, only M1, M2, and M13 could be measured in plasma. M1, formed through demethylation reaction, was the major circulating entity in plasma, accounting for approximately 77% of the total radioactivity exposure in circulation. Renal elimination was the main excretion pathway for radioactivity. When healthy adult male subjects took a single dose of 20 mg ATG-008 with or without a high-fat meal, there was no significant change in the exposure of ATG-008 and M1. When ATG-008 was taken after a meal, the time to reach peak blood concentrations (T_{max}) of ATG-008 and M1 was prolonged by 1 hour and 3 hours, respectively. These differences are expected to have minimal clinical impact.

In the study CC-223-CP-003, 14 healthy adult male subjects received ketoconazole (a potent cytochrome P[CYP]3A4 inhibitor) in combination with a single dose of 20 mg ATG-008. After co-administration of a single dose of 20 mg ATG-008 with the CYP3A4 inhibitor ketoconazole (400 mg), the exposure of ATG-008 and M1 increased moderately, by approximately 60% and 70%, respectively. This moderate increase was much weaker than the significant increase (>3-fold) observed in in vitro CYP3A4 inhibition analysis, suggesting the presence of compensatory non- CYP3A4-mediated metabolic/elimination pathways that attenuate the inhibitory effect on overall ATG-008 metabolism and elimination mediated by CYP3A4. These findings can be extrapolated to the use of ATG-008 in combination with other moderate to strong CYP3A4 inhibitors, supporting the need for moderate clinical adjustments rather than prospective dose adjustments for ATG-008.

The first Phase I/II dose escalation and expansion clinical study (CC-223-ST-001) evaluating the safety, tolerability, pharmacokinetics (PK), and preliminary efficacy of ATG-008 in human solid tumors and hematologic malignancies was completed in November 2016. In the dose escalation phase (Phase I), 28 subjects received daily once-daily (QD) dosing at five dose levels: 7.5 mg (n=1), 15 mg (n=2), 30 mg (n=9), 45 mg (n=9), and 60 mg (n=7). The dose of 45 mg QD was selected for the expansion phase (Phase II) of this study, which involved 198 subjects and included seven tumor types: HCC, NSCLC, pleomorphic glioblastoma, hormone receptor-positive breast cancer, non- pancreatic origin neuroendocrine tumors, diffuse large B-cell lymphoma (DLBCL), and multiple myeloma.

In the CC-223-ST-001-B (dose expansion) study, subjects received ATG-008 treatment at a dose of 30 mg or 45 mg per day. In the solid tumor cohort (excluding GBM), the objective response rate (ORR) was 5.6% (95% CI: 2.4%, 10.7%). The highest ORR was observed in the hormone receptor- positive breast cancer (HRPBC) cohort at 11.8%, compared to HCC (5.7%), NET (4.3%), and NSCLC cohorts (3.8%). Among the treated subjects, 5.6% achieved a partial response (PR), 55.9% had stable disease (SD), and 9.8% experienced disease progression. The overall disease control rate (DCR) was 61.5% (95% CI: 53.0%, 69.5%), with the highest DCR observed in the NET cohort (78.7%; 95% CI: 64.3%, 89.3%). Due to the inclusion of salvage tumor resection in the study, tumor efficacy assessment before and after treatment was not possible for the GBM cohort. At the 6-month mark of the study, all GBM subjects (13 cases) had experienced disease progression.

Efficacy of ATG-008 in HCC:

In the CC-223-ST-001-B study, analysis of efficacy data for longer-term treatment showed an ORR of 5.7% (95% CI: 1.2%, 15.7%) in the HCC cohort. Based on HBV status, the ORR for HBV- positive subjects was 25.0% (95% CI: 5.5%, 57.2%), while it was 0% for HBV-negative subjects.

Three subjects (5.7%) achieved a PR as the best response (all three were HBV-positive), and 26 subjects (49.1%) had SD as the best response (8 cases [66.7%] were HBV-positive, 18 cases [43.9%] were HBV-negative). Six subjects (11.3%) experienced disease progression as the best overall response (1 case [8.3%] was HBV-positive, 5 cases [12.2%] were HBV-negative). The DCR in the HCC cohort was 54.7% (95% CI: 40.4%, 68.4%). Among HBV-positive subjects, the DCR was 91.7% (95% CI: 61.5%, 99.8%), while among HBV-negative subjects, it was 43.9% (95% CI: 28.5%, 60.3%).

Efficacy of ATG-008 in NET:

In the CC-223-ST-001-B study, 47 subjects with NET received a dose of 30 mg (n=23) or 45 mg (n=24) of ATG-008. The overall response rate (ORR) in the treatment group was 6.4% (95% CI: 1.3%, 17.5%), with 34 subjects (72.3%) achieving stable disease (SD) as the best response. The disease control rate (DCR) was 78.7% (95% CI: 64.3%, 89.3%). Compared to baseline, the target tumor shrinkage rate was 63.8% (95% CI: 48.5%, 77.3%). The median duration of overall response for the 3 subjects who achieved a partial response (PR) was 253.0 days. Among the 34 subjects with the best SD response, the median duration of SD was 296.5 days. Seventeen subjects had SD lasting over one year. The median progression-free survival (PFS) was 19.5 months (95% CI: 10.4, 28.5), and the median overall survival (OS) was not reached.

In summary, this study provides preliminary evidence of the broad antitumor activity of ATG-008 against various solid tumors and hematologic malignancies.

1.3.1.3. Safety Research data of ATG-008 (C-223)

In general, the most common treatment-emergent adverse events (TEAE) in the ATG-008 study included fatigue, diarrhea, nausea, vomiting, hyperglycemia and anorexia. These TEAE occur in a large proportion of subjects in the higher dose cohort, so they may be dose-dependent. These TEAE are also most often attributed by researchers to ATG-008 treatment. Most TEAE occurs in the early stages of treatment and can be managed through drug therapy interventions or by reducing doses and interrupting treatment. Selected adverse events known to be related to the mechanism of action (AE (i.e., infection, septicemia, pneumonia and lung inflammation)), known category effects or the frequency of TEAE observed so far are consistent with expectations.

Date of data deadline to August 28, 2019 Adverse drug reactions to CC-223 (ATG-008) reported in the Celgene study included blood and lymphoid disorders (neutropenia, thrombopenia), gastrointestinal disorders (stomatitis, nausea, vomiting, diarrhea, pancreatitis), systemic disorders and site reactions (fatigue / weakness, fever, peripheral edema), infections (septicemia, infection, pneumonia), metabolic and nutritional disorders (anorexia, loss of appetite, Dehydration, hyperglycemia, hypokalemia, hyponatremia, hypophosphatemia, hypercholesterol, hypertriglyceridemia), kidney and urinary system diseases (elevated serum creatinine, renal insufficiency, acute renal failure and acute tubular necrosis), respiratory system, chest and mediastinal diseases (inflammation of lung tissue) and skin and subcutaneous tissue diseases (rash, acne-like dermatitis).

In hematology, chemistry, urine testing, immunology, thyroid or vital signs, there were no unexpected trends or clinically important post-baseline findings. The changes observed in laboratory measurements (including glucose, insulin, C-peptide, CD4+ and CD8+) were consistent with those expected according to the mechanism of mTOR pathway inhibition. When there are medical indications, use hypoglycemic drugs to control hyperglycemia. Based on the changes of electrocardiogram (ECG) or left ventricular ejection fraction (LVEF), no cardiac problems were found. In general, the side effects of ATG-008 are similar to those of other drugs in the type of mTOR pathway inhibitors.

Overall, the safety of ATG-008 in healthy subjects was consistent with that in cancer subjects. In addition, the safety of ATG-008 was consistent with the published results of other drugs targeting mTOR and related cellular pathways, as well as with the preclinical toxicological results of ATG- 008.

1.3.1.4. Data of Pharmacokinetics of CC-223-ST-001

The preliminary competitive results of phase I of CC-223-ST-001 study showed that ATG-008 could be absorbed rapidly and reached the peak plasma level within 1 to 3 hours after single and multiple daily administration of ATG-008 (peak time of plasma concentration, T_{max}). In the range of 30 mg to 60 mg, the maximum blood concentration (C_{max}) and the area under the blood concentration-time curve (AUC) seemed to increase linearly with the dose. The terminal half-life of ATG-008 is about 4.86 to 5.64 hours. After repeated administration of multiple doses, the plasma accumulation rate of ATG-008 was very small (< 25%). There was no significant difference between the total exposure of ATG-008 in human body and that observed after oral dose was equal to or close to the maximum tolerable dose of human beings (45 mg QD). The plasma exposure level of M1, the main metabolite with pharmacological activity, was about 15 times higher than that of the parent compound.

Plasma concentrations of ATG-008 and M1 were measured during the dose expansion period (II phase) of the CC-223-ST-001 study in all subjects who used 30 or 45 mg QD ATG-008. After oral administration of single and multiple doses of ATG-008 at a dose of 30 mg/d and 45 mg/d, it could be absorbed rapidly, and the median T_{max} reached the maximum plasma concentration at 1.02 × 2.96 hours. At the dose level of 30 mg to 45 mg, the overall exposure dose ($AUC_{0-\infty}$) after a single oral administration of ATG-008 seemed to increase linearly with the dose. At the dose level of 30 mg to 45 mg, C_{max} also seemed to increase linearly with the dose after a single oral administration. M1 reached the maximum plasma concentration at 3.08 ≤ 6.13 hours of median T_{max}. At the dose level of 30 mg to 45 mg, the total exposure dose of M1 (AUC_{tot}) seemed to increase linearly with the dose after a single oral administration. At the dose level of 30 mg to 45 mg, M1 C_{max} also seemed to increase linearly with the dose after a single oral administration. When comparing different types of tumors in the study of II, there was no significant difference in ATG-008 or M1 competition.

Non-clinical pharmacological evaluation of M1 showed that the metabolite was about 1.5-2 times weaker than ATG-008 in inhibiting mTOR pathway. In vitro, the exposure of M1 metabolites to the inhibitory effect of mTOR pathway similar to ATG-008 in PC3 human prostate xenotransplantation model was about 5 times higher. In vitro metabolic studies showed that M1 was formed by O-demethylation. The preliminary competition results show that the terminal half-life of M1 is about 2-3 times longer than that of ATG-008. Pharmacodynamic markers of inhibitory effect of mTOR kinase include stimulation and non-stimulation of monocytes, B cells and T cells

TORC1 (pS6, p4EBP1) and TORC2 (pAKT) blood biomarkers were detected by stimulation analysis. The inhibitory effect of 30 and 45 mg QD dose cohort on TORC1 and TORC2 biomarkers at the peak plasma concentration was close to the maximum. The additive effect of M1 with a half- life of 2-3 times longer than ATG-008 can partly explain the persistent mTOR inhibition effect which is expected to last for 8-20 hours. The preclinical EC50 concentrations of these biomarkers were 19 ng/mL ATG-008 and 69 ng/mL ATG-008, respectively. The inhibitory effect of TORC1 evaluated by p4EBP1 analysis was also confirmed to be concentration-dependent, but its degree was weaker than that of TORC2 biomarkers. During the expansion period of the study, the inhibitory effects of blood TORC1 and TORC2 biomarkers were similar among all types of tumors.

Preclinical assessment confirmed that the benefit risk ratio of ATG-008 was acceptable as a whole, and antitumor activity signal could be observed in several types of tumors. Data from the first human I/II phase study suggest that the safety of ATG-008 is comparable to that of other mTOR pathway inhibitors.

For more details, see ATG-008 (CC-223) Manual of researchers。

1.3.2. Toripalimab Injection

Toripalimab Injection is a recombinant humanized anti-programmed cell death protein -1 (PD-1) monoclonal antibody injection. It was conditionally approved by the National Medical Products Administration in December 2018 for the treatment of locally advanced or metastatic melanoma that has progressed after prior standard therapy. Phase II clinical trial projects, of which 7 have been

completed and 20 are still under way. A total of about 1241 subjects received monotherapy or combined treatment with Toripalimab injection, of which 1109 cases received single drug treatment and 132 cases received combined treatment.

PD-1 is an inhibitory receptor of immunoglobulin family on the surface of activated T lymphocytes. Its ligands are B7 homologous protein programmed death ligand-1 (PD-L1) (also known as B7-H1) and programmed death ligand-2 (PD-L2) (also known as B7-DC). The binding of PD-1/PD-L1 plays an important role in down-regulating T cell activation and maintaining peripheral immune tolerance, so tumor cells inhibit T cell activation by expressing PD-L1 and then interact with PD-1 to avoid the killing of immune cells. The blocking of this immune checkpoint can enhance the proliferation, survival and killing activity of T cells to achieve the effect of tumor immunotherapy.

PD-1 and cytotoxicity T cell associated protein 4 (CTLA-4), which express similar structure, are two of the most important T lymphocytes immune checkpoint. Ipilimumab, a whole-person IgG1 monoclonal antibody drug targeting CTLA-4, was developed by Squibb and approved for sale in the United States in 2011. For the PD-1/PD-L1 pathway, by the end of 2017, FDA in the United States had approved five monoclonal antibody drugs, namely, Nivolumab, Pembrolizumab, Atezolizumab (PD-L1), Avelumab (PD-L1) and Durvalumab (PD-L1), for 11 indications. These include melanoma, non-small cell lung cancer, renal cell carcinoma, classical Hodgkin's lymphoma, head and neck cancer, bladder cancer (urothelial carcinoma), gastric cancer, Merkel cell skin cancer, solid tumor carrying MSI-H or dMMR and MSI-H or dMMR colorectal cancer. Based on the data of objective remission rate and duration of remission, Nivolumab and Pembrolizumab were also accelerated approved by FDA for HCC subjects who had previously used sorafenib.

PD-1 pathway blocking drugs have high efficiency and broad-spectrum antitumor characteristics. However, there is still room for improvement in clinical research on the types of tumors with weak immunogenicity and how to better control the side effects of drugs (such as pneumonia, dermatitis, colitis, hepatitis, nephritis, arthralgia, thyroid dysfunction and other adverse reactions). Compared with the foreign homologue drugs, Nivolumab injection has different antigenic determinant (CDR) and higher affinity, which can specifically bind to PD-1 and effectively block PD-1 and its ligands PD-L1 (B7-H1) and PD-L2(B7-DC) interaction to activate cytotoxicity T lymphocytes and inhibit tumor growth. Pre-clinical research data show that toripalimab, nivolumab and pembrolizumab have similar safety, but have better in vivo efficacy, so they have the best clinical antitumor effect of the same kind of drugs.

For more details, see Manual of researchers of toripalimab injection.

1.4. Research rationale

At present, a large number of clinical studies are being carried out in other types of cancer, as well as in combination with other treatments (including radiotherapy, chemotherapy, targeted therapy, tumor vaccine and other immunostimulatory therapy). The different antitumor activity mechanism of PD-1 pathway blocker and ATG-008 provides the possibility for tumor treatment.

Although PD-1 has shown good clinical effect on many kinds of tumors, including melanoma, renal cancer and lung cancer, when PD-1 is used as a single drug, some patients do not respond to PD-1 tumor immunotherapy, or some patients recur after treatment. Therefore, the combined treatment of tumor with PD-1 has become a means to improve the response rate and curative effect of tumor treatment. A number of preclinical studies have shown that the combination of mTOR inhibitor and PD-1/PD-L1 monoclonal antibody has a good effect on oral cancer, renal cell carcinoma and liver cancer. One study found that PD-1 can bind to eukaryotic activating factor 4e (eIF4E), ribosomal protein S6 (S6) downstream of the mTOR pathway, thus promoting the phosphorylation of these proteins. The phosphorylation of eukaryotic initiation factor 4e (eIF4E) increases the expression of Cyclin D1, which leads to the amplification of protein synthesis. Finally, it became the possible cause of HCC disease. If PD-1 and mTOR are blocked at the same time, the growth of liver cancer can be better inhibited.

Figure 1. PD-1/PD-L1 anti-tumor signal pathway

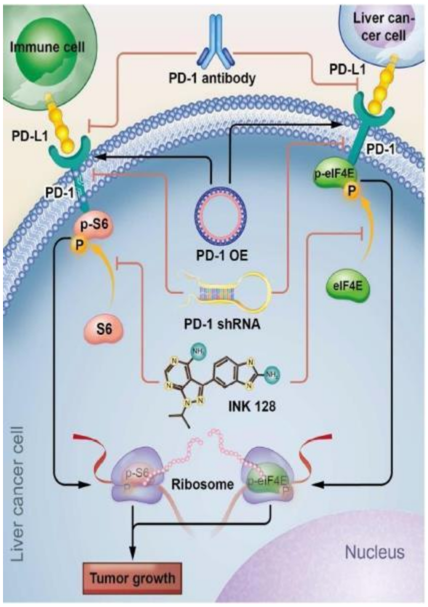

Literature (Li 2017) have been reported that the combination of PD-1 monoclonal antibody and mTOR inhibitor (INK-128) can produce significant synergistic effect in animal models. INK128 (mTOR inhibitor), PD-1 monoclonal antibody and mTOR inhibitor combined with PD-1 monoclonal antibody were given to NOD/SCID mice model of liver cancer. The results showed that PD-1 monoclonal antibody combined with mTOR inhibitor had stronger inhibitory activity than single drug experimental group.

Figure 2. In vivo antitumor activity of mTOR inhibitor combined with PD-1 monoclonal antibody

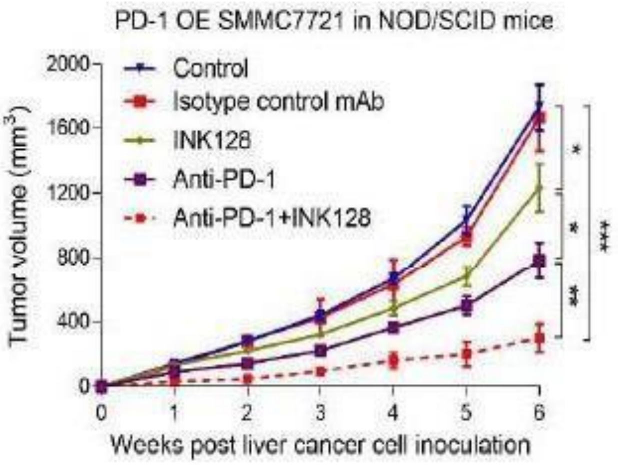

In addition, there are literature reports (Hirayama 2016), the combination of PD-L1 monoclonal antibody and mTOR inhibitor had a good effect on the animal model of renal cell carcinoma (RCC) before clinic. Everolimus is a kind of mTOR inhibitor. In evaluating the monoclonal antibodies of everolimus and PD-L1 in Balb/c mice with renal cell carcinoma, the mice were divided into four groups and treated with blank, PD-L1 monoclonal antibody and everolimus combined with PD-L1 monoclonal antibody for 2 weeks, respectively. There was no significant difference in animal weight, but the monoclonal antibody against everolimus combined with PD-L1 showed a stronger inhibitory effect on tumor than both drugs alone.

Figure 3. In vivo anti-renal cell carcinoma activity of mTOR inhibitor combined with PD-1 monoclonal antibody

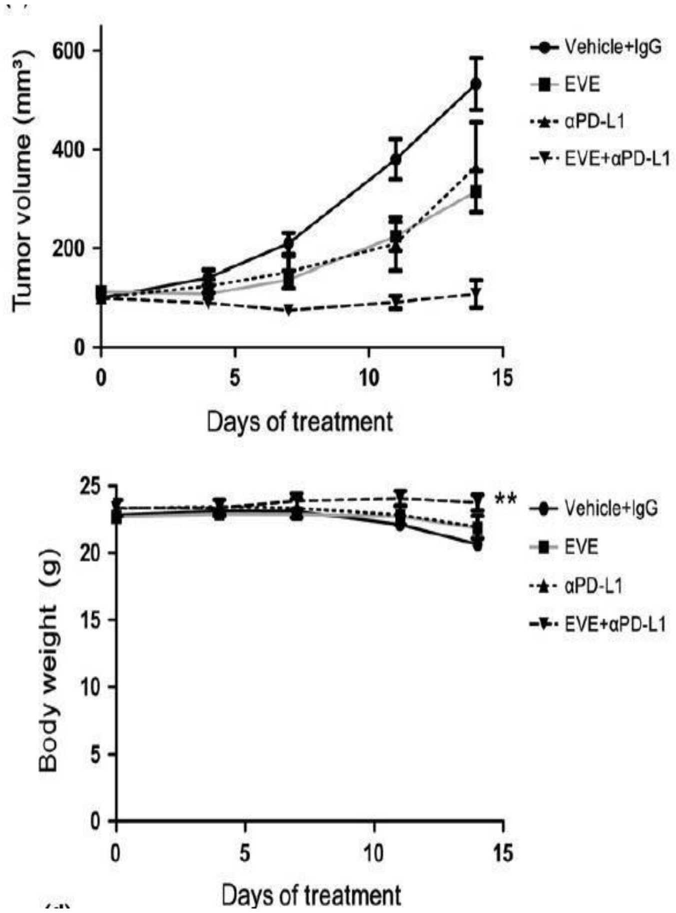

To sum up, the combination of mTOR inhibitor ATG-008 and anti-PD-1 monoclonal antibody Toripalimab will improve the effect of treating a variety of tumors and prolong the response time of anti-tumor compared with a single drug.

Clinical trial data for Toripalimab shows that as of December 16, 2019, out of 985 subjects, 964 (97.9%) experienced at least one adverse event, with 928 (94.2%) experiencing adverse events related to the study. The most commonly reported (≥10%) adverse events related to the study drug were anemia, alanine aminotransferase elevation, aspartate aminotransferase elevation, fever, cough, decreased white blood cell count, rash, decreased appetite, fatigue, hypothyroidism, elevated thyroid-stimulating hormone, pruritus, elevated blood glucose, decreased white blood cell count, and urinary protein detected. Of the participants, 161 (16.3%) experienced serious adverse events related to the study drug, with serious adverse events reported at a rate ≥1% being lung infections and abnormal liver function tests. 302 (30.7%) participants experienced Grade 3 or higher adverse events related to the study drug, with adverse events reported at a rate ≥1% being anemia, hyponatremia, lung infections, and abnormal liver function indicators. Immune-related adverse reactions were observed in 236 (31.8%) cases, with the most common being hypothyroidism, occurring at a rate of 13.6%. One participant died from interstitial pneumonia. Other immune-related adverse reactions included endocrine diseases, skin adverse reactions, colitis, nephritis, myocarditis, pancreatitis, hyperglycemia or diabetes, hepatitis, and adrenal insufficiency, most of which were Grades 1-2 and did not lead to trial drug discontinuation or interruption, with most completely resolving. These adverse reactions were consistent with immune-related adverse reactions observed with other similar products, with no new immune-related adverse reactions discovered.

ATG-008 has been studied in over 400 participants globally, including healthy subjects, and participants with advanced solid tumors and hematologic malignancies. Adverse drug reactions included hematologic and lymphatic system disorders (neutropenia, thrombocytopenia), gastrointestinal disorders (stomatitis, nausea, vomiting, diarrhea, pancreatitis), systemic disorders and administration site reactions (fatigue/weakness, fever, peripheral edema), infections (sepsis, infection, pneumonia), metabolic and nutritional disorders (decreased appetite, dehydration, hyperglycemia, hypokalemia, hyponatremia, hypophosphatemia, hypercholesterolemia, hypertriglyceridemia), renal and urinary system disorders (increased serum creatinine, renal dysfunction, acute renal failure, acute tubular necrosis), respiratory, thoracic, and mediastinal disorders (pulmonary tissue inflammation), and skin and subcutaneous tissue disorders (rash, acneiform dermatitis). No unexpected trends or clinically significant baseline findings were observed in hematology, chemistry, urinalysis, immunology, thyroid, or vital signs. Drug-related changes observed in laboratory measurements (including glucose, insulin, C-peptide, CD4+, and CD8+) were consistent with the expected mechanism of action based on mTOR pathway inhibition. The safety profile of ATG-008 is similar to other mTOR pathway inhibitors.

Overall, the safety profile of ATG-008 in healthy subjects is consistent with that in tumor subjects. AstraZeneca's mTOR1/2 inhibitor Vistusertib and PD-L1 monoclonal antibody Durvalumab have been studied in clinical trials, with no unforeseen adverse reactions reported.

In summary, the main adverse reactions of ATG-008 are metabolic in nature, while the main adverse reactions of Toripalimab are immune-related, with a low degree of overlap between the two. The likelihood of additive and unforeseen adverse reactions from the combination of ATG-008 and Toripalimab is low.

Potential Drug-drug Interactions:

In vitro studies evaluated the direct (competitive or non-competitive) and time-dependent inhibition of CYP enzymes by ATG-008, which may lead to potential drug-drug interactions. ATG-008 (up to 50 μM) exhibited no or minimal (50 μM for all isoforms (except CYP2C9). ATG-008 is a weak inhibitor of CYP2C9 and CYP2C19 (50% inhibition concentration [IC50]

≥27.4 μM), but not a time-dependent inhibitor of CYP enzymes. Pharmacokinetic studies of Toripalimab b showed that the metabolism of Toripalimab injection does not involve cytochrome P450 enzymes or conjugation/glucuronidation reactions. AstraZeneca's mTOR1/2 inhibitor Vistusertib and PD-L1 monoclonal antibody Durvalumab have been studied in clinical trials without significant drug interactions reported.

Therefore, it is anticipated that at planned therapeutic concentrations, the co-administration of ATG-008 and Toripalimab will not result in metabolic inhibition of these enzymes, leading to clinically relevant drug-drug interactions.

This study aims to evaluate the combination therapy of ATG-008 and Toripalimab, assessing the dose-limiting toxicity (DLT), safety, tolerability, and pharmacokinetic (PK) characteristics of the combination regimen in patients with advanced solid tumors, as well as evaluating the anti-tumor activity of ATG-008 combined with Toripalimab in patients with advanced solid tumors.

1.4.1. Dose Selection rationale

1.4.2. Dose Selection rationale of ATG-008

The maximum tolerated dose of ATG-008 established in preliminary trials was 45 mg per day. However, due to the higher frequency of dose reductions in the 45 mg per day group (study CC-223-ST-001), this dose was deemed unsuitable for further investigation in subjects with unresectable HCC. The daily dose of 30 mg demonstrated good tolerability in most subjects and approached maximal inhibition of mTOR biomarkers [pAKT, pS6, and phosphorylated 4E-binding protein 1 (P4EBP1)] in the blood, with inhibition of various markers ranging from at least 40% to 70%. The plasma exposure of ATG-008 at a dose of 30 mg exceeded the levels required for mTOR biomarker inhibition and anti-tumor activity in preclinical tumor xenograft models. Despite dose reductions, encouraging signals of activity were observed in the HCC cohort, including three subjects meeting the criteria for partial response (PR) on imaging assessments (target lesion reduction>30%) after two treatment cycles, decreased uptake on positron emission tomography (PET) imaging, and reduced alpha-fetoprotein (AFP) levels. All three HCC subjects who achieved PR on imaging assessments in Phase 2 studies required dose reduction to 30 mg. Therefore, based on these data, the dose escalation phase of this study selected a maximum daily dose of 30 mg.

1.4.3. Dose Selection Basis for Toripalimab

This study will utilize a fixed dose of 240 mg of Toripalimab administered every 3 weeks. Based on data from three Phase I dose-escalation clinical studies (CT1, CT2, CT3) encompassing various dose groups of Toripalimab (doses ranging from 0.3 to 10 mg/kg administered every 2 weeks, as well as a fixed dose of 240 mg), summary pharmacokinetic (PK) analysis showed that after intravenous infusion of Toripalimab at doses of 1, 3, and 10 mg/kg, the drug exhibited predominantly linear PK characteristics; however, at doses increased to 10 mg/kg, some degree of non-linear PK characteristics was observed. This demonstrates the apparent "long-acting" feature of Toripalimab as an antibody-like drug. Continuous intravenous administration of Toripalimab for approximately 3-4 doses resulted in attainment of steady-state blood concentrations, with a mean half-life of approximately 222.98 ± 61.92 hours (9.29 ± 2.58 days), C_{max} values of 77.17 ± 47.3, and AUC_{0-t} values of 11635.89 ± 7204.64 in the 3 mg/kg group (n=37) after multiple administrations.

The steady-state minimum blood concentration of 3 mg/kg administered every two weeks was approximately in the range of 20-40 μg/mL. In vitro experiments indicated that Toripalimab concentrations > 20 nM or 3 μg/mL saturated the PD-1 receptor on T cells, and considering the limited penetration of large antibody molecules into the tumor microenvironment, maintaining peripheral blood Toripalimab levels at 25 μg/mL was suggested to ensure complete PD-1 occupancy on lymphocytes in the tumor microenvironment. Phase I studies demonstrated that various dose groups (0.3, 1, 3, 10 mg/kg, administered every 2 weeks) could maintain complete PD-1 receptor occupancy throughout the treatment period. Based on the aforementioned PK study results and receptor

occupancy data, a dose of 3 mg/kg administered every 2 weeks was selected as the dose for the pivotal Phase II study (CT4). Additionally, exploratory studies (CT5 and CT7) investigated a fixed dose of 360 mg administered every 3 weeks, which, based on preliminary PK data from 9 subjects, resulted in a 153% increase in steady-state peak concentration (166.53 µg/mL: 95%CI 121.0 to 212.0 vs 108.83 µg/mL: 95% CI 87.5 to 130.0), a 133%
increase in steady-state peak concentration (47.92 µg/mL: 95% CI 31.8 to 64.0 vs 36.09 µg/mL: 95% CI 27.7 to 44.5), and a 138% increase in AUC_{0-85} (164993 µg·hr /mL vs 119814 µg·hr /mL) compared to 3 mg/kg administered every 2 weeks (from CT1, 11 subjects). PK modeling predicted that the trough concentration of 240 mg administered every 3 weeks (~32 µg/mL) and drug exposure were similar to those of 3 mg/kg administered every 2 weeks. Considering the comprehensive results of Toripalimab PK, receptor occupancy, and pivotal Phase II melanoma studies, support was provided for a fixed dose of 240 mg of Toripalimab administered every 3 weeks as the recommended dose for late-stage clinical studies.

2. Objective and Endpoints

Objective and Endpoints：

Dose Escalation Phase	
**Primary Objectives**	**Primary Endpoints**
To evaluate the safety and tolerability of ATG-008 combined with Toripalimab in subjects with advanced solid tumors	To establish MTD and RP2D of ATG-008 combined with Toripalimab<br>To evaluate the safety and tolerability of ATG-008 combined with Toripalimab
**Secondary Objectives**	**Secondary Endpoints**
To evaluate the pharmacokinetic characteristics of ATG-008 combined with Toripalimab	Pharmacokinetic parameters of ATG-008 for ATG-008 combined with Toripalimab
To evaluate the efficacy of ATG-008 combined with Toripalimab in subjects with advanced solid tumors	Evaluated by investigators according to RECIST 1.1：<br>• ORR<br>• DOR<br>• DCR<br>PFS<br>OS
To evaluate the immunogenicity of Toripalimab	Incidence rate of ADA
**Exploratory Objectives**	**Exploratory Endpoints**
To evaluate the efficacy of ATG-008 combined with Toripalimab in subjects with advanced solid tumors according to iRECIST 1.1 criteria	Evaluated by investigators according to iRECIST:<br>ORR, DOR, DCR, and PFS
To explore potential biomarkers in blood and tumor tissues	Levels of MSI, TMB and PD-L1 (tumor tissues), or sPD-L1(blood samples), etc.
**Dose Expansion Phase**	
**Primary Objectives**	**Primary Endpoints**
To evaluate the efficacy of ATG-008 combined with Toripalimab in subjects with advanced solid tumors	ORR evaluated by investigators according to RECIST 1.1 or RANO criteria (GBM subjects only)
**Secondary Objective**	**Secondary Endpoints**
To further evaluate the efficiency of treatment with ATG-	Evaluated by investigators according to RECIST

008 combined with Toripalimab in subjects with advanced solid tumors	1.1<br>• DOR<br>• DCR<br>• PFS<br>OS
To further evaluate the safety and tolerability of ATG-008 combined with Toripalimabin subjects with advanced solid tumors	Safety and tolerability of ATG-008 combined with Toripalimab
To further evaluate the immunogenicity of Toripalimab	Incidence rate of ADA
**Exploratory Objectives**	**Exploratory Endpoints**
To evaluate the efficacy of ATG-008 combined with Toripalimab in subjects with advanced solid tumors according to iRECIST and iRANO criteria (GBM subjects only)	Evaluated by investigators according to iRECIST and iRANO criteria (GBM subjects only)<br>• ORR、DOR、DCR and PFS
To explore the potential biomarkers in blood and tumor tissues	• Levels of MSI, TMB and PD-L1 (tumor tissues), or sPD-L1(blood samples),etc.<br>Changes of CA125(ovarian cancers only)

Abbreviations: ADA = Antidrug Antibody; CA125= carbohydrate antigen 125;DCR = Disease Control Rate; DOR = Duration of Response; GBM = Glioblastoma; HCC = Hepatocellular Carcinoma; iRANO = Immunotherapy Response Assessment in Neuro-oncology; iRECIST = Evaluation Criteria for Immune Efficacy of Solid Tumors; MSI = Microsatellite Instability; MTD = Maximum Tolerated Dose; ORR = Overall Response Rate; OS = Overall Survival; PFS = Progression Free Survival; PK = Pharmacokinetics; RANO = Response Assessment In Neuro-Oncology; RECIST = Efficacy Evaluation Criteria For Solid Tumors; RP2D = Recommended Phase II Dose; Spd-L1 = Serum Free Programmed Death Ligand-1;TMB= Tumor Mutational Burden.

3. Study Design

3.1 Overall Design

This is an open-label study, including two phases: Dose Escalation Phase and Dose Expansion Phase. The safety and tolerability of ATG-008 combined with Toripalimab, as well as DLT evaluation, maximum tolerated dose (MTD), and recommended phase II dose (RP2D) in advanced solid tumor subjects will be evaluated in the Dose Escalation Phase. Once RP2D is determined, Dose Expansion study will be conducted at the RP2D dose level in subjects with different types of solid tumors (including HCC, NET, advanced or metastatic gynecologic tumors, GBM, etc.) to further evaluate the anti-tumor efficacy and safety of ATG 008 combined with Toripalimab.

ATG-008 will be administered orally once daily (QD), with a provisional maximum dose of 30 mg QD at present. Toripalimab will be administered intravenously as a fixed dose of 240 mg, once every 3 weeks (Q3W). The longest duration of Toripalimab is not more than 2 years. The investigational dosage as following

Dose level	The combined treatment dose of ATG-008+Toripalimab	
	**Toripalimab dose**	**ATG-008 dose**
1	240 mg Q3W	15 mg QD
2	240 mg Q3W	20 mg QD
3	240 mg Q3W	30 mg QD

Q3W= Once every 3 Weeks; QD= Once a Day

When using the principles of the standard “3+3” design, for each dose level, the

administration of the first dose of the study drug will be at least 72 hours apart between the first 2 subjects. At least 3 subjects will be enrolled for DLT evaluation in each dose level. If more than 3 subjects are eligible, additional subjects will be allowed to enroll (up to a total of 6 subjects).

- At a given dose level, if no DLT occurs in 3 subjects, the study will proceed to the next higher dose level, enrolling 3 new subjects for further evaluation.
- At a given dose level, if 1 out of 3 subjects experiences a DLT, 3 new subjects will be enrolled at the current dose level. If none of the newly enrolled subjects experiences a DLT, escalation to the next higher dose level will occur, enrolling 3 new subjects for further evaluation.
- At a given dose level, if ≥2 subjects experience a DLT during the DLT assessment period, that dose level will be defined as the DLT dose. No new subjects will be enrolled at this dose level, and dose escalation will be halted. A maximum of 6 evaluable cases may be enrolled at the DLT dose.
- The MTD is defined as the dose level at which ≥2 subjects experience a DLT; the preceding dose is then considered the MTD.
- The RP2D will be determined based on safety, PK data, and preliminary efficacy analysis.

DLT evaluation will occur within the first cycle (1-21 days) among evaluable subjects. Evaluable subjects are defined as those who experience a DLT during Cycle 1 treatment, or those who do not experience a DLT but receive at least 80% of the planned dose of ATG-008 and at least 80% of the dose of toripalimab, and complete all safety assessments required for Cycle 1. If the number of evaluable subjects is less than the required number specified in the protocol, additional subjects will be enrolled to reach the minimum required number of evaluable cases.

When a DLT occurs, the study center must immediately notify the drug provider. If 2 DLTs occur at the same dose level, enrollment at the current or higher dose level will be paused until a dose escalation meeting evaluates and determines whether the dose level should be defined as the DLT dose. Safety, PK, and efficacy data monitoring will continue throughout the dose escalation phase. A Safety Monitoring Committee (SMC), consisting of investigators and medical representatives from the drug provider, will be established to evaluate safety and make dose escalation decisions, including modifying dose escalation based on DLT occurrence or determining the RP2D. Once the RP2D is determined, subjects in lower dose groups may escalate to the RP2D dose level. (Please find the details of SMC decision)

Based on safety and tolerability data from CC-223-ST-001-B Phase I solid tumor subjects, the average dose for the NET group of 47 subjects is approximately 20 mg. Therefore, during dose escalation, if the ATG-008 20 mg QD dose group (ATG-008 combined with toripalimab treatment) demonstrates good safety and tolerability at the end of enrollment, NET cohort subjects will undergo dose expansion at the ATG-008 20 mg QD dose combined with toripalimab, while doses expansion for other cohorts will be determined after the completion of the dose escalation study (30 mg dose group) and discussed by the SMC.

During the dose expansion phase, subjects with different types of solid tumors will be enrolled at the RP2D level, including the following 5 cohorts: advanced HCC, NET, advanced or metastatic gynecological tumors (endometrial cancer, cervical cancer, and ovarian cancer), GBM, and advanced solid tumor subjects (approximately 10-12 subjects per cohort), to further evaluate the preliminary efficacy, safety, and tolerability of the study drug at this dose level. If the ORR for the GBM cohort is approximately >10% or for other tumor types or cohorts is approximately >20%, the number of subjects enrolled in the corresponding tumor type or cohort may increase to approximately 40 subjects, with a total enrollment limit of 150 subjects for the study.

During this period, if more than one-third of all subjects experience a DLT within the first cycle, enrollment will be suspended, and an immediate SMC meeting will be convened to discuss the safety of that dose level. The SMC will also decide whether to recruit additional subjects or conduct other dose group study for further investigation.

3.1.1. Definition of DLT

All toxicities or adverse events are graded according to the National Cancer Institute Common Terminology Criteria for Adverse Events (NCI-CTCAE) version 5.0. Any of the following toxicities occurring within the first cycle (21 days) will be considered DLTs if judged by the investigator to be related to the study drug rather than the disease or disease- related processes:

Hematologic toxicities:

Grade 4 neutropenia lasting >7 days
Febrile neutropenia of grade 3/4
Grade 4 thrombocytopenia or grade 3 thrombocytopenia with clinically significant bleeding

Non-hematologic toxicities:

Toxicities of grade ≥3, except for:

- Laboratory abnormalities, diarrhea, nausea, and vomiting improving to grade ≤2 within 3 days after optimal medical treatment
- Fatigue of grade 3 lasting ≤7 days
- Acne-like or maculopapular rash improving to grade ≤2 within 7 days after optimal medical treatment
- Grade 3 infusion-related reactions resolving within 6 hours after appropriate clinical management
- Hair loss

3.2. Basic Rationale of Study Design

ATG-008 is a potent, selective inhibitor of mammalian target of rapamycin (mTOR) in development, acting as a dual inhibitor of mTORC1 and mTORC2 complexes. Toripalimab is a recombinant humanized (97% human) anti-PD-1 monoclonal antibody. Preclinical studies have shown that combining mTORC1/2 inhibition with immune checkpoint inhibition enhances anti-tumor activity across various tumor types. Therefore, this study aims to evaluate the DLTs, safety, tolerability, and PK characteristics of ATG-008 combined with toripalimab therapy in advanced solid tumor subjects, as well as the efficacy of this combination therapy in advanced solid tumor subjects.

3.3. Study Duration

All subjects will continue to receive combination therapy with ATG-008 and toripalimab, with the treatment duration of toripalimab not exceeding 2 years, until confirmed radiographic disease progression, intolerable toxicity, subject or investigator decision to discontinue, subject loss to follow-up, or death, whichever occurs first.

Study termination is defined as 12 months after the last subject is enrolled, withdrawal of consent, death, or loss to follow-up (whichever occurs first). Additionally, the drug provider may decide to terminate the study at any time for reasons including but not limited to safety concerns.

After disease progression or the end of this study, if subjects require continued treatment and based on subject's willingness, the investigator may determine the suitability for continued dosing and, with the agreement of the drug provider, may continue to provide ATG-008 and/or toripalimab to the subject (up to a maximum of 2 years), and continue to collect safety data through voluntary reporting. ATG-008 and/or toripalimab may be provided through additional expanded studies or other forms at the discretion of the drug provider.

4. Study Population

4.1. Number of subjects

The target population in the dose escalation phase of this study was participants with advanced solid tumors (approximately 3-18 cases). In the dose-expansion phase, a cohort of subjects with different types of solid tumors will be enrolled at the RP2D level, including the following five cohorts (approximately 50-150 patients) : advanced HCC, NET, advanced or metastatic gynecologic tumors (endometrial cancer, cervical cancer, and ovarian cancer), GBM, and advanced solid tumors (approximately 10-12 patients per cohort) to further evaluate the preliminary efficacy, safety, and tolerability of the study drug at this dose level. If the ORR for the GBM cohort is approximately >10% or for other tumor types or cohorts is approximately >20%, the number of subjects enrolled in the corresponding tumor type or cohort may increase to approximately 40 subjects, with a total enrollment limit of 150 subjects for the study. The number of subjects recruited in each cohort is primarily based on clinical considerations, without statistical hypothesis testing.

4.2. Inclusion criteria:

Subjects must meet all the following inclusion criteria to be eligible for this study:

1. Know and voluntarily sign informed consent.
2. 18-70 years old (including 18 and 70 years old), weight ≥45 Kg (Dose escalation only).
3. At least one measurable lesion according to RECIST 1.1 or RANO criteria; lesions not recommended for target within the radiation field (except for GBM).
4. ECOG performance status score 0 or 1.
5. Clinical chemistry test results must meet the following:
   a) Aspartate aminotransferase (AST) and alanine aminotransferase (ALT) ≤2.5 ×normal upper limit (ULN)
   b) Total bilirubin ≤1.5 × ULN
   c) Serum albumin >29 g/L
   d) Creatinine ≤1.5 × ULN or 24-hour serum creatinine clearance ≥50 mL/min
   e) Lipase and amylase ≤2 × ULN.
6. Adequate bone marrow function and meet the following criteria:
   a) Absolute neutrophil count (ANC) ≥1.5 × 10^9 cells/L
   b) Platelet count ≥75 × 10^9 cells/L
   c) Hemoglobin ≥90 g/L.
7. Coagulation function: International Normalized Ratio (INR) ≤ 2.0, Prothrombin Time (PT) ≤ 1.5 × ULN.
8. The hepatitis B virus/hepatitis C virus (HBV/HCV) test must meet the following criteria:
   a. For non-HCC subjects: Subjects with positive hepatitis B surface antigens (HBsAg) or HCV antibody, must further pass quantitative detection of HBV-DNA (no more than 2500 copies/mL or 500 IU/mL) and HCV-RNA (not exceed the lower limit of detection), and will be enrolled after ruling out the active hepatitis B or hepatitis C infection which need to receive treatment. Hepatitis B virus carriers, hepatitis B stable subjects after drug treatment (DNA quantitative detection should not be higher than 2500 copies/mL or 500 IU/mL) and cured hepatitis C subjects can be included in the study.
   b. For HCC subjects: Subjects with HBsAg positive and/or hepatitis B core antibody

positive, meeting the following conditions can be included: HBV-DNA must < 2000 IU/mL or 10^4 copies/mL when enrollment, if higher than this, subjects must receive antiviral treatment at first to let HBV-DNA drop to the normal range for at least 2 weeks, and must continue to receive antiviral treatment during the study treatment. For subjects who have received antiviral therapy for hepatitis B in the past and have normal range of HBV-DNA when enrollment, antiviral therapy must be received throughout the study treatment. HCV-RNA positive subjects with normal liver function may also be enrolled in this study but must receive approved standard anti-HCV therapy.

9. Except for hearing loss and alopecia, all toxicity caused by previous anti-tumor therapy must have recovered to ≤ Grade 1 (according to NCI-CTCAE version 5.0).
10. Life expectancy is longer than 3 months.
11. Fertile men and women of childbearing age must agree to use effective contraceptives from they sign the informed consent to180 days after the last dose of study drug. Women of childbearing age include premenopausal women and women within 2 years after menopause. Women of childbearing age must have a negative blood pregnancy test at screening.

Inclusion criteria should also be met in Dose Escalation Phase：

1. Subjects are histopathologically confirmed with advanced, relapsed or refractory solid tumors (including but not limited to breast cancer, neuroendocrine cancer, lung cancer, cholangiocarcinoma, gastroesophageal junction adenocarcinoma and HCC). Subjects with HCC will meet the inclusion/exclusion criteria of the Dose Expansion Phase. No standard treatment available, intolerant or reject to standard treatment.

Inclusion criteria should also be met in Dose Expansion Phase：

1. Meets all the criteria for any of the following cohort

Advanced HCC Cohort:

a. Pathologically or clinically confirmed HCC, following Guidelines for Diagnosis and Treatment of Primary Liver Cancer in China (2018 Edition)

b. Unresectable Stage B (intermediate stage) or Stage C (advanced stage) HCC according to Barcelona Clinic Liver Cancer Staging (BCLC). Subjects with stage B must have progressed after surgery /local treatment, or be unsuitable for surgery/local treatment.

c. Previously received at least one type of systemic therapy for HCC (including sorafenib, oxaliplatin-based chemotherapy, lenvatinib, or regorafenib, etc.).

d. Child-Pugh A or Child-Pugh B7 without encephalopathy

Advanced NET Cohort:

a. Histologically confirmed, unresectable advanced local or metastatic non-functional neuroendocrine neoplasms, including well-differentiated neuroendocrine neoplasms and poorly differentiated neuroendocrine carcinomas;

b. Subjects with poorly differentiated neuroendocrine carcinoma must have previously received platinum-based chemotherapy. Subjects with well-differentiated neuroendocrine tumors must have received at least one systemic treatment, including somatostatin analogues, antiangiogenic agents, chemotherapy, etc.

Advanced GBM Cohort:

a. Histologically confirmed GBM (WHO Grade IV);

b. Radiologically confirmed recurrence or progression after prior radiotherapy and temozolomide treatment;

c. Karnofsky Functional Status score (KPS) ≥70;

d. Prior to screening, antiepileptic drug therapy should be stable for at least 4 weeks or more

Advanced or Metastatic Gynecologic Tumors Cohort:

a. Histologically confirmed, recurrent or metastatic gynecological tumors, any of the following:

i. Ovarian cancer is epithelial ovarian, fallopian tube or peritoneal cancer, includes high-grade serous, endometrioid and clear cell carcinoma;

ii. Cervical cancer is advanced, unresectable, unsuitable for radiotherapy and/or metastatic cervical cancer (squamous cell carcinoma, adenocarcinoma, adenosquamous carcinoma);

iii. Advanced/recurrent endometrioidadenocarcinoma;

b. Progression or recurrence after previous chemotherapy containing platinum and/or taxol.

Advanced solid tumor cohort:

a. Subjects with histologically confirmed advanced, recurrent or refractory solid tumor that cannot be resected (recommended but not limited to soft-tissue sarcoma, papillary thyroid carcinoma [Thyroid Stimulating Hormone (TSH) level <0.5 mU/L], and cholangiocarcinoma).

2. No standard treatment available, intolerant or reject to standard treatment

4.3. Exclusion criteria:

Subjects meeting any of the following criteria are not allowed to be enrolled in the study:

1. Have ahistory of hepatic encephalopathy.
2. Have a history of organ transplantation (eg., liver transplantation).
3. Stable period determined by imaging <3 months, or symptomatic brain metastases (not applicable for GBM).
4. Have a thyroid disorder with a clinically significant thyroid dysfunction judged by the investigator (not applicable for thyroid cancer is in Dose Expansion Phase).
5. Active or history of upper gastrointestinal bleeding, ulcers, or esophageal varices with bleeding within 6 months.
6. Subjects with a history of human immunodeficiency virus (HIV) infection and/or acquired immunodeficiency syndrome.
7. Major surgery has been performed within 4 weeks prior to the first dose or is expected to be performed during the study period.
8. Have received systemic chemotherapy, radiotherapy, traditional Chinese medicine with anti-tumor activity, or local treatment for tumor (including but not limited to radiofrequency ablation, hepatic arterial chemoembolization, and high-intensity focused ultrasound) within 4 weeks prior to the first dose. Immunotherapy (including anti-PD-1, anti-PD-L1, cytotoxic T cell-associated protein 4 [CTLA-4] or CAR-T cell therapy, etc.) within 6 weeks prior to the first dose; Prior immunotherapy with less than 2 full cycles and progression or clinical judgment as hyperprogression of immunotherapy.
9. Received live attenuated vaccine within 4 weeks prior to the first study dose (Subjects should not receive live attenuated vaccine at the time of study drug administration and up to 28 days after the last dose if enrolled).
10. Poorly controlled pleural or pericardial effusion (with clinical symptoms, fluctuating fluid or requiring repeated drainage, oral diuretics, etc.) at Screening. Ascites can be detected during the physical examination at Screening, or clinical symptoms caused by ascites, or require special treatment, such as repeated drainage, intraperitoneal drug perfusion, etc. (But subjects with ascites could be considered applicable for enrollment if their ascites can only be found through image examination).
11. Other primary malignancies occurred within 5 years prior to the first study dose except for locally curable malignancies (eg., basal or squamous cell skin cancer, superficial bladder cancer, prostate, cervical or breast carcinoma in situ, etc.).

12. Suffering from active or previously had autoimmune diseases with recurring potential (eg., systemic lupus erythematosus, rheumatoid arthritis, inflammatory bowel disease, autoimmune thyroid disease, vasculitis, psoriasis disease, etc.), or at risk of such diseases.
13. Immunosuppressive drugs are systemically used currently or within 14 days prior to the first dose. Except following:
    a. Intranasal, inhaled, topical steroids, or topical steroid injections (such as intra-articular injections)
    b. Systemic corticosteroid treatment not exceeding 10 mg/day prednisone or equivalent physiological dose
    c. Steroids as a preventive medication for allergic reactions (such as pretreatment before CT scan).
14. Suffer a persistent or active infection, a history of pneumonia due to immunotherapy, or currently have Grade 2 above pneumonia, or a history of active pulmonary tuberculosis infection within 1 year prior to the first dose (Subject who has a history of active pulmonary tuberculosis infection more than 1 year ago, and currently has no evidence of active tuberculosis judged by investigator could be applicable for enrollment).
15. Have clinically significant cardiovascular diseases, such as Class II or above cardiac dysfunction(NYHA Functional Classification), ischemic heart disease (such asmyocardial infarction or unstable angina pectoris), clinically significant supraventricular or ventricular arrhythmias, poorly controlled hypertension (systolic blood pressure> 150 mmHg and/or diastolic blood pressure> 100 mmHg), or echocardiogram showing ejection fraction <50%, or QT corrected interval by using the heart rate (QTc) > 450 msec (male), 470 msec (female).
16. Any serious gastrointestinal disease that may affect the absorption of the study drug
17. Previously received received mTOR (TORC1 and/or TORC2) inhibitors and/or PI3K/AKT/mTOR inhibitors.
18. Using/eating drugs or food known to have strong CYP3A4 inhibitory effects within 2 weeks prior to screening, including but not limited to atazanavir, clarithromycin, Indinavir, itraconazole, ketoconazole, nefazodone, nelfinavir, ritonavir, saquinavir, telithromycin, vinegar oleandomycin, voriconazole, grapefruit or grapefruit juice.
19. Receiving drugs known to have strong CYP3A4 induction within 2 weeks prior to screening, including but not limited to carbamazepine, phenobarbital, phenytoin, rifabutin, rifampicin, and hyperforin perforatum.
20. Receiving drugs as CYP3A4 substrates (with narrow therapeutic index) within 2 weeks prior to screening, including but not limited to dihydroergodemine, ergodemine, pimozide, astimidazole, cisapride, and terfenadine.
21. Have a history of severe allergic reactions to chimeric, human or humanized antibodies, or fusion proteins; Known to have hypersensitivity reactions to any components produced from CHO cells, or a Toripalimab monoclonal antibody (such as citric acid monohydrate, sodium citrate dihydrate, mannitol, polysorbate).
22. Investigator considers that the complications or other situations of the subject may affect compliance with the protocol or is not suitable for participation in this study.

Exclusion criteria should also be met in Dose Escalation Phase as following:

1. Subjects with diabetes or glycated hemoglobin (HbA1c)> 7%.

Exclusion criteria should also be met in Dose Expansion Phase as following:

1. Poorly controlled diabetes (HbA1c >7%)
2. For HCC subjects:
   a. Histopathological diagnosis is fibrous lamellar HCC, sarcomatoid HCC, bile duct cell carcinoma, or mixed liver cancer;
   b. Have received molecular targeted therapy, such as sorafenib, orlenvatinib within 4 weeks or 5 half-lives prior to the first dose;
   c. Have received palliative surgery in the liver area, within 4 weeks prior to the first dose.
   d. Imaging findings of major portal vein cancer metastasis
3. For GBM subjects: those who have received carmustine wafer implantation or intracranial radioactive implantation

5. Registration and enrollment

5.1. Screening and registration

All subjects will be screened to evaluate their research qualifications. The screening evaluation must be completed within 28 days before the first administration, with the exception of the following marked tests (such as laboratory tests). All screening assessments must be conducted before admission (first medication) and subsequent treatment, and the screening period assessment can be conducted at the same visit according to the timing of the scheme.

Drug providers will review the eligibility of all participants in this study. After all the screening procedures have been completed, the drug provider will check the eligibility of the subjects for admission, and then the subjects will be able to join the group.

The research center will send the drug provider by fax or e-mail to the group qualification checklist / subject registration form, which will be evaluated by the drug provider. After confirming the eligibility of the subjects, the drug provider returns the signed qualification checklist / subject registration form to the research center by fax or e-mail.

All laboratory analyses will be carried out in the local laboratory (unless otherwise indicated). Some of the laboratory numerical criteria must be screened within 7 days before the first dose of the drug is given and the subjects must be verified to enter the group. If necessary, the screening evaluation can be repeated in the screening time window, and the latest test results should be used to confirm the eligibility of the subjects. If, prior to the signing of the informed consent form (ICF), the daily clinical assessment tests are also in line with the assessment tests required for the screening period of the study, these assessments need not be repeated during the screening period.

During the competitive assessment and fasting blood biochemical examination, the subjects should be fasting for at least 6 hours before the visit.

5.2. The ID of the study subjects

Each subject will be assigned an ID when screened and taken into the group to establish an association with the data. Under no circumstances shall the subject number be reallocated or reused. Drug providers will identify subjects only by assignment number, initials, date of birth and sex. The researchers must maintain a main log of the subjects.

5.3. Screening failure

If the subject has signed ICF, but the drug provider does not confirm his eligibility to enter the group or does not start using the drug, the screening is considered a failure. For all

screening losers, the researchers will enter at least the subject number, demographic characteristics, and reasons for screening failure in the electronic case report form (eCRF). The data will also be stored in the researchers' research documents, and the research center can print the data in log format at the end of the study. Screening losers can be screened again after discussion with drug providers (only once). However, you need to reassign a number and indicate it as re-filtering.

6. Research procedure

For detailed research procedures, see the Research Process Table (Table 1).

6.1. Screening visit (28 days before the first dose administration)

All subjects will be screened to evaluate their research qualifications. The screening evaluation must be completed within 28 days before the first dose administration, with the exception of the tests indicated below (such as laboratory tests). All screening assessments must be conducted before admission (first medication) and subsequent treatment, and the screening period assessment can be conducted at the same visit according to the timing of the scheme.

If anyone have any questions about the eligibility of the subjects, please let the drug provider or the designated person know. During the implementation of this experiment, the contents of the research required by the test scheme shall not be exempted without authorization.

All laboratory analyses will be carried out in the local laboratory (unless otherwise indicated). Some of the laboratory numerical criteria must be screened within 7 days before the first dose of the study drug is given and the entry criteria of the subjects must be verified. If necessary, the screening evaluation can be repeated in the screening time window, and the latest test results should be used to confirm the eligibility of the subjects. If, prior to the signing of the ICF, the daily clinical assessment tests are also those required for the screening period of the study (for example, samples that need to be collected and sent to the central laboratory), these assessments need not be repeated during the screening period.

Subjects were required to perform competitive assessment and fasting chemical examination for at least 6 hours before the visit. The following actions will be done during filtering:

- Obtain informed consent (record the age of the day the ICF was signed), and the subject must sign on ICF before the screening process begins.
- Verify inclusion criteria and exclusion criteria.
- Record demographic data (date of birth, sex and nationality).
- Ask for a complete history of cancer, including specific information about diagnosis, staging, and histology, as well as other meaningful medical conditions.
- Previous cancer treatment: includes surgery, radiotherapy, systemic and local or any other treatment (such as hormones) for the subject's cancer (also record the causes of previous treatment discontinuation, such as disease progress, intolerance, or others).
- Evaluation of past and accompanying drug use.
- Measure height and weight.
- Complete physical examination (including general condition, skin, head, neck, ear, nose, mouth, throat, respiratory/lung, cardiovascular, gastrointestinal/abdominal, reproductive urethra, neurology, limbs and lymphoid system, as well as any other items that may be relevant).
- Vital signs (blood pressure, pulse, respiratory rate and body temperature) were measured.
- ECOG physical fitness assessment.
- Child-Pugh score was evaluated (only HCC subjects).
- Blood routine examination: hemoglobin, hematocrit, mean red blood cell volume, average erythrocyte hemoglobin content, average erythrocyte hemoglobin concentration, white blood cell count, white blood cell classification count, red blood cell count,

lymphocytes, monocytes, neutrophils, eosinophils, basophilic granulocytes and platelets. Reticulocytes need to be determined only when there are clinical indications.

● Fasting blood biochemical tests included sodium, potassium, chlorine, bicarbonate, blood urea nitrogen, urea, creatine, glucose, calcium, phosphate, magnesium, ALT, AST, alkaline phosphatase, total bilirubin, lactic dehydrogenase, total protein, albumin, amylase, lipase, creatine kinase and uric acid.

● Routine urine examination: including urine bilirubin, urine occult blood, urine red blood cells, urine ketone, pH value, urine protein, urine specific gravity and urobilinogen, etc., will be examined under microscope when there are clinical indications.

● Coagulation function test: PT, INR, partial thromboplastin time (PTT).

● Thyroid function test: thyrotropin (TSH), free triiodothyronine (T3), free tetraiodothyronine (T4).

● HbA1c detection.

● AFP quantitative detection (only HCC subjects).

● Quantitative detection of CA125 (ovarian cancer only).

● HBV/HCV/HIV detection: hepatitis B virus (HBV), hepatitis C virus (HCV) (including hepatitis B surface antigen [HBsAg], hepatitis B surface antibody [HBsAb], hepatitis B e antigen [HBeAg], hepatitis B e antibody [HBeAb], hepatitis B core antibody [HBcAb] and hepatitis C virus antibody [HCVAb]) and human immunodeficiency virus (HIV) serological examination.

● Antinuclear antibody (ANA) was detected.

● All female subjects of childbearing age required serum β-hCG pregnancy tests to be performed at the time of screening.

● Echocardiography.

● 12-lead ECG: single local evaluation When the 12-lead ECG is performed at the same time as other assessments, the 12-lead ECG check should always be performed first.

● Myocardial zymogram (including serum creatine kinase and serum creatine kinase isoenzyme).

● Tumor tissues were collected for detection of MSI, Tumor Mutational Burden (TMB) and PD- L1, as well as drug-related pathway mutations (including, but not limited to, PI3K, AKT, KEAP1, NFE2L2, STK11), or about 10 mL blood samples were collected as needed for serum free programmed death ligand-1(sPD-L1) detection.

● Treatment response assessment / tumor assessment: solid tumors (except GBM) were evaluated according to RECIST and iRECIST, and GBM was evaluated according to RANO (Appendix 9) And iRANO (Appendix 10). A tumor baseline assessment should be carried out not more than 28 days before administration and, preferably, as close to the beginning of administration or as soon as possible. At the time of screening, if a chest, abdominal and pelvic tumor scan (CT/MRI), as well as skull MRI, had been performed less than 28 days before the first dose of the drug was given, it would not need to be repeated. Subjects with bone lesions should undergo a confirmed bone scan at the base line.

● The combined use of drugs accepted after the signing of ICF and the adverse events that occurred (only serious adverse events need to be recorded before administration) are recorded.

6.2. Treatment Period (Combination Therapy with ATG-008 and Toripalimab)

Qualified subjects must initiate treatment promptly after signing the Informed Consent Form (ICF). A window of ±3 days is allowed for all subsequent visits, except in cases where special instructions apply (e.g., PK blood sampling).

Subjects will receive oral ATG-008 (once daily) in combination with intravenous infusion of Toripalimab (fixed dose of 240 mg, every 3 weeks), and PK samples will be collected (only during the dose escalation phase). Each cycle consists of 21 days, until radiological disease progression occurs, intolerable toxicity is observed, or the subject withdraw informed consent and refuses to continue treatment (in which case, the subject will continue to receive

further anticancer therapy and be followed for survival, unless follow-up is explicitly withdrawn). Subjects must fast for at least 6 hours prior to visits for PK assessments and fasting chemistry tests.

Visits during the treatment cycles will occur on days 1, 8, and 15 of cycles 1 and 2, followed by visits on the first day of each subsequent cycle.

Day 1 of Cycle 1 (C1D1):

1. Verification of inclusion and exclusion criteria.
2. Measurement of body weight.
3. Physical examination for symptoms.
4. Vital signs assessment.
5. ECOG performance status evaluation.
6. Child-Pugh score assessment (for hepatocellular carcinoma [HCC] subjects only) *.
7. 12-lead ECG.
8. Cardiac enzyme profile *.
9. Complete blood count *.
10. Fasting blood chemistry (including fasting blood glucose) *.
11. Urinalysis *.
12. Coagulation profile *.
13. Thyroid function tests *.
14. HBV-DNA testing should be performed in each cycle if HBsAg is positive, anti-HBc antibodies are positive, or HBV-DNA is detectable *. If HBV-DNA levels increase during treatment (in subjects with detectable HBV at baseline) or if HBV-DNA becomes detectable (in subjects with undetectable HBV-DNA at baseline), HBsAg, HBeAg, and anti-HBc antibody testing should be conducted.
15. Women of childbearing potential should undergo urine or blood pregnancy testing on day 1 of each cycle. If the urine pregnancy test is positive, it should be confirmed by serum pregnancy testing.
16. Quantitative AFP testing (for HCC subjects only) *.
17. Quantitative CA125 testing (for ovarian cancer subjects only; for subjects achieving CA125 response, a CA125 re-evaluation should be conducted 4 weeks later to confirm response; for subjects with CA125 normalization after treatment, at least two additional CA125 tests should be performed one week apart to determine PD [definitions of CA125 response and disease progression according to GCIG criteria are provided in Appendix 11]) *.
18. PK blood sampling (refer to Section 7.10).
19. Serum ADA testing: Samples should be collected within 60 minutes before each Toripalimab administration during the first 6 cycles of the treatment period.
20. Subjects will receive intravenous infusion of Toripalimab at a fixed dose of 240 mg at the research center, followed by oral administration of ATG-008 1-2 hours after the infusion is completed. The remaining doses of medication for weeks 1-3 will be provided to the subjects for self-administration.
21. Record concomitant medications and all adverse events occurring after medication administration.

*If assessed within the first 7 days before initial dosing, C1D1 does not need to be repeated.

Days 8 and 15 of Cycle 1 (C1D8 and C1D15):

1. Vital signs monitoring.
2. 12-lead ECG: when clinically indicated.
3. Cardiac enzyme profile: when clinically indicated.
4. Complete blood count.
5. Fasting blood chemistry (including fasting blood glucose).
6. PK blood sampling (C1D15, refer to Section 7.10).

7.Administration of ATG-008.

8.Record concomitant medications and all adverse events occurring after medication administration.

Day 1 of Cycle 2:

1.HbA1c measurement; subsequently, once every 2 cycles (day 1).

2.12-lead ECG: when clinically indicated.

3.Cardiac enzyme profile: when clinically indicated.

4.Other procedures identical to those on day 1 of Cycle 1 (excluding verification of inclusion and exclusion criteria and PK blood sampling).

Days 8 and 15 of Cycle 2:

1.Vital signs monitoring.

2.12-lead ECG: when clinically indicated.

3.Cardiac enzyme profile: when clinically indicated.

4.ATG-008 administration at the research center, with remaining doses for self-administration by the subject.

5.Record concomitant medications and all adverse events occurring after medication administration.

Day 1 of Cycle 3 and subsequent Cycle 1 days:

1.12-lead ECG: when clinically indicated.

2.Cardiac enzyme profile: when clinically indicated.

3.Treatment response assessment/tumor evaluation: every 6 weeks ±7 days within the first 12 months of treatment; every 12 weeks ±7 days after 12 months of treatment; or as clinically necessary.

4.Other procedures identical to those on day 1 of Cycle 1 (excluding verification of inclusion and exclusion criteria and PK blood sampling).

6.3. End-of-Treatment Visit (0-7 days after the end of treatment)

The end-of-treatment visit will occur 0-7 days after the completion of the last dose of ATG-008 and Toripalimab for all subjects, including those who prematurely terminate treatment, as detailed in the study flowchart (Table 1):

1.Measurement of body weight.

2.Vital signs monitoring.

3.Comprehensive physical examination (as per Section 6.1).

4.ECOG performance status assessment.

5.Child-Pugh score assessment (for HCC subjects only).

6.12-lead ECG.

7.Cardiac enzyme profile: when clinically indicated.

8.HBV and HCV testing (as per Section 6.2).

9.Complete blood count.

10.Fasting blood chemistry (including fasting blood glucose).

11.Urinalysis.

12.Coagulation function testing.

13.Thyroid function testing.

14.HbA1c measurement.

15.Quantitative AFP testing (for HCC subjects only).

16.Quantitative CA125 testing (for ovarian cancer subjects only).

17.Serum pregnancy testing (for women of childbearing potential).

18.Serum ADA testing.

19.Record concomitant medications and adverse events.

20.Record any additional anticancer treatment received by subjects after the completion of

ATG-008 and Toripalimab combination therapy.

For subjects who discontinue study medication for reasons other than "radiological disease progression," tumor evaluations will continue according to the study flowchart, without the need for a specific end-of-treatment visit.

6.4. Safety Follow-up (30 days ± 7 days after the last dose)

All subjects will be monitored for new adverse events or follow-up on existing adverse events for 30 days after receiving the last dose of study medication. For any clinically significant assessments or abnormal laboratory findings, additional unscheduled assessments should be conducted until the issues are resolved, as determined by the investigator.

For all subjects who discontinue treatment due to reasons other than disease progression, death, or withdrawal of consent for follow-up, treatment response assessments and collection of new anticancer treatment information will be conducted according to the provisions of Section 7.11.

6.5. Information to be collected during safety follow-up includes:

- Measurement of body weight.
- Vital signs monitoring.
- Physical examination for symptoms.
- ECOG performance status assessment.
- Child-Pugh score assessment (for HCC subjects only).
- 12-lead ECG.
- Cardiac enzyme profile: when clinically indicated.
- HBV and HCV testing (as per Section 6.2).
- Complete blood count.
- Fasting blood chemistry (including fasting blood glucose).
- Urinalysis or serum pregnancy testing (for women of childbearing potential), with confirmation by serum pregnancy testing if the urine pregnancy test is positive.
- Serum ADA testing.
- Record concomitant medications and adverse events.
- Record any additional anticancer treatment received by subjects after the completion of ATG-008 and Toripalimab combination therapy.

6.6. Survival follow-up

All the subjects who received treatment after admission to the group were followed up for survival. From the last drug administration date of the study to the end of the study, the subjects (or the subjects' families) were contacted by telephone or in person every 12 weeks (±14 days) to determine the survival status of the subjects, to track the status of serious adverse events related to the study drug, and any new antitumor therapy used by the subjects after the termination of the combined treatment of ATG-008+ toripalimab. If the subject has died, the date of death and the cause of death (if possible) are collected.

For the missing subjects, the researchers should record in the original document all the efforts made by the subjects, such as the date of telephone contact and other contact information.

6.7. Restrictions and considerations

Medication: During this study period, participants are not permitted to use any other investigational drugs to treat any conditions. Participants must report all prescription and over-the-counter medications they receive to their investigators during the study period.

Diet: There are no dietary restrictions in this study. Participants should maintain an adequate intake of calories and fluids.

7. Evaluation method

7.1. Demographic data

Demographic data on the subjects, including date of birth, sex and nationality, will be collected during screening visits.

7.2. Medical history and treatment history

During screening visits, a complete history of cancer, including specific information on diagnosis, staging, and histology, as well as other meaningful medical conditions, was collected for each subject.

During screening visits, previous cancer treatments will be recorded: including surgery, radiotherapy, systemic and local areas, or any other treatment (such as hormones) for the subject's cancer (also record the causes of previous treatment discontinuation, such as disease progress, intolerance, or others). And the past and accompanying drug use evaluation of the subjects.

7.3. Combination of drugs

In addition to studying drugs (ATG-008 and Toripalimab), all subjects in this study also received optimal supportive treatment, which was determined by the researchers in accordance with local / hospital guidelines. Optimal support therapy is defined as nonspecific antitumor therapy, which maximizes the quality of life, including antibiotics, painkillers, antiemetic drugs, thoracic puncture, pleural fixation, blood transfusion, nutritional support, except surgery, transcatheter arterial chemo embolism, radiofrequency ablation, immunotherapy, anticancer hormone therapy, systemic chemotherapy and radiotherapy However, palliative radiotherapy for non-target lesions (such as sustained-release pain as the main purpose) and bisphosphate therapy for bone metastases were allowed during the study period. There is no need to terminate treatment alone because of palliative radiotherapy.

7.4. Physical examination and ECOG score

At the end of the screening and treatment visit, a complete physical examination will be conducted (including general condition, skin, head, neck, ear, nose, mouth, throat, respiratory/lung, cardiovascular, gastrointestinal/abdominal, reproductive urethra, neurology, Musculoskeletal/limbs and lymphoid system, as well as any other items that may be relevant). The other symptom-oriented physical examinations carried out in this study were carried out according to the corresponding symptoms of the subjects.

Medical examination information must be recorded in the original file of the research center. Clinical results that meet the definition of adverse events after initial administration of ATG-008 and Toripalimab must be recorded in eCRF.

ECOG physical fitness assessment (Appendix 4) will take place on the screening visit, the 1st day of each cycle, and at the end of the treatment.

7.5. Vital signs

The vital signs of the subjects, including blood pressure, pulse, respiratory frequency and body temperature, were measured during each visit. The subjects were measured after lying on their backs or resting in a sitting position for 5 minutes. If possible, the vital signs of the subjects should be evaluated before administration on the day of the visit.

7.6. Height and weight

Height (cm) will be measured only during screening, such as the research process table (Table 1). The subject weight (kg) will be recorded at the end of the screening, the 1st day of each cycle, the end of treatment, and the safe follow-up.

7.7. Evaluation of Child-Pugh score (HCC subjects only)

During the dose expansion phase of the study, according to the 2018 version of the guidelines for the diagnosis and treatment of primary liver cancer, subjects with HCC diagnosed pathologically or clinically were enrolled in the study. The stage of HCC was confirmed according to the clinical stage of liver cancer in Barcelona, and the Child-Pugh grade score was determined (Appendix 5).

7.8. Evaluation of KPS Score (GBM Only)

In the dose expansion phase of the study, GBM participants who are histologically diagnosed will undergo Karnofsky Performance Status (KPS) assessment to confirm a KPS score ≥70 (Appendix 8).

7.9. Safety Assessment

Safety monitoring will involve evaluating adverse events, concomitant medications, laboratory parameters, physical examinations, vital signs, weight, ECOG status, and ECG results. Adverse event information will be collected at each visit and graded according to NCI-CTCAE 5.0 criteria.

Standard 12-lead ECG examinations will be conducted at screening, end of treatment visits, and at other clinical indications. Participants must rest for at least 5 minutes before ECG recording. ECG interpretations will be categorized as normal, abnormal without clinical significance, or abnormal with clinical significance. ECG details including date, time, heart rate, PR interval, QT interval, QRS interval, and corrected QT interval (see Appendix 7) will be recorded in the electronic Case Report Form (eCRF).

The study will include various clinical laboratory tests as follows:

Complete Blood Count:

Hematologic assessments will be conducted at screening visits, during the first week (days 1, 8, and 15), in subsequent cycles, at treatment end visits, and during safety follow-ups. Parameters examined will include hemoglobin, hematocrit, mean corpuscular volume, mean corpuscular hemoglobin content, mean corpuscular hemoglobin concentration, white blood cell count, differential white cell count, red blood cell count, lymphocytes, monocytes, neutrophils, eosinophils, basophils, and platelets. Manual or automated white cell differentials will be conducted as per institutional standards. Reticulocyte count will be performed only if clinically indicated.

Biochemical Tests:

Biochemical evaluations will be performed at screening visits, during the first week (days 1, 8, and 15), in subsequent cycles, at treatment end visits, and during safety follow-ups. Test parameters will include sodium, potassium, chloride, bicarbonate, blood urea nitrogen/urea, creatinine, glucose, calcium, phosphorus, magnesium, ALT, AST, alkaline phosphatase, total bilirubin, lactate dehydrogenase, total protein, albumin, amylase, lipase, creatine kinase, and uric acid.

Urinalysis:

Urine tests will be carried out at screening visits, on the first day of each cycle, and at treatment end visits. Parameters examined will include bilirubin, glucose, occult blood, ketones, pH, protein, specific gravity, and urobilinogen. Microscopic examination of urine samples will be conducted only when clinically indicated.

HBV, HCV, and HIV Testing:

Testing for HBV, HCV, and HIV will be performed at screening. HBV-DNA testing should be included in each cycle if HBsAg is positive, anti-HBc antibody is positive, or HBV-DNA is detectable. Additional testing for HBsAg, HBeAg, and anti-HBc antibodies may be warranted if HBV-DNA levels increase during treatment or the virus becomes detectable in participants initially negative for HBV-DNA. Thyroid Function Testing: T3, T4, and TSH tests will be conducted at screening, on the first day of each cycle, and at treatment end visits.

Coagulation Function Testing:

Assessment of coagulation parameters, including prothrombin time, international normalized ratio, and activated partial thromboplastin time, will be performed at screening visits, on the first day of each cycle, and at treatment end visits. Repeat testing may be done as needed in case of coagulation-related issues.

Pregnancy Testing for Women of Childbearing Potential:

Serum human chorionic gonadotropin (hCG) pregnancy testing will be done at screening and treatment end visits. Urine or blood pregnancy testing will be conducted on the first day of each cycle and during safety follow-ups for women of childbearing potential. A positive urine pregnancy test will be confirmed with a serum pregnancy test.

ANA Testing:

ANA testing will only be conducted at screening.

HbA1c Testing:

HbA1c testing will be carried out at screening, every two cycles post-treatment, and at treatment end visits.

Cardiac Enzyme Profile Testing:

Cardiac enzyme tests, including serum creatine kinase and creatine kinase MB, will be performed at screening, on the first day of the first cycle, and as clinically indicated.

AFP or CA125 Testing:

Quantitative AFP testing will be conducted for HCC participants at screening visits, on the first day of each cycle, and at treatment end visits. Quantitative CA125 testing will be done for ovarian cancer participants at the same time points.

MSI, TMB, and PD-L1 Testing:

Exploratory testing for MSI, TMB, PD-L1 levels in tumor tissue, as well as monitoring for specific pathway mutations (including but not limited to PI3K, AKT, KEAP1, NFE2L2, STK11), will be performed during screening visits. Additionally, blood samples for sPD-L1 testing may be collected as needed.

7.10. Pharmacokinetic Assessment (Dose Escalation Phase Only)

During dose escalation, blood samples (3 mL/sample) for PK analysis of ATG-008 will be collected from each participant at various time points to evaluate the PK characteristics of ATG-008 and its metabolites post-combination therapy.

PK Sampling Timepoints: The sampling intervals for ATG-008 will include time points before the first dose, 15 minutes post-dose, 0.5 hours ±5 minutes post-dose, 1 hour ±10 minutes post-dose, and subsequent intervals up to 48 hours. Additional samples at various time points will be collected according to the study protocol.

Information including drug dosage, actual administration time, actual sampling time, and planned sampling time must be accurately recorded during PK sample collection. Detailed procedures for sample collection, processing, storage, and transportation can be found in the laboratory manual.

7.11. Efficacy Assessment

7.11.1. Treatment Response Evaluation

Participants will be assessed for tumor response using the Response Evaluation Criteria in Solid Tumors (RECIST 1.1) or Response Assessment in Neuro-Oncology (RANO [GBM only]) criteria.

At screening, all participants must undergo CT or MRI imaging of the chest, abdomen, pelvis, and cranial MRI based on RECIST 1.1 or RANO protocols. Participants with bone lesions must also undergo confirmatory bone scans. Tumor baseline assessment should occur no more than 28 days before treatment initiation. Imaging evaluations will be continued throughout the study following a consistent imaging modality as used during the screening phase.

During the extension phase of the study, blood vessel invasion and extrahepatic spread in HCC will be defined for stratification purposes. Tumor assessment will be conducted at 6 weeks after initial treatment, every 6 weeks ±7 days within the first 12 months post-treatment, and every 12 weeks ±7 days after 12 months of treatment. Evaluations will continue until disease progression, initiation of new anti-cancer therapy, death, or withdrawal of informed consent, whichever occurs first.

For solid tumors (excluding GBM), RECIST 1.1 or iRECIST will be applied, while GBM will be assessed using RANO or iRANO criteria. Participants showing evidence of tumor response (complete response [CR] or partial response [PR]) on imaging will require confirmation at the following scheduled scans with a minimum interval of 28 days. When stable disease (SD) is considered the best response, the efficacy evaluation should be at least 5 weeks from the initial treatment date.

In cases where participants discontinue study drug due to toxicity, participant or investigator decision, tumor assessment should still continue as per the protocol. Additionally, in instances where participants continue treatment post-disease progression on imaging until clinical deterioration (see Appendix 1), tumor assessments should continue until the cessation of study treatment.

New anti-cancer therapies may consist of any systemic therapy, local treatment, surgery, radiotherapy, or other intervention used to treat the participant's cancer.

7.11.2. Serum Anti-Drug Antibody (ADA) Assessment

During the treatment phase, blood samples for serum anti-drug antibody (ADA) detection will be collected 60 minutes before each Toripalimab dose during the first 6 cycles. Subsequent collection will occur every 2 cycles on the first day of the cycle until the end of treatment visit and safety follow-up visits to investigate potential relationships between Toripalimab-ADA and efficacy endpoints.

7.12. Exploratory Evaluation

Descriptive analyses will be performed to summarize the response rates of AFP in HCC participants.

For ovarian cancer participants, CA125 changes will be evaluated in conjunction with GCIG criteria and RECIST 1.1. Participants achieving CA125 relief will undergo CA125 retesting after 4 weeks to confirm resolution status. Those whose CA125 levels return to normal post-treatment will require at least two additional CA125 tests at weekly intervals to definitively assess for progressive disease (PD) [see Appendix 11 for GCIG-defined CA125 relief and disease progression]. Descriptive analyses will be conducted to summarize participants' levels of MSI, TMB, PD-L1 (tumor tissue or blood), relevant pathway mutations (including but not limited to PI3K, AKT, mTOR, KEAP1, NFE2L2, STK11), and other relevant exploratory endpoints related to the study drug.

8.Termination Criteria

8.1. Premature Termination of the Study

The sponsor may decide to terminate the study for any reason, including medical or ethical reasons affecting the continuation of the study, or difficulties in subject recruitment. Medical reasons may include unacceptable risks to subjects, lack of efficacy, serious adverse events such as Grade 4 anorexia, and post-treatment fatigue without efficacy.

8.2. Premature Treatment Termination of Individual Subjects

The investigator may consider allowing subjects to withdraw from the study for the following reasons:

1.Disease progression, confirmed by investigator assessment (RECIST 1.1 or RANO criteria).

For subjects with imaging evidence of disease progression, if there is clear evidence of clinical benefit/stability, the investigator may choose to continue the subject in the study and receive ATG-008 Toripalimab monoclonal antibody combination therapy after discussion with the sponsor, but continuous monitoring of subject's disease status is required.

2.Clinically determined disease progression by the investigator (if possible, confirmed by radiographic imaging, and the investigator must maintain comprehensive records).

3.Unacceptable adverse events or intolerance to ATG-008 + Toripalimab monoclonal antibody combination therapy.

4.Discontinuation for more than 28 days, except in special cases approved by the sponsor.

5.Any medically reasonable reasons determined by the investigator, or significant protocol violations.

In case of any of the following reasons, the investigator must discontinue ATG-008 + Toripalimab monoclonal antibody combination therapy for the subject:

1.Subject decision to terminate treatment or withdrawal of consent.

2.Pregnancy.

Subjects may decide to terminate ATG-008 + Toripalimab monoclonal antibody

combination therapy for any reason. If a subject chooses to terminate study treatment, they should be encouraged to continue participating in the study for follow-up information on disease progression and survival status. However, subjects may choose to withdraw consent at any time and refuse further participation in the study.
All subjects will receive follow-up until the end of the study or withdrawal of consent, investigator-initiated discontinuation, subject death, or loss to follow-up (whichever occurs first).

8.3. Study Termination

Study termination is defined as 12 months after the last subject is enrolled, withdrawal of consent, death, or loss to follow-up (whichever occurs first). In addition, the sponsor may decide to terminate the study at any time for reasons including but not limited to safety concerns.

After the completion of the study, the sponsor will continue to provide study medication to subjects with clinical benefit (as determined by the investigator and agreed upon by the sponsor) as needed, and continue to collect safety data through voluntary reporting. The study medication may be provided through additional expanded studies or other forms at the discretion of the sponsor.

9.Treatment Research

This study will employ combination therapy, investigating drugs including ATG-008 and toripalimab monoclonal antibody.

The drug provider will supply ATG-008 tablets in specifications of 5mg, 15mg, 20mg, and 30mg. ATG-008 tablets are traditional solid oral dosage forms manufactured with excipients and should be stored at room temperature (15-25 degrees Celsius).

Shanghai Junshi Biosciences Co., Ltd. will provide toripalimab monoclonal antibody (toripalimab monoclonal antibody injection), a colorless or pale-yellow clear liquid with slight opalescence. The main active ingredient of toripalimab monoclonal antibody (per vial) is toripalimab monoclonal antibody (240.00 mg), with a volume of 6 mL/vial, antibody concentration of 40.0 mg/mL, and excipients including citric acid monohydrate (3.06 mg), sodium citrate dihydrate (31.80 mg), sodium chloride (17.52 mg), mannitol (150.00 mg), and Polysorbate 80 (1.20 mg). It should be stored and transported between 2-8 degrees Celsius, protected from light, and should not be frozen or vigorously shaken.

For detailed information on the investigational drugs, please refer to the ATG-008 and toripalimab monoclonal antibody investigator's manual.

In addition to the investigational drugs, all subjects may receive best supportive care, as determined by the investigator according to local/hospital guidelines (see Section 7.3). Note: ATG-008 and toripalimab monoclonal antibody may be temporarily suspended and used individually based on clinical practice.

9.1. Administration Sequence

ATG-008 should be taken at the same time every morning. When administered on the same day as toripalimab monoclonal antibody, subjects should visit the research center after fasting for 6 hours, complete all fasting examinations before eating (light diet), and take the investigational drugs after eating. Treprostinil monoclonal antibody should be administered intravenously first, followed by oral administration of ATG-008 1-2 hours after toripalimab monoclonal antibody injection.

9.2. Drug Labeling

ATG-008 tablets and toripalimab monoclonal antibody will be labeled according to the current International Conference on Harmonization (ICH) requirements for human drug registration, Good Clinical Practice (GCP), and specific requirements of Chinese regulatory authorities. The drug labels will be in Chinese and comply with Chinese legal requirements, including storage conditions.

9.3. Drug Compliance

Investigators or designated personnel will assess the compliance of ATG-008 and

toripalimab monoclonal antibody treatment at each subject visit and record the subject's treatment compliance in the source documents after discussing drug counts with the subject. The primary investigator or designated personnel will reconcile the returned drugs from the subjects and calculate the number of ATG-008 tablets dispensed. Any deviations and missed doses will be recorded in the electronic Case Report Form (eCRF), and the reasons for discrepancies and missed doses will also be recorded in the drug count log. Investigators/designated personnel will provide timely guidance to subjects to ensure compliance with the dosing regimen. All unused toripalimab monoclonal antibody will be stored at the designated storage location in the clinical pharmacology department of the research center, following specified storage conditions (protected from light, refrigerated at 2-8°C). The drug provider will regularly arrange personnel to collect used toripalimab monoclonal antibody residues and empty bottles from the research center. If drugs are lost or damaged, detailed records should be kept. At the end of the study, any unused toripalimab monoclonal antibody and used residues and empty bottles must be uniformly collected and returned to the drug provider or a designated third party.

9.4. Overdose

From a dosing perspective, overdose is defined as a specific subject using more than the study-specified investigational drug dose, regardless of whether there are any related adverse events or sequelae. Based on dosing frequency, overdose is defined as the frequency of use of any compound exceeding the frequency required by the study protocol. Complete data on drug use, including any instances of overdose, whether accidental or intentional, should be reported in the eCRF. See Section 10.5 for adverse event reporting related to overdose.

9.5. ATG-008

9.5.1. Dosage and Administration of ATG-008

ATG-008 will be administered orally, once daily (QD), with each dosing cycle lasting 21 days.

9.5.2. Management of ATG-008-Related Adverse Reactions

Hyperglycemia

Hyperglycemia is a known class effect of mTOR inhibitors related to the mechanism of action of the drug. Subjects' blood glucose should be closely monitored, and anti-hyperglycemic medications should be used for management according to protocol guidelines.

Pneumonia

Drug-related non-infectious pneumonia is a category effect of drugs targeting the mTOR pathway, reported under ATG-008 treatment. In subjects with non-specific respiratory signs and symptoms and after ruling out infection, tumor-related causes, and other reasons through appropriate examination, pneumonia should be considered for diagnosis.

Gastrointestinal Symptoms

ATG-008 treatment has been associated with drug-related gastrointestinal toxicity (nausea, vomiting, diarrhea, stomatitis, and decreased appetite), which may be associated with the risk of dehydration, electrolyte imbalance, and sharp decline in estimated glomerular filtration rate. These toxicities in subjects should be closely monitored, and ATG-008 administration should be temporarily suspended or the dose adjusted based on medical indications, along with appropriate hydration and supportive care. Subjects with dehydration-related hypovolemia may experience hypotension. Subjects should be informed that hypotension and orthostatic dizziness may be early signs of dehydration, and if they experience these symptoms, they should contact their research center.

Pancreatitis

Pancreatitis and asymptomatic elevations of amylase and lipase have been reported under ATG-008 treatment. Monitoring should include routine amylase and lipase laboratory tests. Temporary discontinuation of ATG-008 administration or dose adjustment may be necessary

to manage these events according to protocol guidelines.

Infection

The use of ATG-008 may lead to immunosuppression, making subjects more susceptible to infection. Subjects should be carefully monitored for infection, including opportunistic infections. Routine infectious disease prophylaxis is not recommended; however, antibiotics, antivirals, anti-Pneumocystis jirovecii agents, antifungals, or other preventive measures may be administered during the study process based on the investigator's decision.

Photosensitivity

ATG-008 absorbs ultraviolet light between 290 and 700 nm and has been shown in non-clinical animal studies to be distributed to the skin and eyes. As a preventive measure, subjects are advised to avoid prolonged exposure to ultraviolet light during ATG-008 administration, wear protective clothing and sunglasses, and use topical preparations with UV protection.

9.5.3. Dose Adjustment of ATG-008

For managing adverse events (such as gastrointestinal reactions), it can be decided by the investigator and subject to switch to nighttime dosing starting from Day

Table2 Recommended guidelines for reductions for suspected ATG-008-related toxic reactions

Adverse events	Level	Measures
Thrombopenia	3	If bleeding occurs, the dose of ATG-008 is reduced by one level.
	4	If it lasts more than 7 days, suspend the use of ATG-008 until the event level is ≤ 2 After that, the dose of ATG-008 decreased by one level.
Neutrophil reduction	3	If fever occurred, the dose of ATG-008 was reduced by one level.
	4	If it lasts more than 7 days or is accompanied by fever, suspend the use of ATG-008 until the event level is ≤ 2. The dose of ATG-008 was then reduced by one level.
Increase in the level of bilirubin	3	If > 5 × ULN, pause ATG-008 until event level ≤ 2. The dose of ATG-008 was then reduced by one level.
	4	Pause ATG-008 until event level ≤ 2. The dose of ATG-008 would be reduced by one level.
Elevation of transaminase	3	If > 10 × ULN, pause ATG-008 until event level ≤ 2. The dose of ATG-008 was then reduced by one level.
	4	Pause ATG-008 until event level ≤ 2. The dose of ATG-008 was then reduced by one level.
Stomatitis / mucositis / vomiting / diarrhea	3	If optimal medical management is not possible within 7 days, reduce the dose of ATG-008 by one level.
	4	Pause ATG-008 until event level ≤ 2. Then reduce the dose of ATG-008 by one level.
Hyperglycemia	3 / 4	If optimal medical management is not possible within 7 days, reduce the dose of ATG-008 by one level. See also Appendix 3.
Skin rash	3	If there is no response to the best medical management within 7 days, ATG-008 is suspended until the event level is ≤ 2. The dose of ATG-008 was then reduced by one level.
	4	Deactivate ATG-008
Local acute pneumonia	1	Continue to use ATG-008 and monitor it closely.
	2	Pause ATG-008 until event level ≤ 1. After that, maintain the dose or reduce the dose of ATG-008 by a level.

	3	Deactivate ATG-008
Fatigue	3	If there is no response to the best medical treatment within 7 days, suspend ATG-008 until event level ≤ 2.
Others	3	Reduce ATG-008 reduction by one level.
	4	Deactivate ATG-008.

9.6. Toripalimab Monoclonal Antibody

Toripalimab monoclonal antibody will be administered at a fixed dose of 240 mg via intravenous infusion every 3 weeks (Q3W). The maximum duration of treatment with Toripalimab monoclonal antibody will not exceed 2 years, until the occurrence of confirmed disease progression on imaging, intolerable toxicity, withdrawal by the subject or investigator, or subject death, whichever comes first.

9.6.1. Preparation, Use, and Storage of Toripalimab Monoclonal Antibody

The investigational drug, Toripalimab monoclonal antibody, must be carefully inspected prior to use to ensure each vial is intact, with no signs of damage, and the solution inside is free from clotting, turbidity, or precipitation before use. It is recommended to withdraw the investigational drug using a syringe and inject it into 100 mL of normal saline. Gently invert the mixture 3-5 times, avoiding vigorous shaking, and then use an in-line filter (0.2 or 0.22 μm) for intravenous infusion. The final concentration of the diluted drug should be 0.2-20.0 mg/mL. Unused portion of the drug should not be reused, and it should not be diluted with 5% glucose solution or mixed with other drugs. The diluted solution should be stored and transported between 2-8°C, protected from light, and should not be frozen or vigorously shaken. Diluted solution prepared under aseptic conditions can be stored for up to 12 hours at 2-8°C or room temperature, but it is recommended to use within 4 hours. Toripalimab monoclonal antibody injection solution, once prepared, should be brought to room temperature if refrigerated and should not be frozen.

9.6.2. Management of Hypersensitivity Reactions

Antibody administration may cause hypersensitivity reactions. Therefore, immediate provision of appropriate medications and medical equipment to treat acute hypersensitivity reactions is crucial. Study personnel must be trained to identify and manage hypersensitivity reactions. The study site must be equipped with emergency response teams and equipment, and the capability to admit subjects to intensive care units if necessary. In the event of severe hypersensitivity reactions, subjects must receive immediate emergency treatment according to local clinical protocols. Subjects must receive adrenaline and dexamethasone immediately and undergo electrocardiographic monitoring. Serum IgE samples may also be considered for extraction. Subjects experiencing such symptoms must immediately inform the investigator. Subjects experiencing hypersensitivity reactions must discontinue treatment immediately and withdraw from the study.

9.6.3. Management of Infusion Reactions

The use of any recombinant protein may lead to infusion reactions. Clinical symptoms of infusion reactions include fever, chills, rigors, headache, rash, itching, joint pain, hypotension or hypertension, bronchospasm, among others. To promptly treat infusion-related reactions, it is advisable to prepare necessary medications before starting infusion and place them readily accessible near the bedside as per routine for handling infusion-related reactions.

If symptoms of infusion reactions occur (e.g., feeling cold, rigors, fever, neck pain, nausea), infusion should be paused, and the subject should be assessed for other signs or symptoms indicative of more severe reactions (such as hypotension, hypoxia, wheezing, urticaria). Vital signs should be monitored every 15 minutes until symptoms subside.

Medication for treating infusion reactions will be administered by the investigator according to local best medical practices. In cases of severe reactions, cardiac monitoring and administration of rescue medications (including but not limited to adrenaline, corticosteroids, antihistamines, bronchodilators, and oxygen) are required. Infusion of Toripalimab monoclonal antibody must be immediately discontinued in the event of Grade 2 or higher infusion reactions or suspected events. During the first intravenous infusion of Toripalimab monoclonal antibody, an in-line filter (0.2 or 0.22 μm) should be used, and infusion should be completed within 60 minutes. If infusion reactions occur, infusion speed can be slowed or interrupted, and necessary interventions should be provided until the subject's symptoms improve, then continue infusion at half the previous rate. Vital signs should be monitored for

30 minutes after infusion completion. Subjects will be informed of possible delayed infusion-related symptoms and instructed to contact their study physician if such symptoms occur. Treatment adjustment guidelines for infusion reactions are detailed in Table 3. If subjects experience Grade 2 or higher infusion reactions again, they must immediately discontinue treatment and withdraw from the study.

Table 3 Guidelines for the adjustment of treatment for infusion response of Toripalimab

CTCAE level	Adjustment measures
Level 1-slight	Transient mild reaction, it is not recommended to interrupt infusion and clinical intervention. Slow the drip rate by 50% and closely observe any deterioration symptoms. If necessary, clinical intervention is carried out.
Level 2-medium	Suspend Toripalimab monoclonal antibody treatment and immediately administer systemic therapy (such as antihistamines, NSAIDs, analgesics, intravenous fluids); when infusion reaction alleviates to Grade 0-1, resume administration at reduced infusion rate by 50%. Monitor closely for any worsening symptoms during this period. Implement appropriate therapeutic interventions according to local medical protocols.
Level 3-serious	Stop the infusion immediately and remove the infusion tube. Based on the actual situation of the subjects, the researchers discussed with the drug providers whether to resupply[a].
	If readministered, during follow-up treatment, the infusion time is at least 2 hours and needs to be given<br>Preventive use of drugs (such as antihistamine drugs and NSAID drugs) requires close observation of the clinical symptoms of the related infusion reactions at the same time.
	Appropriate treatment interventions should be taken according to local medical practices
Level 4-Life-threatening and requiring urgent clinical intervention	Subjects with a level 4 infusion response must immediately and permanently stop the drug and withdraw from the study. Appropriate treatment interventions should be taken according to local medical practices.

NSAID= Non-Steroidal Anti-Inflammatory Drug

a: Participants who still benefit from Toripalimab monoclonal antibody treatment and fully recover from infusion reactions may consider continuing Toripalimab monoclonal antibody treatment, only after consultation between the investigator and the drug provider, based on overall risk-benefit assessment. Re-initiation of Toripalimab monoclonal antibody treatment is permissible upon approval and documentation by the drug provider.

9.6.4. Management of Immunotherapy-Related Adverse Events and Other Special Adverse Events

The mechanism of action of Toripalimab monoclonal antibody can lead to T cell activation and proliferation, which may result in irAEs during the implementation of clinical studies. Therefore, monitoring of the immunological toxicity of Toripalimab monoclonal antibody, including signs and symptoms of irAEs in participants, should be conducted during clinical trial processes. In the absence of alternative causes (such as infection or disease progression), signs or symptoms of inflammatory events such as myocarditis, enterocolitis, pneumonia, dermatitis, hepatitis, and endocrine disorders should be considered as potential immunological events. General management principles for immunotherapy-related adverse events are outlined in Table 4, with detailed information on the management of other immunotherapy-related adverse events available in the Toripalimab monoclonal antibody investigator's manual.

Immunotherapy-Related Interstitial Lung Disease

Possible symptoms include dyspnea, cough, fatigue, hypoxia, pneumonia, and pulmonary infiltrates, primarily in participants with additional risk factors such as a history of lung cancer, interstitial pneumonia, or prior radiotherapy. Lung signs and symptoms should be assessed throughout the study, with chest CT scans performed on participants at each tumor assessment. Comprehensive evaluation of all pulmonary adverse events should be conducted, including common causes such as pneumonia/infection, lymphangitic carcinomatosis,

pulmonary embolism, heart failure, or chronic obstructive pulmonary disease or pulmonary arterial hypertension: oxygen saturation measurement (i.e., arterial blood gas); high-resolution chest CT scan; bronchoscopy and bronchoalveolar lavage and biopsy; pulmonary function tests; implementation of pulmonary function tests according to the pulmonary embolism plan.

Immunotherapy-Related Hypothyroidism and Hyperthyroidism

Participants presenting with symptoms of unknown origin (such as fatigue, myalgia, erectile dysfunction, changes in mental status, or constipation) should be evaluated for thyroid function and hyponatremia or hyperkalemia. If an endocrine disorder is suspected, an endocrinologist consultation should be sought. Measurement of TSH and free T3 or T4 levels should be performed to assess for thyroid abnormalities.

Immunotherapy-Related Adrenal Insufficiency

Clinical manifestations of adrenal insufficiency include fatigue, lethargy, anorexia, nausea, weight loss, dizziness, and orthostatic hypotension. Laboratory findings may reveal morning cortisol deficiency, elevated morning adrenocorticotropic hormone (ACTH), and orthostatic hypotension resulting from aldosterone deficiency, hypovolemia, hyponatremia, and hyperkalemia.

Immunotherapy-Related Pancreatitis

Acute pancreatitis presents with abdominal pain and elevated levels of amylase and lipase. Therefore, when participants experience symptoms of acute abdominal pain, pancreatitis or acute pancreatitis should be considered in the differential diagnosis. Appropriate investigations include assessment for obstruction, as well as serum amylase and lipase testing.

Immunotherapy-Related Hyperglycemia and Diabetes

Occurrences of hyperglycemia/diabetes have been observed during Toripalimab monoclonal antibody use. Participants with a history of diabetes must have their blood sugar controlled before medication. Blood sugar should be closely monitored in all participants throughout the study, with monitoring achieved through glycated hemoglobin and blood glucose monitoring.

Immunotherapy-Related Hepatic Dysfunction

Immunotherapy-related hepatic dysfunction may be associated with Toripalimab monoclonal antibody administration. Participants must have good liver function, and liver function should be closely monitored throughout the entire study treatment period. If participants present with right upper quadrant pain and/or unexplained nausea or vomiting during the study, liver function testing should be performed immediately, and the results reviewed before the next study drug administration. If liver function tests are elevated, consideration should be given to concurrent medication, viral hepatitis, and conditions such as treatment toxicity or tumor recurrence. Liver, gallbladder, and biliary system imaging should be performed to exclude tumor-related or other causes of elevated liver function tests. If autoimmune causes are suspected, anti-nuclear antibodies, anti-neutrophil cytoplasmic antibodies, anti-liver kidney microsomal antibodies, and anti-smooth muscle antibodies tests should be conducted.

Immunotherapy-Related Skin Toxicity

Rashes occurring during treatment may be related to Toripalimab monoclonal antibody therapy. Evaluation of persistent and/or severe rashes or pruritus by a dermatologist should be considered unless contraindicated, biopsy may be considered.

Immunotherapy-Related Colitis

If the event duration or severity is significant, or if it presents with signs of systemic inflammation or acute-phase response (such as elevated C-reactive protein or platelet count or neutrophilia), the following measures are recommended: Perform sigmoidoscopy (or colonoscopy, if applicable) and colon biopsy, take 3-5 pieces of tissue, embed in standard paraffin, and examine infiltrating inflammation and lymphocytes to confirm colitis diagnosis. If possible, freeze 1-2 biopsy specimens. Laboratory investigations should be performed to exclude other etiologies (such as WBC and fecal calprotectin).

Immunotherapy-Related Neurological Toxicity

Participants may experience persistent signs and symptoms of sensory and/or motor neuropathy, which may be related to toripalimab treatment. Diagnostic examinations must be performed to accurately differentiate between other causes.

Ocular Toxicity

Ophthalmologists should evaluate participant visual complaints (such as uveitis or retinal events). Topical corticosteroid eye drops may be used to treat uveitis or superficial scleritis. If immunotherapy-related ocular toxicity is unresponsive to local immunosuppressive therapy, toripalimab treatment should be discontinued completely.

Immunotherapy-Related Bone and Joint Diseases

Drug-related osteoarthritis has been observed, presenting with joint swelling, pain, and limited mobility.

Immunotherapy-Related Hypophysitis

Hormone levels and functional tests (such as TSH, growth hormone, luteinizing hormone, follicle-stimulating hormone, testosterone, prolactin, ACTH levels, and adrenocorticotropic hormone testing) and head MRI will help diagnose hypopituitarism.

Immunotherapy-Related Myocarditis

Immunotherapy-related myocarditis should be suspected when participants present with symptoms and signs of myocarditis, including but not limited to dyspnea, chest pain, palpitations, fatigue, decreased exercise tolerance, and syncope. Immediate evaluation of all suspected myocarditis cases should be performed, monitoring cardiac enzymes, electrocardiography, chest X-rays, echocardiography, cardiac MRI, and cardiology consultation according to professional guidelines. Endomyocardial biopsy may be considered if clinical indicators are available for diagnosis and treatment assistance. Participants with symptoms and signs of myocarditis, in the absence of other clear causes, should be treated according to the guidelines in the investigator's manual.

Table 4 General principles for the management of immunization-related adverse events*

1	A comprehensive evaluation of the subjects was made to determine any alternative causes.
2	In the absence of a clear alternative cause, all inflammatory events should be considered immune-related.
3	Manage according to level, as well as system organs.
4	Early intervention and the early and adequate use of systemic corticosteroids are key in the management, for example, considering the use of prednisone or intravenous equivalent medications for persistent low-grade events or severe events.
5	After initiating corticosteroid therapy, if symptoms do not improve within 2-3 days, dosage escalation may be considered. Once symptoms are controlled to grade 0 or 1, gradual tapering is necessary, with a sufficiently long tapering period (greater than 4 weeks, even 6-8 weeks), to prevent recurrence of immune-related adverse events, especially immune-related pneumonia and hepatitis. When corticosteroid dosage is tapered to ≤10 mg/day prednisone or an equivalent dose, treatment with Toripalimab may be reintroduced. If there is a recurrence of immune-related adverse events, Toripalimab treatment should be permanently discontinued.
6	Systemic corticosteroids are not effective in the treatment of systemic corticosteroids. After discussion with medical guardians, more effective immunosuppressant TNF antagonism should be considered Agents (such as infliximab) or cyclophosphamide, intravenous gamma globulin or mycophenolate mofetil.

*Refer to the NCCN guide

Prior to using Toripalimab for each participant, researchers should consider the balance of benefits and risks. For participants who have met the criteria for permanent treatment discontinuation and are to resume Toripalimab therapy, researchers must thoroughly assess the benefit to the participant and ensure that immune-related adverse events (irAEs) have fully resolved. If treatment is resumed, this decision must be made jointly by the researchers and medical monitors and documented in writing.

For more detailed information regarding management of irAEs, please refer to the researcher's manual. Section

9.7 of Concomitant Medications and Procedures

9.7.1. Recommended Concomitant Medications and Procedures

Antiviral therapy can reduce the risk of developing Hepatocellular Carcinoma (HCC), lower the risk of HBV reactivation, decrease relapse rates, and improve the Overall Survival (OS) and Disease-Free Survival (DFS) of participants with HCC (Freddie 2018, Ge 2015). Therefore, all participants in the HCC cohort who are positive for HBsAg and/or Hepatitis B core antibody and meet the following conditions are allowed to enroll: at enrollment, HBV- DNA should be <2000 IU/mL or 104 copies/mL; if higher than this standard, antiviral therapy should be initiated first to bring it down to normal level for at least 2 weeks and participants must continue antiviral therapy throughout the study. Participants who have previously received HBV antiviral treatment and have normal HBV-DNA levels at enrollment must continue antiviral therapy throughout the study. HCV-RNA positive participants with normal liver function may be included in this study but must undergo approved standard HCV antiviral treatment.

The standard treatment choices recommended by the 2018 Guidelines for the Diagnosis and Treatment of Primary Liver Cancer include Entecavir, Tenofovir or Tenofovir alafenamide. In participants with active HBV infection (HBsAg+, HBeAg+, positive viral load), due to high resistance rates to Lamivudine, it is not recommended. It is advised for participants to continue antiviral treatment for at least 6 months after completing the study treatment.

9.7.2. Permissible Concomitant Medications and Procedures

During this trial, additional medications may be needed to manage participants' conditions, including side effects arising from experimental therapy or disease progression. In general, researchers may administer concomitant medications/treatments deemed necessary for participants. Prophylactic use of antiemetics is not required. If nausea and vomiting occur, participants may use antiemetics after careful consideration by the researchers. Stable doses of anticoagulants are permitted, but participants on Warfarin should undergo PT/INR/PTT monitoring based on clinical indications.

Participants may receive non-oral influenza vaccines. It is recommended to use antidiabetic medications (e.g., metformin, insulin) to control drug-induced hyperglycemia. Before and after intravenous injection of iodine contrast agents, stop using metformin (such as metformin) for 12 hours and 48 hours, as it may cause acute kidney function changes.

Management of treatment-related rashes should be based on standard medical practices and researchers' judgment. Clear documentation should aid in rash classification for subsequent handling. Depending on the severity and type of rash, specialized treatment may be necessary, potentially requiring temporary or permanent discontinuation of the study drug. Rash treatments may include non-occlusive moisturizers, topical or systemic antihistamines (such as diphenhydramine), and/or corticosteroids (considering the risk of hyperglycemia). For dual infections, bacterial cultures should be obtained, and appropriate topical or systemic antibiotics may be considered.

For the treatment of treatment-related mucositis and oral ulcers, early interventions based on standard medical practices and researchers' judgment are recommended. Treatment may include analgesics, topical preparations (suspensions, pastes), and short courses of systemic corticosteroids. More severe cases may require temporary cessation or reduction of medication. Avoid using mouthwashes containing alcohol or peroxide.

Reports have indicated instances of acute renal dysfunction associated with ATG-008 and similar drugs. Generally, this is related to drug-related gastrointestinal toxicity (anorexia, nausea, vomiting, diarrhea, mucositis) leading to dehydration and electrolyte abnormalities. Rehydration, antiemetic, and antidiarrheal treatment should be considered based on clinical

indications. Adjusting the dosage or temporarily stopping ATG-008 may be necessary to manage these toxic reactions.

Reports also associate ATG-008 and related drugs with drug-related pancreatitis, including asymptomatic elevations in amylase and lipase levels, as well as symptomatic pancreatitis. The study protocol includes routine monitoring of amylase and lipase for safety. For participants exhibiting clinical symptoms of pancreatitis (including abdominal pain, nausea, and vomiting), recommendations should be based on medical indications, including intestinal rest and pain management. Adjusting the dose of ATG-008 or temporarily stopping its usage may be necessary to manage these toxic reactions.

Reports have noted cases of drug-related non-infectious acute local pneumonia (mTOR pathway-targeted drug-like effects) associated with ATG-008. Consider this diagnosis for participants showing nonspecific respiratory symptoms and signs such as hypoxia, pleural effusion, cough, dyspnea, or interstitial pneumonia infiltrates after excluding infection, tumors, and other causes through appropriate investigation. Participants with imaging findings suggestive of non-infectious acute local pneumonia with very mild symptoms can continue with ATG-008 without altering the dose. Moderate symptoms may warrant treatment interruption, and corticosteroids may be required. Once symptoms are relieved, ATG-008 can be readministered at the assigned or reduced dosage, depending on the participant's general clinical condition.

Routine preventive measures against infectious diseases are not recommended (except for HBV infection as mentioned in Section 9.7.1). However, the judicious use of antibiotics, antiviral drugs, anti-Pneumocystis drugs, antifungal drugs, or other prophylactic medications may be considered by the researchers during the study period.

Bisphosphonate compounds (such as pamidronate, zoledronic acid) or other medications (such as denosumab) are allowed to prevent or delay bone metastasis progression. A stable dosage regimen should be maintained throughout the study period.

For participants with a history of esophageal variceal bleeding or assessed by the researchers as high-risk for esophageal varices, comprehensive endoscopic treatment following local standards is necessary.

Participants can receive physiologic replacement doses of glucocorticoids (up to 10 mg of hydrocortisone daily) for the maintenance treatment of adrenal insufficiency. To control infusion reactions or immune-related adverse events before the next dose of study drug, systemic corticosteroids must have been reduced for at least 1 month and reached a dosage without immunosuppressive effects (hydrocortisone ≤10 mg/day or equivalent dosage of a similar drug).

All concomitant medications (for treatment and preventive purposes), including blood and blood products, should be reported in the eCRF from the time of participant informed consent signing, throughout the treatment and safety follow-up period (last dose of study drug administered within 90 days).

9.7.3. Prohibited Concomitant Medications and Procedures

The use of other chemotherapy, immunotherapy (including interferons, interleukins), or other anticancer treatments during the study treatment is not allowed. The use of traditional Chinese medicine with anti-tumor and immunostimulant effects, which may lead to unpredictable drug interactions causing or complicating toxicity assessments, is prohibited.

Routine prevention using granulocyte colony-stimulating factors (G-CSF) or granulocyte-macrophage colony-stimulating factors is not allowed. These drugs can only be used for treatment purposes based on standard medical practices and the researcher's judgment when indicated.

Unless symptoms are suspected to be caused by immunological factors, the use of systemic glucocorticoids for any purpose is prohibited. However, after consulting the drug provider, the use of physiologic doses of glucocorticoids (≤10 mg/day of hydrocortisone or equivalent dosage of a similar drug) may be approved.

During the study treatment period, local palliative radiotherapy or surgery for non-target lesions may be considered by the researchers after careful evaluation to treat cancer-related symptoms.

Transcatheter arterial chemoembolization is not allowed during clinical treatment. Live vaccines, including but not limited to measles, mumps, rubella, varicella, yellow fever, seasonal influenza, H1N1 flu, rabies, BCG, and typhoid vaccines, are prohibited.

In vitro metabolism studies have shown that the oxidation metabolism of ATG-008 is mainly catalyzed by CYP3A4/5 (and to a lesser extent CYP2C9). The co-administration of potent CYP3A4 inhibitors (such as ketoconazole, clarithromycin, ritonavir) or inducers (such as rifampin, phenobarbital, phenytoin, carbamazepine) with ATG-008 is prohibited. Consumption of grapefruit-containing foods and beverages is allowed. Appendix 6 provides a list of common drugs belonging to these categories. If drugs metabolized by CYP3A4/5 and CYP2C9 are used in this study, monitoring for drug interactions and enhanced toxicity effects in participants is necessary. All these drugs must be recorded in the eCRF, including their dosages and administration frequencies.

In vitro studies indicate that ATG-008 may moderately activate the pregnane X receptor and moderately induce CYP3A4/5. The in vivo level of induction by ATG-008 is not known, but it may diminish the clinical efficacy of oral or injectable contraceptives. Therefore, participants of childbearing potential must use dual contraception methods throughout the study period.

9.7.4. Required Concomitant Medications and Procedures

Details can be found in Section 9.6.1. Additionally, all participants may receive optimal supportive care (Section 7.3) based on clinical decisions.

This translation includes comprehensive guidelines regarding the administration of concomitant medications and procedures in the context of a medical study involving use of Toripalimab.

9.8. Contraceptive Requirements

During the course of this study, subjects must not become pregnant or father children, as the combined treatment of ATG-008 and Teriparatide monoclonal antibody may affect the fetus. Women should not breastfeed during their participation in this study. Subjects must use contraceptive measures during their participation in this study, which is crucial. Women of childbearing potential must agree to use two effective methods of contraception, with serum pregnancy test results negative at screening. If sexually active with women of childbearing potential, male subjects must use effective barrier contraception.

Effective contraceptive methods include:

1.Combined hormonal contraception that suppresses ovulation (containing estrogen and progestin), including oral, vaginal, and transdermal formulations.

2.Progestin-only hormonal contraception that suppresses ovulation, including oral, injectable, and implantable formulations.

3.Intrauterine devices.

4.Intrauterine hormone-releasing systems.

5.Bilateral tubal ligation.

6.Partner vasectomy (if the partner is the only fertile female partner participating in the trial and has undergone successful surgical evaluation).

7.Total abstinence (defined as avoiding heterosexual intercourse throughout the risk period related to the study treatment. The reliability of abstinence needs to be evaluated based on the duration of the clinical trial and the subject's preferences and daily lifestyle).

The investigator must explain acceptable contraceptive methods to the subjects. Subjects must agree to use the above effective contraceptive methods throughout the entire study period and for 3 months after the last study treatment to be eligible for participation in this study.

9.9. Inventory and Disposition of Investigational Medicinal Products

Investigators or designated personnel at research centers must instruct subjects or caregivers to undergo combined treatment with ATG-008 + Teriparatide monoclonal antibody according to the study protocol requirements. Teriparatide monoclonal antibody needs to be used at the research center. ATG-008 will only be distributed to subjects by authorized research center staff. The doses of all drugs dispensed to subjects and all dose adjustments during the study process must be properly recorded.

ATG-008 and Teriparatide monoclonal antibody must be received by designated personnel at the research center, then stored safely and appropriately in a secure location accessible only to researchers and designated individuals.

Investigators and/or research staff will assess treatment compliance at each subject visit and record it in the eCRF. Additionally, this information must be recorded in the original documents of each subject visit.

Investigators or designated personnel must accurately record the transportation and distribution of investigational drugs in the drug count log. Drug counts will be recorded by study monitors during research center visits and at the end of this study. Subjects will be required to return all unused investigational drugs and packaging regularly, at the end of the study, and/or upon termination of investigational drug therapy. At appropriate times during the study process and at the end of the study, investigators will return all used and unused investigational drugs, drug packaging, drug labels, and a completed copy of the drug count log to the drug provider or designated personnel.

Once the monitor confirms that the drug count is complete, the drugs can be returned or destroyed from the research center.

10. Adverse Events

Adverse events refer to adverse medical events experienced by patients or clinical trial subjects after taking a certain drug, but may not necessarily be causally related to treatment. Therefore, adverse events may include any unfavorable and unexpected signs (including abnormal laboratory results), symptoms, or diseases associated with the duration of the study treatment, regardless of whether they are related to the study treatment.

Symptoms and signs of clinical significance related to disease progression are expected to be reported as adverse events; asymptomatic or study-identified disease progression (non- fatal) confirmed only by examination does not need to be reported as adverse events.

Abnormal laboratory test results occurring during treatment with clinical significance (i.e., meeting one or more of the following conditions) should be recorded as single diagnostic information on the adverse event page of the eCRF:

- Accompanied by clinical symptoms.
- Resulting in changes in the study treatment (such as dose adjustment, interruption of treatment, or permanent termination of treatment).
- Requiring changes in concomitant treatment (such as addition, interruption, termination of concomitant medication, therapy, or any other change).

If laboratory abnormalities are part of a diagnosis or syndrome, only the diagnosis or syndrome should be recorded on the adverse event page of the eCRF. If laboratory abnormalities are not part of a diagnosis or syndrome, they should be recorded as adverse events. If possible, laboratory abnormalities should be recorded in medical terms rather than simply recorded as abnormal laboratory test results (for example, recorded as thrombocytopenia rather than decreased platelet count). Regardless of the severity of laboratory abnormalities, only those that meet severity criteria will be recorded as serious adverse events.

Adverse event reporting period: Adverse events will be recorded from the first administration of the investigational drug; serious adverse events will be recorded from the time the subject signs the screening informed consent form until 30 days after the last dose of

the investigational drug is used. Suspected serious adverse events related to the investigational drug that the investigator becomes aware of at any time after the adverse event reporting period will also be recorded.

Investigators are responsible for recording all adverse events that occur during this study. Adverse event information will be elicited from subjects through non-leading questioning, such as: "Since our last inquiry/your last visit, have you experienced any symptoms or any changes in symptoms?" Adverse events should be recorded on the appropriate page of the eCRF.

The actual level and duration of adverse events should be reported.

The severity of adverse events will be graded using NCI-CTCAE version 5.0 (for details, please visit the NCI-CTCAE website http://ctep.cancer.gov). For adverse events not included in NCI-CTCAE, events will be graded according to the following definitions (semicolon denotes "or"):

Grade 1 - Mild: Asymptomatic or mild symptoms; only clinical or diagnostic observation; no intervention required.

Grade 2 - Moderate: Requires minimal, local, or non-invasive intervention; age-appropriate instrumental activities of daily living limited (instrumental activities of daily living refer to activities such as cooking, shopping, making phone calls, or managing finances).

Grade 3 - Severe or medically significant but not immediately life-threatening; hospitalization or prolonged hospitalization required; disability; basic activities of daily living limited (basic activities of daily living refer to bathing, dressing, eating, toileting, taking medication, and non-bedridden activities).

Grade 4 - Life-threatening consequences; urgent intervention required.

Grade 5 - Death related to the adverse event.

Investigators will assess the relationship between adverse events and investigational drug therapy, as detailed in Table 5 below.

Table 5 Classification of causality of adverse events

It doesn't matter	The event is not temporally related to the investigational treatment, rendering the causal relationship implausible, with alternative medications, interventions, or underlying conditions providing adequate explanations.
Relevant	There is a reasonable possibility of a causal relationship between the event and the investigational treatment, with the likelihood of the event being caused by exposure to the investigational treatment higher than any other medication, intervention, or underlying condition.

10.1. Serious Adverse Events

Serious adverse events refer to adverse medical events resulting in the following outcomes, occurring at any dose level (including events occurring after signing the ICF and before administration):

- Death.
- Life-threatening (immediate risk of death at the time of the event).
- Requires hospitalization (formal admission for medical reasons) or prolongation of existing hospitalization.
- Results in persistent or significant disability/incapacity.
- Results in congenital anomaly/birth defect.

Important medical events that do not result in death, are not life-threatening, or do not require hospitalization may also be considered serious adverse events, provided that, according to appropriate medical judgment, these events endanger the subject and require drug or surgical intervention to prevent one of the outcomes defined in this definition. Such events include allergic bronchospasm requiring intensified treatment in the emergency room or at home, blood dyscrasias not requiring hospitalization, or seizures, or the development of drug dependence or drug abuse.

10.1.1. Events not meeting the definition of serious adverse events

Selective hospitalization for routine treatment or monitoring of the studied indication unrelated to any worsening of the condition is not considered a serious adverse event.

Hospitalization due to the following events is not considered a serious adverse event:

- Routine treatment or monitoring of the studied indication unrelated to any deterioration of the condition.
- Procedures related to the study protocol/disease (e.g., surgery, scans, endoscopies, laboratory sampling, and bone marrow aspirations). However, hospitalization or prolongation of hospitalization due to complications of these procedures is still reportable as a serious adverse event.
- Hospitalization or prolongation of hospitalization due to technical, practice, or social reasons without the occurrence of an adverse event.
- Scheduled procedures (i.e., planned before the initiation of study treatment) must be documented in source documents. However, hospitalization or prolongation of hospitalization due to complications of these procedures is still reportable as a serious adverse event.
- Elective treatment for pre-existing conditions unrelated to the studied indication.

Subjects who die due to disease progression and cannot be classified as any other Grade 5 adverse event are classified as disease progression and reported as serious adverse events. However, "tumor progression"/"disease progression" itself does not need to be reported as an adverse event, and adverse events unrelated to malignant tumor progression (e.g., deep vein thrombosis occurring during disease progression or hemoptysis occurring during disease progression) should be reported according to commonly used guidelines for such events, with causality with the investigational drug appropriately determined.

During the reporting period for adverse events in this study, or when it is judged after the reporting period to be related to the study drug, any subject death that cannot be classified as any other Grade 5 adverse event is classified as NOS (Not Otherwise Specified), and death NOS is reported as an adverse event.

10.2. Reporting of Serious Adverse Events

For each occurrence of a serious adverse event, drug overdose, or pregnancy event in a subject, the principal investigator or designated research center staff must report to the drug provider within 24 hours of becoming aware of the event, in addition to recording it in the subject's original documents and the AE page of the eCRF. The following information must be provided:

1. Protocol number
2. Research center number
3. Subject number
4. Brief description of the event
5. Start date and time
6. Date and time of recovery, if applicable
7. Any medication administered for the event
8. Assessment of causality between the serious adverse event and the investigational drug treatment by the investigator
9. Outcome of the event as of the reporting date

This information will be documented in the protocol-specific event reporting form submitted to the drug provider or regulatory authority. The drug provider is responsible for submitting adverse event reports to the national regulatory authority based on Chinese regulations, Institutional Review Board (IRB)/Independent Ethics Committee (IEC) requirements, and research institution requests.

10.3. Follow-up of Adverse Events and Serious Adverse Events

All adverse events occurring during this study will be followed up in-hospital or via telephone follow-up according to medical practice requirements until they resolve, stabilize, are deemed clinically insignificant by the investigator, or transition to a chronic condition.

Adverse events deemed related to the drug must be followed up until resolution or stabilization.

10.4. Adverse Events and Serious Adverse Events Occurring After the Reporting Period

At the last treatment visit, the investigator should instruct each subject to report any subsequent events they or the investigator believe may be reasonably related to the investigational drug. Serious adverse events or deaths occurring more than 30 days after the end of study treatment and considered reasonably related to the investigational drug should also be reported to the sponsor's drug safety department.

10.5. Drug Overdose

Drug overdose refers to intentional or accidental administration of the investigational drug at levels higher than those specified in the study protocol (including ATG-008 and toripalimab monoclonal antibody). In case of a drug overdose, the investigator and drug provider must be notified immediately, and the subject should be closely monitored for adverse events. If applicable, symptoms caused by drug overdose should be appropriately treated. Drug overdose events and any related adverse events and/or treatments must be documented in the subject's medical records and eCRF. In addition to documenting the overdose event in the subject's medical records and eCRF, the drug provider's drug safety department must be notified of the overdose event within 24 hours of becoming aware of it, and any serious adverse events observed due to overdose must be reported according to serious adverse event requirements.

10.6. Pregnant and Lactating Women

Note: Pregnancy itself is not considered an adverse event; however, discussing pregnancy in this protocol is important because reporting pregnancy events occurring during the study process is crucial, and medical events observed in the mother or fetus/newborn will be classified as adverse events.

Investigators will inform female and male subjects of reproductive potential about the potential risks of pregnancy during participation in the study and advise them to use highly effective contraceptive methods (with low failure rates when used correctly) during drug administration and for at least 3 months after completion of the investigational drug treatment, as described in Section 9.6.

If a subject confirms pregnancy during the study, the investigational drug treatment must be immediately terminated. The investigator must report the pregnancy to the drug provider's drug safety department within 24 hours of becoming aware of the event.

Pregnancy events should be followed up to determine outcomes, including natural or induced miscarriage, birth details, and the presence of any birth defects, congenital abnormalities, or maternal and/or neonatal complications.

Pregnancy events occurring within 3 months after the last dose of study drug must also be reported to the drug provider, regardless of whether the subject received the study drug, whether the subject withdrew from the study, or whether the subject completed the study. Subjects should be instructed to report any pregnancy events to the investigator.

Any serious adverse events occurring during pregnancy must be recorded in the serious adverse event report form (such as serious maternal complications, therapeutic abortions, ectopic pregnancies, stillbirths, neonatal deaths, congenital abnormalities, birth defects), and must be reported within 24 hours according to the serious adverse event reporting procedures.

For pregnancy events in female sexual partners of male subjects, the event must be reported to the drug provider within 24 hours of becoming aware of it, and follow-up should be conducted until the pregnancy outcome. Informed consent should be obtained from the female sexual partner for reporting pregnancy information.

The secretion of study drugs into breast milk is currently unknown, but women should not breastfeed while receiving treatment regimens containing study drugs.

11. Statistical method

11.1. Statistical and analytical plan

This study is an open-label phase study, consisting of two stages: dose escalation and dose expansion.

The dose escalation stage will evaluate the safety and tolerability of ATG-008 in combination with Toripalimab in subjects with advanced solid tumors, assessing DLTs, determining MTD, and RP2D. The optimization and selection of RP2D will be determined by the investigator based on clinical practice. Based on safety and tolerability data from CC-223- ST-001-B Phase I solid tumor subjects, the average dose for the NET group of 47 subjects is approximately 20 mg. Therefore, in the dose escalation stage, if the ATG-008 20 mg QD dose group (ATG-008 in combination with Toripalimab treatment) still shows good safety and tolerability at the end of enrollment, NET cohort subjects will be dosed with ATG-008 20 mg QD in combination with Toripalimab for dose expansion first, and the expansion doses for other cohorts will be determined after the completion of the 30 mg dose group escalation trial and discussion by the SMC.

The dose expansion stage will enroll 5 different solid tumor cohorts at the RP2D dose level, including: advanced HCC, advanced NET, advanced or metastatic gynecological tumors (endometrial cancer, cervical cancer, ovarian cancer), advanced GBM subjects, and advanced solid tumor subjects (approximately 10-12 subjects per cohort), to further evaluate the antitumor efficacy, safety, and tolerability of ATG-008 in combination with Toripalimab treatment in solid tumor subjects. If the ORR for the GBM cohort is approximately greater than 10%, and for other tumor types or cohorts, ORR is approximately >20%, the number of subjects enrolled in that tumor type or corresponding cohort may increase to approximately 40 subjects, with the overall enrollment not exceeding 150 subjects.

11.1.1. General Considerations in Statistics

Formal hypothesis testing will not be conducted for study data, including primary endpoints, secondary efficacy endpoints, demographic, and safety data. Relevant distributions, demographics, baseline, efficacy, and safety parameters will need to be listed. For categorical variables, summary tables will be generated for the number and proportion of subjects in each category (including missing data categories), along with displaying bilateral 95% confidence intervals. For continuous variables, the number of subjects, mean, median, standard deviation, minimum, and maximum values will be displayed. Event occurrence time data will be summarized using the Kaplan-Meier method, with summaries including the 25th percentile, 50th percentile (median), 75th percentile, along with bilateral 95% confidence intervals and the proportion of censoring.

Statistical methods will be detailed in the statistical plan.

11.1.2. Determination of Sample Size

This trial is an open-label dose escalation and dose expansion study, and the sample size is primarily based on clinical considerations, without statistical hypothesis testing.

11.1.3. Subject Distribution

The subject distribution table will include the number of subjects in the analysis population, the number of subjects with evaluable lesions meeting efficacy assessment criteria, the number of subjects lost to follow-up, the number of subjects who exited the study before completion, and the reasons for withdrawal.

11.1.4. Dose Adjustment

According to the guiding principles in Section 9.5.2, the dosing of ATG-008 in combination therapy may be reduced. However, regardless of dose reduction, efficacy data analysis will be based on the mITT and per-protocol populations.

11.2. Analysis Population

• Modified Intent-to-Treat Population (mITT): The population for efficacy analysis will be based on mITT, with subjects defined as those who received at least one dose of the study drug. In this study, the mITT population is consistent with the safety population.

• Per-Protocol Population (PP): Statistical analysis of ORR will also be performed based on the PP population, which will be used to support efficacy analysis. The PP population consists of all subjects from the mITT population who meet the following criteria:

- Compliance with ATG-008 and Toripalimab medication ≥70%;
- Have undergone at least one complete baseline tumor assessment, excluding subjects who died or withdrew from the clinical trial before baseline assessment;
- No major protocol violations affecting the assessment of clinical efficacy.

A list of significant protocol violations affecting statistical analysis will be finalized before database lock.

• Safety Population: Subjects who are enrolled according to the study protocol and have received at least one dose of study treatment will be included in the safety population.

• PK Analysis Population: Subjects defined as those who have received at least one dose of study drug and have undergone PK parameter assessment at least once after baseline.

11.3. Multiplicity

No multiplicity correction will be conducted in this study.

11.4. Data Analysis and Presentation

Summary tables will be generated for the aforementioned distribution data and demographic, baseline, efficacy, and safety data as shown in the following sections. All data collected in the eCRF will be provided in the subject data listings.

11.4.1. Demographic Characteristics

Demographic characteristics, including gender, race, and age at the time of informed consent, will be summarized. Statistical indicators will include the number and proportion of subjects by gender and race. Race categories will be based on recorded categories in the database. For age at the time of informed consent, the mean, median, minimum, maximum, and standard deviation of subject ages will be provided.

11.4.2. Baseline Characteristics and Medical History

Baseline characteristics include performance status, duration since initial diagnosis, efficacy after previous treatments, types of previous treatments, and height/weight. Summary statistics will be performed, and baseline data will be listed; formal hypothesis testing will not be conducted. Additionally, baseline medical history and physical examination results for subjects will be listed.

11.4.3. PK Analysis

PK parameters of ATG-008 when co-administered with Toripalimab will be evaluated, including but not limited to C_{max}, trough concentration (C_{trough}), T_{max}, AUC0-21, AUC0-∞, clearance rate, volume of distribution, and half-life ($t_{1/2}$). PK data will be presented in graphical and/or tabular form, and descriptive summaries will be provided. PK parameters will be presented in tabular form based on ATG-008 dose levels.

11.4.4. Efficacy Analysis

Researchers will assess ORR (Objective Response Rate), DOR (Duration of Response), DCR (Disease Control Rate, defined as CR+PR+SD), PFS (Progression-Free Survival), and OS (Overall Survival) based on RECIST1.1 (excluding GBM) or RANO (for GBM only) criteria. ORR evaluated according to RECIST1.1 or RANO criteria (for GBM only) will serve as the primary efficacy endpoint for the dose expansion phase and will be summarized for all treated subjects. Exploratory efficacy analysis will be conducted for ORR, DOR, DCR, and PFS based on iRECIST criteria for solid tumors (excluding GBM) or iRANO criteria for GBM. Analysis will be conducted in the mITT (modified intention-to-treat) and PP (per- protocol) populations. Kaplan-Meier method in survival analysis will be used to calculate survival functions for events with truncated values (e.g., DOR, PFS, and OS), and 95% confidence intervals will be provided for ORR and DCR.

Analysis will be conducted based on serum ADA detection data to evaluate the potential relationship between teriparatide monoclonal antibody and efficacy.

11.4.4.1. Exposure-Response Analysis

The relationship between ATG-008, M1 exposure, teriparatide monoclonal antibody, and efficacy will be evaluated. Significant trends between drug exposure and response will be determined initially through graphical representation. Based on these findings, appropriate models will be established and simulations conducted to quantify the exposure-response relationship using statistical drug models.

11.4.5. Safety Data Analysis

Safety analysis will be conducted in the safety population. Data from all subjects who received at least one dose of the study drug will be used for safety analysis.

11.4.5.1. Adverse Events

Adverse events will be coded using the Medical Dictionary for Regulatory Activities (MedDRA) and presented in tabular and list formats categorized by MedDRA System Organ Class and Preferred Term. Severity of adverse events will be graded according to CTCAE version 5.0.

Adverse event analysis will be conducted for events deemed to occur during treatment, defined as any adverse event or worsening of pre-existing conditions occurring from the first day of study drug treatment to 30 days after the last dose of study drug. Adverse events with incomplete dates will be assessed using available date information to determine if they are commonly associated with treatment; adverse events with entirely missing dates will be considered as occurring during treatment. Formal hypothesis testing will not be conducted on the incidence rates of adverse events.

Adverse events will be summarized by incidence rate per subject, so each subject will be counted only once for a specified adverse event (Preferred Term) in any table. The number and percentage of subjects experiencing any treatment-related adverse events in each treatment group will be summarized by System Organ Class and Preferred Term. Additionally, the number and percentage of subjects experiencing ≥3 grade treatment-related adverse events will be listed.

Researchers will use "unrelated" and "related" to determine the causality of adverse events with study drug treatment. If a subject experiences the same adverse event repeatedly, the event with the highest severity and/or most commonly associated causality will be used in the table. Serious adverse events will also be listed separately.

All adverse events (occurring during and after treatment), categorized by subject and study date, will be listed in the subject data listing. Additionally, a separate listing will be provided for deceased subjects, serious adverse events, and adverse events leading to discontinuation.

11.4.5.2. Laboratory Examination Data

Clinical laboratory parameter values will be reported in International System of Units (SI). Actual values of clinical laboratory parameters at each study assessment time point and the change from baseline (Day 1, prior to the first dose of study drug) will be summarized, including hematological, clinical biochemical, coagulation, and urine analysis parameters. If there are repeated measurements, the last non-missing value from each study day/time point will be used. If there are no Day 1 data for a subject/parameter, the screening parameter value will be used as the baseline value.

Severity of selected clinical laboratory indicators (those with corresponding CTCAE grading) will be determined using CTCAE standards. Laboratory parameter values with CTCAE grade ≥3 will be provided in the data listing. Additionally, percentage change from baseline for the worst value during the study and change in CTCAE grading relative to baseline for the last parameter value during the study will be generated and categorized for change tables.

11.4.5.3. Vital Signs, Physical Examination, and ECOG Performance Status

Summary of actual values of vital signs at each study visit and their changes relative to baseline (Day 1) will be provided. Additionally, a categorized change table will be generated for the worst values during the study and the final ECOG performance status parameter values relative to baseline percentage changes. Furthermore, summary of physical

examination results at screening and changes during the study will be included. All vital sign measurements, physical examination dates, and ECOG performance status scores will be listed in the subject data listings.

11.4.5.4. Electrocardiogram (ECG) Assessment

ECG results will be descriptively summarized, including heart rate and intervals such as PR, QRS, QT, and QTc (calculated using the Fridericia correction formula). The Fridericia-corrected QTc interval will be calculated as follows: QT / (RR^[1/3]), where RR = 60 / heart rate. Actual values at each study visit and their changes relative to baseline will be reported. ECG data for each subject will be provided in the data listings.

11.4.5.5. Concomitant Medications

Concomitant medications will be coded using the WHO Drug Dictionary. Results will be displayed via anatomical therapeutic classification and preferred term lists. Concomitant medications will include any medications not discontinued before the first dose of study drug administration. If an end date is missing or the medication is still being used, it will be considered a concomitant medication. Subject data listings will include both prior and concomitant medications.

11.4.5.6. Deaths During the Study

All deaths occurring during the entire study period, including within 30 days after the last dose of study drug, will be reported. A table summarizing deaths during the study and their causes will be provided, along with a separate list of deaths during the study.

11.4.6. Exploratory Analysis

Exploratory summaries and analyses will be conducted for ORR, DCR, DOR, and PFS evaluated by the investigators according to iRECIST (excluding GBM) or iRANO (GBM) criteria. Descriptive analysis summaries will be conducted for AFP response rates among HCC subjects, defined as the proportion of subjects meeting AFP response criteria among those with elevated AFP levels at baseline. Ovarian cancer subjects will also be analyzed for CA125 response rates according to GCIG criteria combined with RECIST 1.1. Descriptive analysis summaries will be provided for subjects' levels of MSI, TMB, PD-L1 (tumor tissue), sPD-L1 (blood sample), and the status of pathway mutations related to the study drug (including but not limited to PI3K, AKT, KEAP1, NFE2L2, STK11).

11.4.7. Handling of Missing Data

All missing efficacy data will not be imputed. For time-to-event analyses, subjects without efficacy assessments will be considered censored at time zero. For PFS and DOR, subjects who are not evaluable at the time of disease progression or for the primary or final analysis will be censored at the imaging detection date corresponding to the last efficacy assessment. For OS, subjects will be followed up until they are lost to follow-up, withdraw from the study, or die. Censoring of subjects will also occur at the date of last known survival, regardless of disease status.

Missing dates for adverse events will not be imputed; however, if partial dates are available, they will be used to assess whether adverse events occurred during treatment. Severity of missing adverse events will not be imputed and will be considered missing data in the severity table for each adverse event. If no association between adverse events and study drug treatment is provided, the event will be considered related to the study drug treatment.

11.5. Changes in Study Implementation or Prespecified Analysis

Any deviations from the original statistical analysis plan will be documented in the final clinical summary report.

12. Regulatory, ethical and legal obligations

12.1. Regulatory and ethical compliance

The design, conduct, and reporting of this clinical study will adhere to the principles outlined in the ICH guidelines, GCP, relevant local regulations, and the Helsinki Declaration regarding ethical principles.

12.2. Institutional Review Board/Independent Ethics Committee

The study protocol and proposed Informed Consent Form (ICF) must undergo review and approval by an IRB/IEC before the study commences. Prior to study initiation, the investigator must sign the protocol signature page, confirming their agreement to conduct the study according to the protocol and consenting to the designated agents of the drug provider, IRB/IEC, and regulatory authorities (when necessary) to access all relevant data and records.

12.3. Regulatory Agency Approval

Before initiating the study, the study protocol must obtain approval from the relevant regulatory agencies (if necessary).

12.4. Protocol Compliance

Investigators should ensure to minimize protocol deviations. No exemptions will be granted for protocol deviations in this study. All protocol deviations must be properly documented and reported, and all significant protocol deviations will be recorded in the clinical summary report.

12.5. Protocol Amendments

Any changes or additions to the study protocol can only be made through a written protocol amendment provided by the drug provider and approved by regulatory agencies (if necessary) and IRB/IEC. Amendments involving subject safety may be implemented prior to IRB/IEC approval. Formal protocol amendments require approval before implementation, but in cases involving participant safety, investigators may take immediate actions, even if they deviate from the protocol. In such cases, the drug provider should be notified, and the local IRB/IEC should be informed according to Chinese regulations and local requirements.

12.6. Informed Consent

Eligible subjects can only be enrolled in the study after obtaining written informed consent approved by the IRB/IEC (witnessed, as required by law or regulation).

Informed consent from subjects must be obtained before conducting any study-related procedures (i.e., procedures outlined in the study protocol). The actual date of obtaining subjects' informed consent should be recorded in their eCRF. The investigator will provide a draft version of the ICF suitable for this study and compliant with the ICH-GCP guidelines and regulatory requirements. Any proposed changes to the draft consent form by the investigator must be approved by the drug provider before submission to the IRB/IEC; after IRB/IEC approval, a copy of the approved version must be provided to the drug provider or designated personnel.

12.7. Subject Confidentiality and Disclosure

Investigators must ensure subject anonymity; subjects' names should not appear in documents submitted to the drug provider or its designated personnel. Signed ICFs and subject enrollment logs must be strictly confidential for subject identification at the study center.

12.8. Study Documentation, Recordkeeping, and Document Retention

The study center will retain relevant medical and research records of this clinical trial in accordance with ICH E6 GCP Section 4.9 and relevant regulations and institutional requirements for subject confidentiality. The study center will allow authorized representatives of the drug provider and regulatory agencies to inspect (and copy when required by law) clinical records for quality assurance audits, inspections, and assessments of research safety and progress. Original data refers to all information recorded in the original records or certified copies, including clinical findings, raw data or observed results, and original records necessary for reconstructing and evaluating activities related to the clinical trial.

Data collection will be performed by clinical trial staff at the study center under the supervision of the principal investigator. The study eCRF is the primary data collection tool used in this study. Investigators must ensure the accuracy, completeness, and timeliness of data collected via eCRF and all required reports. Data obtained through eCRF should be

consistent with or explained for inconsistencies with the original documents. All data required on the eCRF must be recorded.

Reasons for missing data must be explained, and data discrepancies in the eCRF will be tracked through the system.

Investigators/institutions must retain the "Essential Documents for Conducting Clinical Trials" (ICH E6 Section 8) and trial documents specified in relevant Chinese regulations and/or guidelines. Measures should be taken to prevent accidental or premature destruction of these documents.

Essential documents (both paper and electronic versions) should be retained for at least 15 years after the end of the clinical trial, unless the drug provider agrees in writing to dispose of these documents or if extension of retention time is required by relevant laws, regulations, and/or guidelines.

12.9. Study Monitoring

The drug provider will appoint a representative to conduct monitoring to ensure that the study center conducts this study in accordance with the study protocol. These monitoring visits may occur before, during, and/or after the study.

This study will be monitored according to GCP principles. Study center monitors will regularly visit the study center.

12.10. Audits and Regulatory Agency Inspections

In addition to routine monitoring procedures, the study center must allow audits by the drug provider or its representatives (such as third parties), and must allow regulatory agency inspections of all study-related documents and materials at the study center to assess compliance with the study protocol and GCP principles. These documents/materials include but are not limited to study center documents, study drugs, medical records/documents of all subjects, all study-related equipment, and the study center responsible for subject handling.

Investigators must agree to allow representatives of the drug provider's regulatory agencies to directly access these documents/materials during and after the study.

Audits or regulatory agency inspections may occur at any time during or after this clinical trial to ensure the validity and integrity of trial data. In the event of significant compliance or regulatory issues, the drug provider may conduct an audit without prior notification to the investigator.

12.11. Information Disclosure

All information provided by the drug provider or its designated personnel to the investigator must be kept strictly confidential. Except for the rights granted to the investigator in the clinical trial protocol, no disclosures shall be made.

Unless required by law, information about this study or the progress of the research shall not be provided to anyone not involved in this study, except to the drug provider or its authorized representatives, or IRB or similar committees.

12.12. Study Termination

Either the investigator or the drug provider may terminate the investigator's participation in this study with written notice submitted for reasonable cause.

The drug provider may terminate this study at any time for any reason with immediate notice, including if the drug provider believes the study must be terminated for subject safety reasons.

12.13. Reporting and Publication of Study Documents

The drug provider will publish key design elements of this study protocol in publicly accessible databases (such as www.clinicaltrials.gov) and data required by regulatory agencies. In addition, upon study completion and finalization of the study report, the results of this study will be submitted for publication and/or disclosed in publicly accessible clinical research results databases.
